# Computational Study of *Ortho*-Substituent Effects on Antioxidant Activities of Phenolic Dendritic Antioxidants

**DOI:** 10.3390/antiox9030189

**Published:** 2020-02-25

**Authors:** Choon Young Lee, Ajit Sharma, Julius Semenya, Charles Anamoah, Kelli N. Chapman, Veronica Barone

**Affiliations:** 1 Department of Chemistry and Biochemistry, Central Michigan University, Mount Pleasant, MI 48859, USA; sharm1a@cmich.edu (A.S.); semen1j@cmich.edu (J.S.); anamo1c@cmich.edu (C.A.); chapm1kn@cmich.edu (K.N.C.); 2 Department of Physics and Science of Advanced Materials Program, Central Michigan University, Mount Pleasant, MI 48859, USA

**Keywords:** antioxidant, dendrimer, electronic effect, hydrogen atom transfer (HAT), single electron transfer-proton transfer (SET-PT), sequential proton loss electron transfer (SPLET), DPPH

## Abstract

Antioxidants are an important component of our ability to combat free radicals, an excess of which leads to oxidative stress that is related to aging and numerous human diseases. Oxidative damage also shortens the shelf-life of foods and other commodities. Understanding the structure–activity relationship of antioxidants and their mechanisms of action is important for designing more potent antioxidants for potential use as therapeutic agents as well as preservatives. We report the first computational study on the electronic effects of *ortho*-substituents in dendritic tri-phenolic antioxidants, comprising a common phenol moiety and two other phenol units with electron-donating or electron-withdrawing substituents. Among the three proposed antioxidant mechanisms, sequential proton loss electron transfer (SPLET) was found to be the preferred mechanism in methanol for the dendritic antioxidants based on calculations using Gaussian 16. We then computed the total enthalpy values by cumulatively running SPLET for all three rings to estimate electronic effects of substituents on overall antioxidant activity of each dendritic antioxidant and establish their structure–activity relationships. Our results show that the electron-donating *o*-OCH_3_ group has a beneficial effect while the electron-withdrawing *o*-NO_2_ group has a negative effect on the antioxidant activity of the dendritic antioxidant. The *o*-Br and *o*-Cl groups did not show any appreciable effects. These results indicate that electron-donating groups such as *o*-methoxy are useful for designing potent dendritic antioxidants while the nitro and halogens do not add value to the radical scavenging antioxidant activity. We also found that the half-maximal inhibitory concentration (IC50) values of 2,2-diphenyl-1-picrylhydrazyl (DPPH) better correlate with the second step (electron transfer enthalpy, ETE) than the first step (proton affinity, PA) of the SPLET mechanism, implying that ETE is the better measure for estimating overall radical scavenging antioxidant activities.

## 1. Introduction

The use of antioxidants to combat oxidative damage caused by excess free radicals is important for food, medical, cosmetics and other industries. Hence, synthesis of new potent antioxidants to tackle specific oxidative problems in these areas is very much needed. Phenolic antioxidants have attracted much attention in recent years, since many natural antioxidants contain the phenolic moiety.

Recently, we reported a new class of phenolic antioxidants that we called dendritic antioxidants. Syringaldehyde and vanillin, very weak natural antioxidants, were used as building blocks to assemble potent phenolic antioxidant dendrimers with half-maximal inhibitory concentration (IC50) values significantly smaller than the building blocks. For example, dendrimers with 4, 6 and 8 syringol units had 27-, 100-, and 170-fold higher scavenging activities, respectively, than the syringaldehyde building block [1,2,3]. Besides enhancing radical scavenging, the dendritic architecture allowed metal chelation, thereby preventing potentially deleterious pro-oxidant effects of the antioxidants. We have also previously shown the ability of these dendritic antioxidants to protect biomolecules such as DNA and low-density lipoproteins from radical damage. Dendritic antioxidants offer several advantages similar to dendrimers. For example, their size, solubility, chelation ability, and antioxidant activity may be precisely manipulated by using appropriate cores and building blocks. Therefore, understanding the structure–activity relationship of dendritic antioxidants and their mechanism of action is paramount.

Substituents, such as electron-donating groups (EDGs) and electron-withdrawing groups (EWGs) on the phenol ring significantly affect the antioxidant activity of a phenolic antioxidant [3,4,5]. Several computational and experimental studies suggest that EDGs (e.g., OH, NH_2_, OCH_3_, CH_3_) decrease O–H bond dissociation enthalpy (BDE) of the phenol, which implies increased antioxidant activity [6,7,8,9,10]. On the other hand, EWGs showed varying results. Some computational studies reveal that EWGs, such as NO_2_, CN, CF_3_, F, Cl, and Br increase BDE of the phenol (lower antioxidant activity) [9,10]. However, experimental studies on halogens report different activities depending on the position relative to the phenol OH or the number of halogen substituents. For example, 6-chromanol with di-*o*-Cl substitution showed lower galvinoxyl radical scavenging activity but better than mono-*o*-Cl and tri-Cl substitution (two *o*-Cl groups and one *m*-Cl) compared to the unsubstituted 6-chromanol [5]. Hydroxycinnamic acids with mono-*o*-Br showed no effect in 2,2-diphenyl-1-picrylhydrazyl (DPPH) radical scavenging activity [4].

Antioxidant action for free radical scavenging can occur in several different ways, but the ultimate outcome is the donation of a hydrogen atom (H•) to the radical. The hydrogen atom transfer can occur in multiple different ways. It can be (1) direct H• atom transfer to radicals, (2) electron transfer, followed by H^+^ loss, or (3) proton loss, followed by electron transfer. The proposed mechanism for (1) is the hydrogen atom transfer (HAT) mechanism in which the hydrogen atom (H•) is transferred from the phenolic OH to the radical. In the HAT mechanism, the BDE is the most important parameter in estimating the antioxidant activity. The mechanism for (2) is the single electron transfer-proton transfer (SET-PT), in which an electron is first transferred from PhOH to the radical. The PhOH then becomes a phenoxy radical cation (PhOH•^+^), which in turn deprotonates during the second step to form a phenoxy radical (PhO•). In the SET-PT mechanism, the radical transfer in the 1st step is measured as the ionization potential (IP) and the deprotonation in the 2nd step corresponds to the proton dissociation enthalpy (PDE). The proposed mechanism for (3) is the sequential proton loss electron transfer (SPLET), which also proceeds via two steps. The 1st step involves the deprotonation of PhOH, forming a phenoxide ion (PhO^−^). Subsequently, the PhO^−^ ion transfers an electron to the radical and becomes a phenoxy radical (PhO•). The enthalpy of the 1st step is denoted as the proton affinity (PA) and the 2nd step corresponds to the electron transfer enthalpy (ETE). HAT has been reported to be the most favorable antioxidant mechanism in non-polar solvents or in gas phase, while SET-PT and SPLET are the more prevalent mechanisms in polar solvents [9,10,11,12,13,14,15]

Computational studies of the electronic effects on dendritic antioxidants with multiple free radical scavenging sites have not been reported before. In order to study the effects of EDGs and EWGs on dendritic antioxidants, we performed computational studies using the Density Functional Theory (DFT) calculations for a series of small phenol-based dendritic antioxidants (in methanol), each containing either an EDG or an EWG that is *ortho* to the phenolic OH group.

## 2. Materials and Methods

All calculations were performed using Gaussian 16 [16]. Geometry optimizations as well as frequency calculations were obtained using the B3LYP functional and the 6-311++G** basis set. Several conformations were used as initial structures to find the minimum energy conformation due to the flexibility of the studied compounds. All structures have been relaxed without symmetry constraints until maximum forces and displacements were smaller than 4.5 × 10^−4^ Hartree/Bohr and 1.8 × 10^−3^ Bohr, respectively. The requested convergence of the root mean square (RMS) variation of the density matrix elements and the total energy per cell in the self-consistent field procedure is 10^−8^ a.u. and 10^−6^ Hartree, respectively. To model the solvent effect of methanol, the Polarizable Continuum Model/Solvation Model Density (PCM/SMD) from Truhlar et al. was used in all calculations, including geometry optimizations [17].

For the different mechanisms studied here, the enthalpies are defined as follows:

1. Bond Dissociation Enthalpy: H(PhO•) + H(H•) − H(PhOH)
2. Ionization Potential: H(PhOH•^+^) + H(e^−^) − H(PhOH)
3. Proton Dissociation Enthalpy: H(PhO•) + H(H^+^) − H(PhOH•^+^)
4. Proton Affinity: H(PhO^−^) + H(H^+^) − H(PhOH)
5. Electron Transfer Enthalpy: H(PhO•) + H(e^−^) − H(PhO^−^)

From these definitions, it becomes clear that in order to find the total enthalpies of each mechanism we need the enthalpies for the electron, proton, and the H atom in methanol. As reported before, the solvation enthalpy of the H atom in most organic solvents is about 5 kJ/mol and we therefore adopted this value for methanol [17]. Methanol solvation enthalpies of the proton (−1071 kJ/mol) and the electron (−77 kJ/mol) have been calculated here at the same level of theory as the main compounds (B3LYP/6-311++G**). These values are obtained by performing the calculation of a methanol molecule in methanol (PCM/SMD model) and then the methanol molecule after addition of a proton (or addition of an electron) in methanol (PCM/SMD model) as indicated by:

6. Proton solvation enthalpy: H(H^+^(CH_3_OH)(methanol)) − H(H^+^(gas)) − H((CH_3_OH)(methanol))
7. Electron solvation enthalpy: H(CH_3_OH^−^ (methanol)) − H(e^−^(gas) − H(CH_3_OH(methanol))

where the enthalpy of a proton in the gas phase is taken as $\frac{5}{2}RT$.

## 3. Results and Discussion

### 3.1. Geometry Optimization

**Figure 1:**
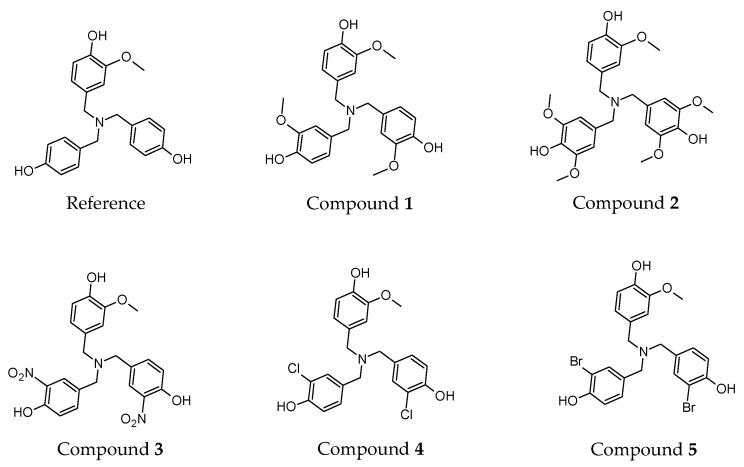
Structures of reference and target compounds **1–5**.

For each compound in Figure 1, we performed geometry optimizations. Structural relaxation of all compounds was carried out to find their minimum energy conformation as detailed under Materials and Methods. Our calculations indicate that in the optimized lowest energy conformation, the three phenol rings of each dendritic antioxidant were almost perpendicular to each other, indicating the absence of π-π stacking interaction between the phenol rings and no steric hindrance between the *ortho*-substituents.

The *o*-OCH_3_ group of the common phenol ring (in the reference and compounds **1**–**5**) was oriented in the same plane as the aromatic ring. It has been reported that in such an orientation, the 2p-type lone pair of electrons on the oxygen is parallel to the aromatic p-orbitals, thereby efficiently overlapping the aromatic π-electron cloud of PhOH [18]. This orientation stabilizes the incipient phenoxy radical (PhO•) and weakens the OH bond of PhOH [18]. The methyl of the OCH_3_ groups orients away from the OH group and the H of the phenol-OH points towards the oxygen of *o*-OCH_3_, forming an intramolecular hydrogen bond. If there are two OCH_3_ groups *ortho* to the phenolic OH (such as in compound **2**), both groups are in the same plane as the phenol ring with the methyl groups orienting away from the OH. It was also observed that *o*-NO_2_, *o*-Br, and *o*-Cl groups formed an H-bond with the H of phenolic OH in the energy-minimized conformation.

### 3.2. Enthalpy Calculations

To the best of our knowledge, this is the first computational study on the mechanism of action of dendritic antioxidants with multiple free radical scavenging sites.

**Figure 2:**
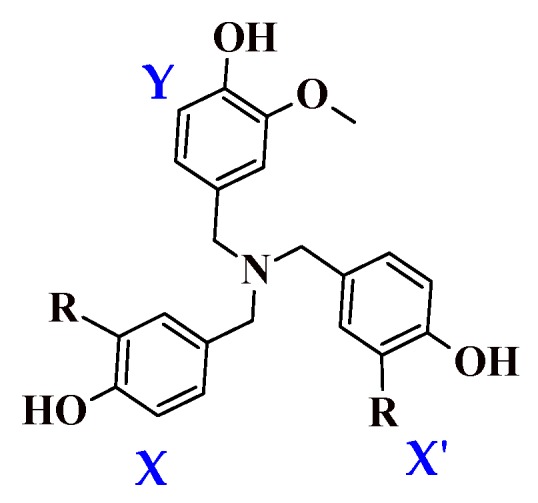
General structure of the compounds and notation (X, X’ and Y) for the phenol rings. R = H, electron-donating or electron-withdrawing group.

We determined the enthalpy for each individual ring present on the dendritic antioxidants. To distinguish the enthalpy of each phenol O–H in the target compounds, X, X’ and Y notations were used (Figure 2). X and X’ represent equivalent phenol rings with either an EDG or EWG *ortho* to the phenolic OH. Y denotes the common ring with one *o*-OCH_3_ group.

In this study, only compound **2** contains PhOH with two *ortho* substituents (OCH_3_). The enthalpy of **2** can be affected by not only electronic but also steric effects. The additional OCH_3_ group might help stabilize the incipient phenoxy radical electronically but might also confer a slight negative impact via steric effect. Since all other compounds have only one *ortho* substituent on each ring, a direct comparison of compound **2** with others might not be fair, but to get further insights into the effect of multiple electron-donating groups on antioxidant activity, we decided to design the compound and calculate its enthalpy nonetheless.

In most computational studies involving the electronic effects of substituents on antioxidant activities, the 1st step of each proposed mechanism, BDE (HAT), IP (1st step of SET-PT), and PA (1st step of SPLET), was reported to be the thermodynamically significant [9,10,11]. In some cases, the 2nd step of SET-PT and SPLET is not even calculated. To understand the antioxidant mechanisms (in methanol) more thoroughly, we calculated the enthalpy of the energy minimized structure of each dendritic molecule for all steps in the three proposed mechanisms (HAT, SET-PT, and SPLET) at the B3LYP/6-311G** level of theory. For the HAT mechanism (Table 1), we calculated the enthalpy for each ring independently as if only one ring in the molecule reacted with the radical: three values in the first column, one for the common phenol ring with *o*-OCH_3_ (Y) and the other two for the two equivalent phenol rings (X, X’) where R is either an *o*-EDG or *o*-EWG. In the second column of each compound, the enthalpy values for the two equivalent phenol rings (X, X’) were averaged. In the case of SET-PT, only one value is given for each step for each compound (Table 2) since the loss of an electron is a global property and will most likely occur at the phenol ring with the highest electron density in an antioxidant with multiple phenol OH groups. For SPLET (Table 3 and Table 4), we present three values in the first column and two in the second column as we did for HAT (calculated raw data is shown in Table S1 in Supplementary Information).

#### 3.2.1. HAT Mechanism

In MeOH, *o*-OCH_3_ (EDG) decreased BDE_O–H_ whereas *o*-NO_2_ (EWG) increased BDE_O–H_. *Ortho*-halogens (Cl and Br) showed similar BDE_O–H_ compared to the unsubstituted reference (Table 1).

#### 3.2.2. SET-PT Mechanism

The IP values of all compounds (except for **2**) are most likely derived from their common phenol ring (Y) with one *o*-OCH_3_ because the ring has the highest electron density. In the case of compound **2**, its IP value could be derived from either of the two phenol rings (with two *o*-OCH_3_) since they are electron richer than the phenol ring with one *o*-OCH_3_.

Unexpectedly, compound **2** showed higher IP and lower PDE values than the reference and compound **1** (Table 2). Since **2** is the only one with two *o*-substituents, direct comparison with others might be misleading. Without considering compound **2**, *o*-OCH_3_ (EDG) decreased IP whereas EWGs (*o*-NO_2_, *o*-Cl, and *o*-Br) increased IP. The *ortho*-OCH_3_ group increased PDE whereas the *o*-NO_2_, *o*-Cl, and *o*-Br groups decreased PDE compared to the reference. It should be noted that the differences in IP and PDE values between the compounds are very small, implying they might be derived from the same ring, probably Y.

#### 3.2.3. SPLET Mechanism

Our results show that *o*-OCH_3_ (EDG) slightly increased PA whereas *o*-NO_2_ (EWG) significantly decreased PA (Table 3). *Ortho*-OCH_3_ decreased ETE considerably while *o*-NO_2_ increased ETE substantially compared to the reference (Table 4). *Ortho*-halogens (*o*-Cl and *o*-Br) exhibited slightly lower PA and slightly higher ETE values compared to the unsubstituted reference.

### 3.3. Thermodynamically Favorable Antioxidant Mechanism for Dendritic Antioxidants

Thermodynamically favorable processes should be determined by calculating the change in the free energy of a reaction (∆G). The entropy component (−T∆S) in the proposed mechanisms is negligible, meaning that ∆G largely depends on the enthalpy (∆H). Therefore, it is reasonable to determine the most preferred mechanism from enthalpy values [9,11].

**Scheme 1:**
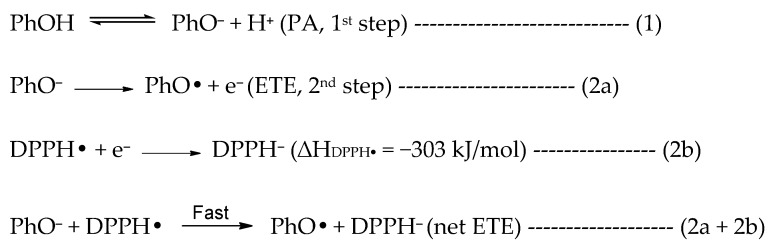
Reaction between antioxidant (PhOH) and the DPPH radical via the SPLET mechanism.

In methanol, the SET-PT is clearly the least favored mechanism due to the large enthalpy of the first step. The SPLET mechanism has an overall low enthalpy in the first step but the enthalpy of the second step is comparable to the BDE in the HAT mechanism. However, the second step in SPLET is not isolated and occurs simultaneously with the reduction of the radical (in this case DPPH•) (reaction 2a + 2b in Scheme 1) as stated in the original papers that introduced the SPLET mechanism [13,19]. The authors state that based on studies between phenolic antioxidant and the DPPH radical in hydroxylic solvents like methanol or ethanol, the deprotonation step (1st step of SPLET) is equilibrium (reaction 1 in Scheme 1) and electron transfer from PhO^−^ to the DPPH radical is fast (reaction 2a + 2b in Scheme 1) [13,14,19]. Many studies consider the first step as thermodynamically significant, perhaps based on these reports, and thus the enthalpy of only the 1st step is used to determine the major operating mechanism or in assessing the antioxidant potential. However, we wish to include the role of the DPPH radical in determining the major antioxidant mechanism. If we consider the electron transfer from PhO^−^ (reaction 2a, Scheme 1) to the DPPH radical (reaction 2b, Scheme 1) to be concurrently occurring in the second step, the overall enthalpy of the second step can be reduced by −303 kJ/mol (reaction 2b), thus significantly favoring the SPLET mechanism over HAT in methanol. We note that the enthalpy for DPPH reduction was obtained at the same level of theory as all other calculations following reaction 2b (Scheme 1).

Our computational study indicates that although both HAT and SPLET mechanisms are possible, SPLET is the more prevalent mechanism for dendritic antioxidants in the presence of the DPPH radical in methanol. This result is consistent with previous studies reporting that SPLET is the major operating mechanism in polar ionizing solvents, while HAT is more favorable in nonpolar solvents or in gas [9,10,11,13,14]. However, it should be cautioned that the SPLET process between dendritic antioxidants and DPPH• in methanol should not be extrapolated to other radicals or solvents.

Values of pKa depend on the PA of phenol O–H: the higher the PA, the more difficult it is to deprotonate, meaning a higher pKa. Based on the pKa values calculated using the ChemAxon pKa calculator (Table S2 in Supplementary Information), the phenol rings in the dendritic antioxidants have pKa values of less than 11.0, indicating that they are weakly acidic. It was shown that phenols with pKa values below 12.5 can undergo the SPLET process with radicals derived from molecules with low pKa values, e.g., DPPH• and ROO• [19].

### 3.4. Prediction of Overall Antioxidant Activity of Dendritic Antioxidants

**Figure 3:**
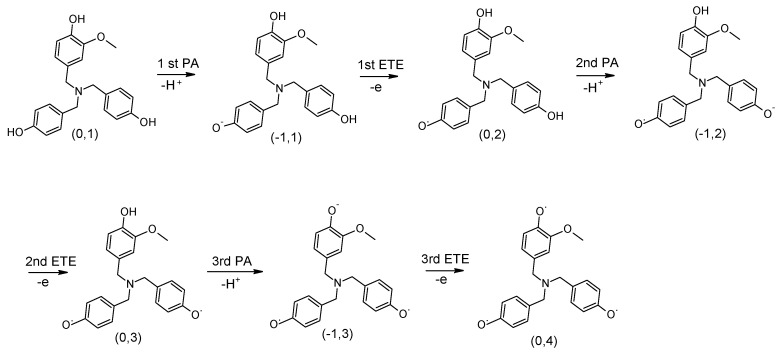
Scheme of the reference compound undergoing consecutive sequential proton loss electron transfer (SPLET) mechanisms. The order was determined based on the computed PA values shown in Table 3. At each step, charge and spin multiplicity of the resulting molecule are indicated in parenthesis (charge, spin multiplicity).

Since our dendritic antioxidants have three potential radical scavenging sites, it is important to know whether all three phenol rings can undergo the SPLET mechanism and contribute to the antioxidant activity or whether their enthalpy becomes too high after one or two radical scavenging and stops. Hence, we determined the cumulative total enthalpy (ΔH_cum_) using SPLET as the major operating mechanism in methanol to estimate the overall antioxidant activity of our dendritic compounds. Starting from the phenol ring which had the lowest PA value based on the PA order determined in the SPLET mechanism (Table 3), H^+^ was removed and then an electron was removed immediately after to form a PhO• radical. The process was repeated based on the PA enthalpy order of the PhOH rings and continued cumulatively until all three PhOH rings became PhO• radicals. Figure 3 shows a scheme of the changes in the reference compound after each step. The ΔH_cum_ of each compound was determined by adding the PA values of steps 1–3 and the ETE values of steps 1–3. These cumulative enthalpy calculations enable us to determine potential enthalpy cooperative effects of the rings in the dendritic antioxidants.

In the SPLET mechanism, the 1st step corresponds to the PA. EWGs decrease PA whereas EDGs increase PA. This means PhO^−^ formation occurs more readily with PhOH containing an EWG and the resulting PhO^−^ will undergo fast e-transfer to the radical. This may lead to the misconception that a PhOH with an EWG is a better antioxidant than the one with an EDG (higher PA). Based on our experience, antioxidants containing EDGs are better antioxidants than those containing EWGs. In our laboratory, syringaldehyde (4-hydroxy-3,5-dimethoxybenzaldehyde) and vanillin (4-hydroxy-3-methoxybenzaldehyde), each containing an aldehyde group, were used to synthesize dendritic antioxidants via reductive amination. Syringaldehyde and vanillin are very weak antioxidants by themselves, but dendrimers derived from them showed significantly higher antioxidant activities (as determined by the DPPH assay [20]). For example, antioxidant dendrimers containing eight syringaldehyde or vanillin derivatized units showed over 170- and 70-fold increase in DPPH radical scavenging activity rather than 8-fold compared to syringaldehyde and vanillin starting material, respectively [3]. The dramatic increase was caused in part by the replacement of the electron-withdrawing aldehyde (in the starting material) with an electron-donating benzyl group (in the dendrimer). Based on this observation, EDGs are more beneficial than EWGs for antioxidant activity. Although EDGs contribute to increasing PA in polar solvents, they will decrease ETE, thus helping electron transfer from the antioxidant to the radical occur more efficiently. Hence, we argue that the 2nd step (ETE) needs to be considered in determining the full antioxidant potential of our dendritic antioxidants since EDGs reduce ETE considerably. In addition, to emulate each phenol ring reacting with a radical (DPPH• was used in this study), the enthalpy change of the DPPH radical that is undergoing reduction by an electron from PhOH ring was considered. Therefore, the net enthalpy of the 2nd step of each PhOH ring was determined by combining ETE of PhOH (reaction 2a in Scheme 1) with ∆H of DPPH•, which is −303 kJ/mol (reaction 2b in Scheme 1).

In the reference compound, the phenol rings with no substituent (X and X’) had a lower PA than the common ring (Y) and thereby are expected to lose H^+^ before the common ring. Thus, the H^+^ was removed from one of the phenol rings with no substituent (X) and formed a PhO^−^ ion. The 1st PA enthalpy was 150 kJ/mol. In turn, an electron was removed to form a PhO• radical. The 1st ETE was 355 kJ/mol to which the ETE of DPPH• (−303 kJ/mol) was added, giving a net ETE of 52 kJ/mol. The process was repeated for the 2nd phenol ring (X’) to determine the 2nd PA and ETE, which were 147 kJ/mol and 56 kJ/mol, respectively. The 3rd PA and ETE, which originate in the common ring, were 128 kJ/mol and 61 kJ/mol, respectively.

Compound **1** has the same three phenol rings with one *o*-EDG (OCH_3_) on each ring. Each phenol ring underwent the SPLET process one by one. The 1st, 2nd, and 3rd PA/ETE were 154/30, 150/37, 147/41 kJ/mol, respectively. Our results show a slight decrease in PA values as more H^+^ is removed. In contrast, ETE increases as the reaction progresses.

In the case of compound **2**, the first H^+^ was removed from one of the rings with two *o*-OCH_3_ (X or X’), because it had a lower PA than the common ring (Y). The 1st, 2nd, and 3rd PA/ETE were 151/16, 149/19, 148/40 kJ/mol, respectively. Its ETE values are substantially lower than those of the reference compound and **1**. By looking at the order of overall ETE values (compound **2** < compound **1** < reference compound), we can see the importance of EDG on the electron-donating potential of the antioxidants.

In compound **3**, the two phenol rings containing *o*-NO_2_ had the lowest PA values. Therefore, the first proton was removed from one of the PhOH rings with NO_2_. The 1st PA was determined to be 116 kJ/mol. Then, an electron was removed to form a PhO• radical (1st ETE = 119 kJ/mol). The same process was repeated for the 2nd phenol ring with the NO_2_ group. The 2nd PA and ETE were 116 kJ/mol, and 116 kJ/mol, respectively. The common ring underwent SPLET last. Its PA and ETE were 69 kJ/mol and 119 kJ/mol, respectively. Calculations were also done for compounds **4** and **5** to determine their PA and ETE in the same manner. The 1st, 2nd, and 3rd PA/ETE of compound **4** were 132/66, 130/70, 114/73 kJ/mol, respectively. The 1st, 2nd, and 3rd PA/ETE of compound **5** were 136/61, 133/66, 118/70 kJ/mol, respectively (calculated raw data is shown in Table S3 in Supplementary Information).

**Figure 4:**
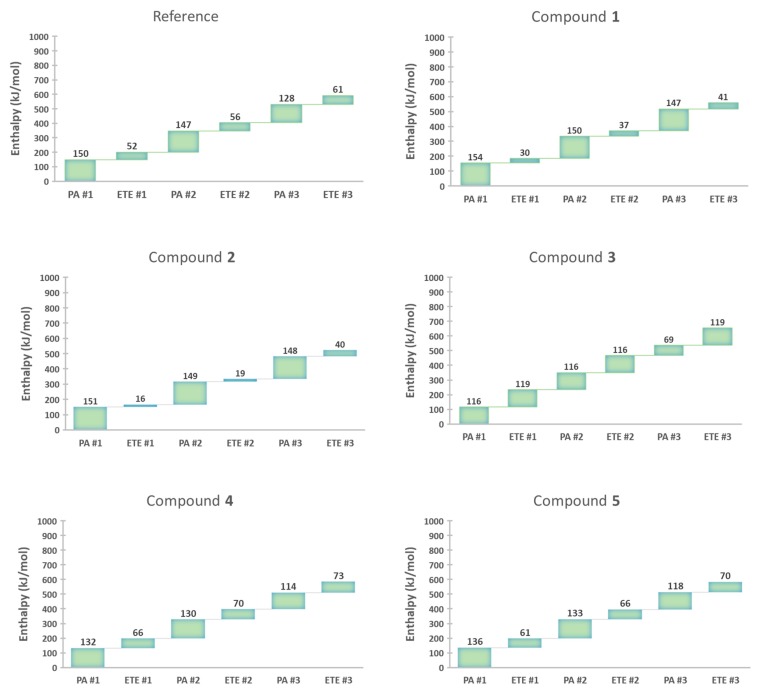
Enthalpy value of each step in a multiple SPLET process for the compounds studied. Each ETE value has been decreased by 303 kJ/mol (ΔH_DPPH•_ = −303 kJ/mol) to account for the reaction with the DPPH radical.

Figure 4 summarizes how each step affects the enthalpy of the following step, according to DFT. Overall, PA values gradually decrease while ETE values increase as more and more phenol rings undergo radical scavenging. This means that the enthalpy values in the later steps are affected by the phenol ring(s), which underwent deprotonation and ETE earlier. None of the steps showed a significant increase in the corresponding enthalpy values, suggesting that the multiple SPLET process can occur and all three PhOH rings in our dendritic antioxidants are able to scavenge free radicals.

**Figure 5:**
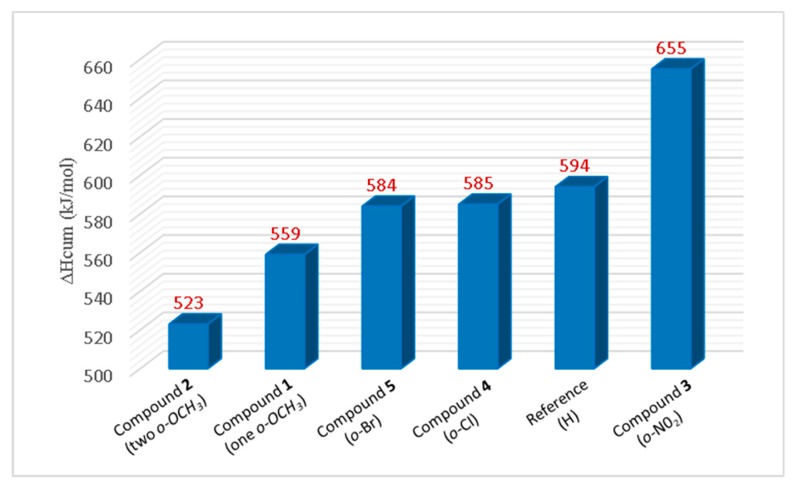
The cumulative total enthalpy (ΔH_cum_) of consecutive SPLET mechanisms.

The ΔH_cum_ of each compound, determined by cumulatively adding PA and ETE of all three rings with consideration of the ∆H_DPPH•_ is shown in Figure 5. The overall enthalpy order is compound **2** (523 kJ/mol) < compound **1** (559 kJ/mol) < compound **5** (584 kJ/mol) ≈ compound **4** (585 kJ/mol) < reference compound (594 kJ/mol) < compound **3** (655 kJ/mol).

If the ∆H_DPPH•_ is not considered, the overall ΔH_cum_ order is compound **2** (1432 kJ/mol) < compound **1** (1468 kJ/mol) < compound **5** (1493 kJ/mol) ≈ compound **4** (1494 kJ/mol) < reference compound (1503 kJ/mol) < compound **3** (1564 kJ/mol). It is worth mentioning that the cumulative total enthalpy values (ΔH_cum_) are slightly higher than the summed-up enthalpy (ΔH_tot_ = PA in Table 3 + ETE in Table 4) of the three rings in each compound: compound **2** (1428 kJ/mol) < compound **1** (1462 kJ/mol) < compound **5** (1490 kJ/mol) = compound **4** (1490 kJ/mol) < reference compound (1496 kJ/mol) < compound **3** (1561 kJ/mol). Nonetheless, the order remains the same. The difference in the ΔH_cum_ and ΔH_tot_ indicates that the phenol rings in the dendritic antioxidants are mutually dependent on each other.

Based on these results, compound **2** (two *o*-OCH_3_) had the lowest total enthalpy, followed by **1** (one *o*-OCH_3_), suggesting that PhOH with EDG will have higher antioxidant activity compared to the unsubstituted PhOH; the more EDG on PhOH, the better the antioxidant. Compound **3** (containing *o*-NO_2_) showed a higher total enthalpy compared to the reference compound. This suggests that PhOH with EWG will have poorer antioxidant activity than the unsubstituted PhOH. Compounds containing *o*-halogens (Cl and Br) had slightly lower enthalpy than the reference. However, the enthalpy values are very close to each other, suggesting that halogens do not have a significant effect on the overall antioxidant activity.

In order to determine if the dendritic antioxidants composed of 3 phenol rings are more active radical scavengers than their starting materials and fragments, we determined PA, ETE and ΔH of vanillin and syringaldehyde (starting materials) as well as the fragments of compound **1**, 4-(aminomethyl)-2-methoxyphenol (Y’) and 4-methyl-2-methoxyphenol (Y”) and fragments of compound **2**, 4-(aminomethyl)-2,6-dimethoxyphenol (X’) and 4-methyl-2,6-dimethoxyphenol (X”) (Table 5).

Compound **1** has three rings derived from vanillin. Therefore, the PA and ETE of vanillin were tripled (3 equivalents) and then added together to determine the total enthalpy (ΔH). ΔH of vanillin (1518 kJ/mol) was higher than that (1468 kJ/mol) of compound **1**. In the case of compound **2**, it can be considered to be derived from 2 equivalents of syringaldehyde (X) and 1 equivalent of vanillin (Y). Therefore, both PA and ETE were calculated by using the formula, 2X+Y, giving a PA of 356 kJ/mol and an ETE of 1128 kJ/mol. The total enthalpy (ΔH) of the starting material (1484 kJ/mol) was higher than that of compound **2** (1432 kJ/mol). The higher enthalpy values of the starting materials are likely due to the presence of the electron-withdrawing aldehyde group on both molecules.

Both compounds **1** and **2** had lower PA but higher ETE and ΔH, compared to their respective fragments. This trend suggests that radical scavenging of antioxidants with multiple radical scavenging sites is a multistep cumulative process, which results in a gradual decrease in PA and increase in ETE as more radical scavenging sites are used up. The higher ΔH of dendritic antioxidants is understandable because their radical scavenging is a cumulative process. By the time the 2nd and 3rd phenol rings undergo the SPLET process, the antioxidant already has one phenoxy and two phenoxy radicals, respectively, which are higher in energy than the ground state (neutral phenol).

### 3.5. Correlation of Enthalpy Values with DPPH IC50

**Figure 6:**
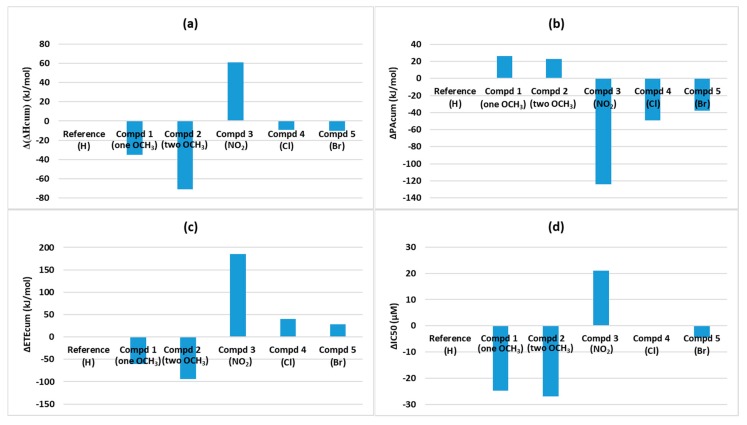
Graphical representation of the enthalpy deviation of each compound from the value of the reference compound: (**a**) ∆(∆H_cum_), (**b**) ∆PA_cum_, (**c**) ∆ETE_cum_ and (**d**) ∆IC50.

It is important to know how well theoretically computed enthalpy values of dendritic antioxidants correlate with their experimental DPPH radical scavenging activities [21]. We first determined the cumulative total PA (PA_cum_) and ETE (ETE_cum_) by adding up the PA and ETE of steps 1–3 shown in Figure 4, respectively. Then, PA_cum_, and ETE_cum_ were added to determine ∆H_cum_ (Table 6). To see the enhancing/decreasing effect, the enthalpy differences between each compound and the reference were determined (∆(∆H_cum_), ∆PA_cum_, and ∆ETE_cum_) and ∆IC50 values were determined the same way. These differences are depicted in Figure 6. Based on these graphs, we observe that the trend of ∆IC50 resembles that of ∆(∆H_cum_) most closely, followed by ∆ETE_cum_.

**Figure 7:**
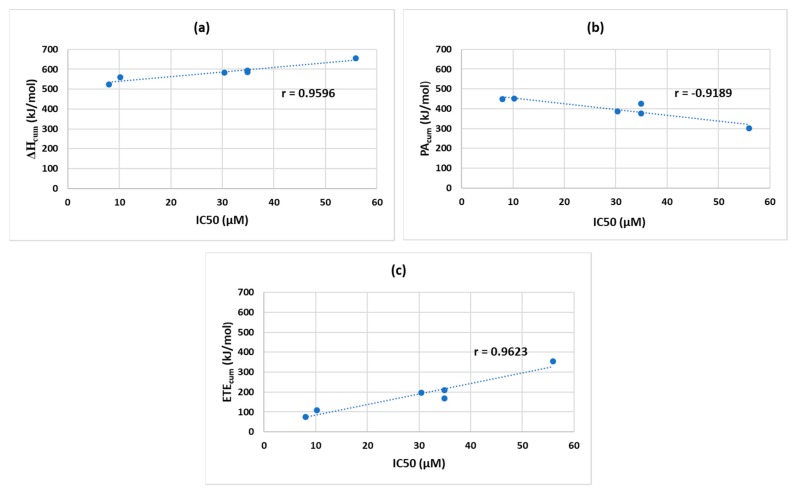
Correlation between IC50 and ∆H_cum_ (**a**), IC50 and PA_cum_ (**b**), and IC50 and ETE_cum_ (**c**).

We also determined the Pearson correlation coefficients (r) between the IC50 and ∆H_cum_/ PA_cum_/ ETE_cum_ values to determine which parameter of the SPLET mechanism better correlates with the IC50 values. Although the IC50 order looks slightly different from the order of ∆H_cum_ (Table 6), their correlation was quite impressive: r = 0.9596 with a 95% confidence interval (0.6692, 0.9957) based on Fischer’s Z-transformation (Figure 7a). The PA_cum_ showed a strong negative correlation with IC50: r = −0.9189 with a 95% confidence interval (−0.9912, −0.4222) (Figure 7b). The r of IC50 to ETE_cum_ was 0.9623 with a 95% confidence interval (0.6882, 0.9960) (Figure 7c). 

These r values, based on the collected data, indicate that the IC50 correlates better with ∆H_cum_ and ETE_cum_ as opposed to PA_cum_. These results suggest that between ETE and PA, the ETE might be a more significant measure in estimating the antioxidant potential than PA. From our study, it is clear that *o*-EDGs like OCH_3_ increase antioxidant activity whereas *o*-EWGs such as NO_2_ decrease it and the trend is more consistent with ETE. Some studies reported compounds containing EWGs to be better antioxidants in the SPLET mechanism than their counterparts with EDGs because of the lower PA values obtained from EWG containing compounds [11]. Their interpretation is reasonable if we consider the findings in the original papers that proposed the SPLET mechanism: the 1st step (PA) of SPLET is equilibrium and PhOH is converted to PhO^−^ with the help of ionizing solvents. Once, the PhO^−^ is formed in the 1st step, the 2nd step (ETE) occurs rapidly in the presence of DPPH radicals [13,14]. It was also reported that rate constants of PhOHs/DPPH•reactions in alcohols were increased by the addition of a base, indicating that PhO^−^ formation is important in SPLET [19]. The formation of PhO^−^ is directly related to the acidity of the PhOH: the more acidic PhOH is, the faster PhO^−^ forms. Based on these reports, phenols containing EWGs seem to be better antioxidants than unsubstituted ones and those containing EDGs. This is because phenols with EWGs can form PhO^−^ more readily and enter the SPLET mechanism faster. The comparison of IC50 values between our *o*-NO_2_ (55 µM) and *o*-OCH_3_ (8 µM) containing antioxidants (both having similar intramolecular and intermolecular H-bonding properties in ionizing solvents like methanol) suggest that faster formation of PhO^−^ (lower PA of *o*-NO_2_) does not necessarily result in better antioxidant activities. The negative effect of *o*-NO_2_ on the antioxidant activity was shown not only by our study but also other experimental studies [22]. Thus, we propose that the 2nd step (ETE) of SPLET should be considered in assessing the full antioxidant potential of phenolic antioxidants. In agreement with our proposal, Li et al. showed that the introduction of *o*-NH_2_ will be more beneficial for the antioxidant activity than *o*-NO_2_ although their *o*-NO_2_ containing compound had lower PA value than the *o*-NH_2_ counterpart [10].

## 4. Conclusions

Enthalpy values for model dendritic antioxidants with either an *o*-EDG or *o*-EWG were determined for each step of the three proposed mechanisms (HAT, SET-PT and SPLET) in methanol using DFT calculations. Based on our results, *o*-OCH_3_ (*o*-EDG) decreases BDE, IP (1st step of SET-PT), and ETE (2nd step of SPLET) and increases PDE (2nd step of SET-PT) and PA (1st step of SPLET) while *o*-NO_2_ (EWG) showed the opposite effects. SPLET was found to be the preferred mechanism in methanol when DPPH• was used as the radical.

To compare relative antioxidant activity between the dendritic compounds, the total enthalpy of each compound was determined by cumulatively running SPLET for all three PhOH rings and adding all the enthalpy values. The results showed that *o*-OCH_3_ (EDG) increases the antioxidant activity whereas *o*-NO_2_ (EWG) decreases it. In comparison, *ortho*-halogens (Br and Cl) showed negligibly lower enthalpy values compared to the unsubstituted PhOH. Correlation of the DPPH radical scavenging activities with each step in SPLET revealed that the experimental IC50 values better correlate with ETE (the 2nd step) rather than PA (the 1st step), implying that ETE is a more important factor in estimating the role of substituents on antioxidant activity than PA.

## Figures and Tables

**Table 1:** Calculated O–H bond dissociation enthalpy (BDE) values (kJ/mol) of the phenol rings (X, X’, and Y) in methanol.

Compound	Reference (H)		Compound 1 (one OCH_3_)		Compound 2 (two OCH_3_)		Compound 3 (NO_2_)		Compound 4 (Cl)		Compound 5 (Br)	
Phenol ring	BDE	<BDE>	BDE	<BDE>	BDE	<BDE>	BDE	<BDE>	BDE	<BDE>	BDE	<BDE>
X	345	345	328	329	311	311	379	377	342	342	342	342
X’	345		329		311		375		342		341	
Y	328	328	330		329	329	330	330	329	329	330	330

**Table 2:** Calculated ionization potential (IP) and proton dissociation enthalpy (PDE) values (kJ/mol) in methanol.

Compound	Reference (H)	Compound 1 (one OCH_3_)	Compound 2 (two OCH_3_)	Compound 3 (NO_2_)	Compound 4 (Cl)	Compound 5 (Br)
IP	430	427	440	448	439	437
PDE	57 (Y) ^a^	60	30 (X/X’) ^a^	41 (Y) ^a^	50 (Y) ^a^	51 (Y) ^a^

^a^ In parenthesis, we indicated the ring where the proton dissociation occurs.

**Table 3:** Calculated proton affinity (PA) values (kJ/mol) of the phenol rings (X, X’ and Y) in methanol.

Compound	Reference (H)		Compound 1 (one OCH_3_)		Compound 2 (two OCH_3_)		Compound 3 (NO_2_)		Compound 4 (Cl)		Compound 5 (Br)	
Phenol ring	PA	<PA>	PA	<PA>	PA	<PA>	PA	<PA>	PA	<PA>	PA	<PA>
X	150	150	153	154	151	151	116	117	132	132	136	136
X’	150		155		151		119		132		137	
Y	152	152	154		152	152	152	152	152	152	152	152

**Table 4:** Calculated electron transfer enthalpy (ETE) values (kJ/mol) of the phenol rings (X, X’ and Y) in methanol.

Compound	Reference (H)		Compound 1 (one OCH_3_)		Compound 2 (two OCH_3_)		Compound 3 (NO_2_)		Compound 4 (Cl)		Compound 5 (Br)	
Phenol ring	ETE	<ETE>	ETE	<ETE>	ETE	<ETE>	ETE	<ETE>	ETE	<ETE>	ETE	<ETE>
X	355	354	333	334	319	319	422	419	370	369	364	364
X’	354		333		319		415		368		364	
Y	335	335	334		336	336	337	337	336	336	337	337

**Table 5:** PA, ETE and ΔH of starting materials and fragments of compounds **1** and **2**.

Molecule		PA (kJ/mol)	ETE (kJ/mol)	ΔH (kJ/mol)
Compound **1**		451	1017	1468
Starting material	Vanillin (Y)	120	386	1518 ^a^
Fragment 1	4-(Aminomethyl)-2-methoxyphenol (Y’)	151	335	1458 ^b^
Fragment 2	4-Methyl-2-methoxyphenol (Y”)	155	328	1449 ^c^
Compound **2**		448	984	1432
Starting Material	Syringaldehyde (X)	118	371	1484 ^d^
Fragment 1	4-(Aminomethyl)-2,6-dimethoxyphenol (X’)	152	317	1424 ^e^
Fragment 2	4-Methyl-2,6-dimethoxyphenol (X”)	155	310	1413 ^f^

^a^ ΔH = 3 equiv. PA (Y) + 3 equiv. ETE (Y), ^b^ ΔH = 3 equiv. PA (Y’) + 3 equiv. ETE (Y’), ^c^ ΔH = 3 equiv. PA (Y”) + 3 equiv. ETE (Y”), ^d^ ΔH = 2 equiv. PA (X) + 1 equiv. PA (Y) + 2 equiv. ETE (X) + 1 equiv. ETE (Y), ^e^ ΔH = 2 equiv. PA (X’) + 1 equiv. PA (Y’) + 2 equiv. ETE (X’) + 1 equiv. ETE (Y’), ^f^ ΔH = 2 equiv. PA (X”) + 1 equiv. PA (Y”) + 2 equiv. ETE (X”) + 1 equiv. ETE (Y”).

**Table 6:** The cumulative enthalpy values, ∆H_cum_, PA_cum_, and ETE_cum_, for dendritic antioxidants and the experimental DPPH IC50 values.

Compound	∆H_cum_(kJ/mol)	PA_cum_ (kJ/mol)	ETE_cum_ (kJ/mol)	IC50 (µM) ^a^
Reference compound	594	425	169	34.9
Compound **1** (one OCH_3_)	559	451	108	10.2
Compound **2** (two OCH_3_)	523	448	75	8.0
Compound **3** (NO_2_)	655	301	354	55.9
Compound **4** (Cl)	585	376	209	34.9
Compound **5** (Br)	584	387	197	30.4

^a^ Data from [21].

## Supplementary Materials

The following are available online at https://www.mdpi.com/2076-3921/9/3/189/s1. Table S1. DFT calculations in methanol; Table S2. Values of pKa obtained using ChemAxon pKa calculator; Table S3. Multistep DFT calculations in methanol.
