# Supplementary material for: Computational Study of Ortho-Substituent Effects on Antioxidant Activities of Phenolic Dendritic Antioxidants
Source: Antioxidants (Basel). 2020 Feb 25;9(3):189. doi: 10.3390/antiox9030189 (PMC7139565; doi:10.3390/antiox9030189)
Supplement: Supplementary file 1 [file antioxidants-09-00189-s001.pdf]

Supplementary Information: Computational Study of *Ortho*-Substituent Effects on Antioxidant Activities of Phenolic Dendritic Antioxidants

Choon Young Lee[1,*], Ajit Sharma[1], Julius Semenya[1], Charles Anamoah[1], Kelli N. Chapman[1] and Veronica Barone[2,*]

[1] Department of Chemistry and Biochemistry, Central Michigan University, Mount Pleasant, Michigan 48859, USA
[2] Department of Physics and Science of Advanced Materials Program, Central Michigan University, Mount Pleasant, Michigan 48859, USA
[*] Correspondence: lee1cy@cmich.edu (C.Y.L.); baron1v@cmich.edu (V.B.)

Table of Contents

S1. DFT calculations in methanol: **2**

S1.0 - Reference Compound **2**

S1.1 - Compound 1 .... **10**

S1.2- Compound 2 .... **20**

S1.3-Compound 3 .... **31**

S1.4-Compound 4 .... **40**

S1.5- Compound 5 .... **49**

S2. Values of pKa obtained using ChemAxon pKa calculator **58**

S3. Multistep DFT calculations in methanol: **59**

S3.0 - Reference **59**

S3.1- Compound 1 .... **63**

S3.2 - Compound 2 .... **68**

S3.3 - Compound 3 .... **73**

S3.4 - Compound 4 .... **78**

S3.5 - Compound 5 .... **82**

S1. DFT calculations in methanol:

Information ordered as follows:

Molecule (Charge, spin multiplicity), Enthalpies (au), lowest frequencies (cm^{-1}), and, optimized geometries (Cartesian, Å) of Compounds **1** to **5** as well as reference obtained at the B3LYP/6-311++G**

S1.0 - Reference Compound

ArOH (0,1)

Sum of electronic and thermal Enthalpies= -1207.754766

Frequencies -- 14.0287 22.2730 26.6668

Center Number	Atomic Number	Atomic Type	Coordinates (Angstroms) X	Y	Z
1	7	0	0.545298	-0.002838	-1.059804
2	6	0	1.750102	0.800269	-1.344702
3	6	0	1.775420	2.142082	-0.643979
4	6	0	1.747246	3.339929	-1.365814
5	6	0	1.858147	2.221222	0.751796
6	6	0	1.797251	4.578184	-0.726861
7	6	0	1.905407	3.448891	1.407252
8	6	0	1.875209	4.631399	0.663955
9	8	0	1.922012	5.872491	1.254205
10	6	0	0.729951	-1.399489	-1.500524
11	6	0	1.713054	-2.184530	-0.658590
12	6	0	1.431254	-2.483014	0.681961
13	6	0	2.913450	-2.658322	-1.194541
14	6	0	2.315221	-3.220565	1.462132
15	6	0	3.811919	-3.402258	-0.427362
16	6	0	3.510618	-3.681149	0.904627
17	8	0	4.350449	-4.406590	1.716447
18	6	0	-0.643700	0.594049	-1.697005
19	6	0	-1.959487	0.028568	-1.204692
20	6	0	-2.335515	0.189069	0.139680
21	6	0	-2.832215	-0.625723	-2.072433
22	6	0	-3.553462	-0.300535	0.596606
23	6	0	-4.061641	-1.116644	-1.620623
24	6	0	-4.425125	-0.957984	-0.293213
25	8	0	-5.633078	-1.441210	0.144490
26	8	0	-4.022936	-0.202140	1.880236
27	6	0	-3.205807	0.448244	2.862506
28	1	0	2.612129	0.214854	-1.015617
29	1	0	1.868684	0.955227	-2.430804
30	1	0	1.686222	3.309154	-2.449286
31	1	0	1.890189	1.310128	1.340366
32	1	0	1.972991	3.494516	2.489941
33	1	0	1.980742	5.782254	2.215167
34	1	0	-0.247557	-1.885001	-1.445904
35	1	0	1.044208	-1.437643	-2.557489
36	1	0	0.502634	-2.137105	1.124185
37	1	0	3.157247	-2.446401	-2.230967
38	1	0	4.740280	-3.763308	-0.859223

39	1	0	5.136020	-4.679178	1.223076
40	1	0	-0.618985	1.665814	-1.483813
41	1	0	-0.594612	0.488392	-2.794109
42	1	0	-1.668889	0.701524	0.821613
43	1	0	-2.558862	-0.758051	-3.113914
44	1	0	-4.741711	-1.626106	-2.294696
45	1	0	-5.731085	-1.235960	1.086455
46	1	0	-3.027814	1.492631	2.591920
47	1	0	-3.771060	0.402410	3.792202
48	1	0	-2.253380	-0.074597	2.985066
49	1	0	1.774921	5.500569	-1.296870
50	1	0	2.087529	-3.450006	2.497463

----

1. ArO-/X (-1,1)

Sum of electronic and thermal Enthalpies= -1207.289832

Frequencies -- 17.5945 22.8240 26.3824

Center Number	Atomic Number	Atomic Type	Coordinates (Angstroms) X	Y	Z
1	7	0	0.507680	0.349673	-1.008751
2	6	0	1.052485	1.704204	-1.260098
3	6	0	0.318737	2.821256	-0.554182
4	6	0	-0.471955	3.743259	-1.253478
5	6	0	0.419318	2.997282	0.835418
6	6	0	-1.135230	4.785894	-0.610472
7	6	0	-0.236826	4.030406	1.494950
8	6	0	-1.049174	4.975513	0.798857
9	8	0	-1.665584	5.950709	1.413341
10	6	0	1.438622	-0.685304	-1.497230
11	6	0	2.700060	-0.820195	-0.670525
12	6	0	2.643516	-1.273323	0.655000
13	6	0	3.956889	-0.528538	-1.207087
14	6	0	3.795123	-1.424593	1.419974
15	6	0	5.124134	-0.676627	-0.455275
16	6	0	5.040106	-1.124217	0.861997
17	8	0	6.148831	-1.291949	1.658486
18	6	0	-0.818449	0.193856	-1.632883
19	6	0	-1.580090	-1.031718	-1.172867
20	6	0	-1.968117	-1.155436	0.172056
21	6	0	-1.937323	-2.037634	-2.069312
22	6	0	-2.689104	-2.263515	0.601442
23	6	0	-2.666357	-3.153596	-1.645102
24	6	0	-3.041557	-3.271570	-0.316728
25	8	0	-3.755649	-4.370287	0.094009
26	8	0	-3.119016	-2.488438	1.883166
27	6	0	-2.809751	-1.516436	2.891094
28	1	0	2.093597	1.688028	-0.927566
29	1	0	1.072145	1.909739	-2.345189
30	1	0	-0.569163	3.644622	-2.332427
31	1	0	1.031695	2.309607	1.414127
32	1	0	-0.132595	4.140031	2.571573
33	1	0	0.902968	-1.637794	-1.476934

34	1	0	1.712830	-0.499421	-2.550158
35	1	0	1.683063	-1.516138	1.097788
36	1	0	4.032945	-0.178492	-2.231896
37	1	0	6.092728	-0.445061	-0.887680
38	1	0	6.948614	-1.057843	1.168321
39	1	0	-1.397451	1.085244	-1.379423
40	1	0	-0.734865	0.172360	-2.733357
41	1	0	-1.703332	-0.376634	0.875868
42	1	0	-1.647856	-1.958466	-3.111881
43	1	0	-2.943954	-3.937475	-2.341501
44	1	0	-3.938800	-4.289621	1.042066
45	1	0	-3.259308	-0.549176	2.650760
46	1	0	-3.241459	-1.901977	3.813575
47	1	0	-1.728189	-1.407512	3.008604
48	1	0	-1.738119	5.483057	-1.186910
49	1	0	3.741998	-1.778683	2.443804

----

2. ARO-/X' (-1,1)

Sum of electronic and thermal Enthalpies= -1207.289710

Frequencies -- 12.1053 22.4971 24.2067

Center Number	Atomic Number	Atomic Type	Coordinates (Angstroms) X	Y	Z
1	7	0	0.548462	-0.084669	-1.056800
2	6	0	1.628864	0.851617	-1.418401
3	6	0	1.512667	2.210636	-0.761266
4	6	0	1.291196	3.367974	-1.515213
5	6	0	1.652683	2.350858	0.625192
6	6	0	1.207860	4.624709	-0.916576
7	6	0	1.569722	3.597220	1.240640
8	6	0	1.346213	4.737870	0.465829
9	8	0	1.256956	5.995079	1.017023
10	6	0	0.889697	-1.467028	-1.466373
11	6	0	2.019717	-2.096898	-0.684780
12	6	0	1.854214	-2.482323	0.655385
13	6	0	3.269448	-2.349866	-1.266344
14	6	0	2.878348	-3.081368	1.379790
15	6	0	4.306968	-2.951444	-0.557245
16	6	0	4.157374	-3.345620	0.803522
17	8	0	5.120480	-3.916560	1.478827
18	6	0	-0.728475	0.336251	-1.661834
19	6	0	-1.946205	-0.361355	-1.091772
20	6	0	-2.285684	-0.190160	0.260906
21	6	0	-2.768551	-1.149310	-1.895316
22	6	0	-3.418093	-0.799293	0.788642
23	6	0	-3.912097	-1.761898	-1.372039
24	6	0	-4.239624	-1.591173	-0.036787
25	8	0	-5.364044	-2.193387	0.471562
26	8	0	-3.845655	-0.701853	2.087181
27	6	0	-3.071579	0.078143	3.008099
28	1	0	2.568910	0.389171	-1.108589
29	1	0	1.684855	0.981718	-2.513275

30	1	0	1.182333	3.290497	-2.592625
31	1	0	1.834304	1.473686	1.237649
32	1	0	1.684557	3.690062	2.316308
33	1	0	1.375769	5.946572	1.975438
34	1	0	-0.017397	-2.062893	-1.335728
35	1	0	1.131561	-1.495087	-2.543648
36	1	0	0.896347	-2.313140	1.141783
37	1	0	3.434861	-2.071458	-2.304819
38	1	0	5.262076	-3.133917	-1.043349
39	1	0	-0.829153	1.411332	-1.491251
40	1	0	-0.710578	0.190378	-2.755716
41	1	0	-1.657723	0.424121	0.893612
42	1	0	-2.522140	-1.292798	-2.942041
43	1	0	-4.552155	-2.376168	-1.996117
44	1	0	-5.445854	-1.964327	1.409607
45	1	0	-3.027314	1.124048	2.692091
46	1	0	-3.590473	0.006249	3.962848
47	1	0	-2.060591	-0.326247	3.107290
48	1	0	1.035957	5.514991	-1.511669
49	1	0	2.713456	-3.371841	2.414384

----

3. ARO-/Y (-1,1)

Sum of electronic and thermal Enthalpies= -1207.288916

Frequencies -- 14.2495 20.5819 21.5475

Center Number	Atomic Number	Atomic Type	Coordinates (Angstroms) X	Y	Z
1	7	0	0.503290	-0.202123	-1.047105
2	6	0	1.923688	0.091784	-1.312746
3	6	0	2.452440	1.304650	-0.575879
4	6	0	2.902311	2.435745	-1.264939
5	6	0	2.537694	1.314161	0.821987
6	6	0	3.419397	3.542504	-0.592602
7	6	0	3.047967	2.411740	1.510733
8	6	0	3.490934	3.529432	0.799411
9	8	0	4.008406	4.640824	1.423290
10	6	0	0.154783	-1.557767	-1.510734
11	6	0	0.759098	-2.668380	-0.678274
12	6	0	0.373140	-2.856027	0.656547
13	6	0	1.694217	-3.555662	-1.217842
14	6	0	0.903037	-3.885524	1.427217
15	6	0	2.235011	-4.596455	-0.460337
16	6	0	1.838054	-4.758464	0.865712
17	8	0	2.332333	-5.761591	1.666700
18	6	0	-0.369950	0.811782	-1.681950
19	6	0	-1.812574	0.772395	-1.232111
20	6	0	-2.161765	1.148133	0.081854
21	6	0	-2.842021	0.395090	-2.092062
22	6	0	-3.481275	1.137174	0.510095
23	6	0	-4.175387	0.383208	-1.668461
24	6	0	-4.562755	0.747258	-0.357342
25	8	0	-5.801903	0.743219	0.053941

26	8	0	-3.877327	1.500986	1.783996
27	6	0	-2.875679	1.912755	2.712585
28	1	0	2.499032	-0.784119	-1.002232
29	1	0	2.101835	0.219051	-2.394710
30	1	0	2.850081	2.456293	-2.349125
31	1	0	2.204317	0.449356	1.386284
32	1	0	3.110783	2.402355	2.594634
33	1	0	4.011817	4.512629	2.381634
34	1	0	-0.934102	-1.637997	-1.469672
35	1	0	0.443531	-1.697645	-2.567026
36	1	0	-0.358783	-2.189730	1.101342
37	1	0	2.009920	-3.437956	-2.249854
38	1	0	2.960435	-5.277298	-0.895075
39	1	0	2.952100	-6.309697	1.166373
40	1	0	0.059276	1.788099	-1.441031
41	1	0	-0.331080	0.713399	-2.781145
42	1	0	-1.375285	1.453199	0.762292
43	1	0	-2.607510	0.105196	-3.113227
44	1	0	-4.961296	0.085810	-2.358041
45	1	0	-2.346102	2.804392	2.360577
46	1	0	-3.404169	2.149539	3.636295
47	1	0	-2.153781	1.111818	2.904038
48	1	0	3.764667	4.414338	-1.137553
49	1	0	0.594966	-4.025944	2.457661

----

4. ARO./X (0,2)
Sum of electronic and thermal Enthalpies= -1207.125445
Frequencies -- 14.8435 21.2317 24.6060

Center Number	Atomic Number	Atomic Type	Coordinates (Angstroms) X	Y	Z
1	7	0	0.550166	0.038835	-1.045214
2	6	0	1.743392	0.838836	-1.351085
3	6	0	1.793538	2.155420	-0.619926
4	6	0	2.052722	3.356172	-1.321991
5	6	0	1.627372	2.203819	0.788805
6	6	0	2.133841	4.559403	-0.666304
7	6	0	1.704482	3.389806	1.469671
8	6	0	1.958306	4.632734	0.769305
9	8	0	2.027186	5.733472	1.384021
10	6	0	0.723105	-1.356300	-1.499161
11	6	0	1.735896	-2.139456	-0.692972
12	6	0	1.494491	-2.452983	0.652184
13	6	0	2.925730	-2.595087	-1.267367
14	6	0	2.407626	-3.188015	1.399854
15	6	0	3.852929	-3.336324	-0.532764
16	6	0	3.591751	-3.630691	0.804432
17	8	0	4.460798	-4.354240	1.585445
18	6	0	-0.648501	0.651794	-1.656181
19	6	0	-1.958009	0.073727	-1.165006
20	6	0	-2.327702	0.208851	0.183943
21	6	0	-2.831509	-0.569550	-2.040385

22	6	0	-3.540520	-0.295839	0.637989
23	6	0	-4.055262	-1.075787	-1.591243
24	6	0	-4.412444	-0.943411	-0.259094
25	8	0	-5.614176	-1.442252	0.175804
26	8	0	-4.004801	-0.222623	1.924659
27	6	0	-3.188357	0.416793	2.914819
28	1	0	2.621167	0.258489	-1.043666
29	1	0	1.845731	1.015313	-2.434149
30	1	0	2.181374	3.315907	-2.398666
31	1	0	1.442187	1.281167	1.326227
32	1	0	1.585151	3.433815	2.546806
33	1	0	-0.251824	-1.841694	-1.413422
34	1	0	1.002151	-1.387390	-2.565632
35	1	0	0.574813	-2.121437	1.123162
36	1	0	3.137318	-2.371741	-2.308426
37	1	0	4.772230	-3.683971	-0.993894
38	1	0	5.235348	-4.614095	1.068319
39	1	0	-0.620367	1.719007	-1.417677
40	1	0	-0.608755	0.571357	-2.755096
41	1	0	-1.661457	0.713473	0.871980
42	1	0	-2.562614	-0.681367	-3.085357
43	1	0	-4.735967	-1.576898	-2.270829
44	1	0	-5.707790	-1.257558	1.122536
45	1	0	-3.017667	1.466442	2.660536
46	1	0	-3.750348	0.352548	3.845362
47	1	0	-2.232511	-0.102423	3.025805
48	1	0	2.324833	5.484158	-1.199765
49	1	0	2.211743	-3.429721	2.438846

----

5. ARO./X' (0,2)

Sum of electronic and thermal Enthalpies= -1207.125489

Frequencies -- 17.8773 20.2611 23.7756

Center Number	Atomic Number	Atomic Type	Coordinates (Angstroms) X	Y	Z
1	7	0	0.612134	-0.116872	-1.050863
2	6	0	1.912015	0.528456	-1.337851
3	6	0	2.106757	1.855268	-0.637287
4	6	0	2.189648	3.051670	-1.357714
5	6	0	2.236532	1.919250	0.755640
6	6	0	2.389070	4.274555	-0.719285
7	6	0	2.434427	3.131936	1.410553
8	6	0	2.509180	4.313788	0.669212
9	8	0	2.704534	5.540330	1.258179
10	6	0	0.625091	-1.515751	-1.498356
11	6	0	1.475163	-2.417860	-0.641863
12	6	0	1.309575	-2.439480	0.767575
13	6	0	2.416156	-3.291776	-1.235548
14	6	0	2.046871	-3.284990	1.553304
15	6	0	3.170152	-4.148766	-0.472997
16	6	0	3.021094	-4.183224	0.967001
17	8	0	3.709479	-4.964817	1.680816

18	6	0	-0.498525	0.625737	-1.679830
19	6	0	-1.869942	0.204051	-1.198017
20	6	0	-2.240301	0.413359	0.141312
21	6	0	-2.795043	-0.368321	-2.069755
22	6	0	-3.504443	0.050290	0.590092
23	6	0	-4.070016	-0.733385	-1.625614
24	6	0	-4.427617	-0.529248	-0.302697
25	8	0	-5.679221	-0.891511	0.126807
26	8	0	-3.973793	0.208505	1.867114
27	6	0	-3.099900	0.771073	2.854836
28	1	0	2.693360	-0.162565	-1.009913
29	1	0	2.045082	0.666155	-2.423778
30	1	0	2.097160	3.031403	-2.439130
31	1	0	2.188357	1.007999	1.342801
32	1	0	2.537720	3.165483	2.490838
33	1	0	2.774917	5.443872	2.217881
34	1	0	-0.404299	-1.890001	-1.448239
35	1	0	0.940352	-1.602558	-2.550779
36	1	0	0.580705	-1.776936	1.219280
37	1	0	2.541623	-3.272370	-2.313110
38	1	0	3.897919	-4.815969	-0.921777
39	1	0	-0.348648	1.682385	-1.445622
40	1	0	-0.457106	0.533374	-2.777908
41	1	0	-1.532631	0.864282	0.825306
42	1	0	-2.526855	-0.534139	-3.107718
43	1	0	-4.790546	-1.180163	-2.302044
44	1	0	-5.760989	-0.685936	1.070294
45	1	0	-2.806980	1.788612	2.582086
46	1	0	-3.675211	0.791152	3.779211
47	1	0	-2.211251	0.148015	2.987710
48	1	0	2.450761	5.196256	-1.287396
49	1	0	1.919843	-3.313047	2.630090

----

6. ARO./Y (0,2)

Sum of electronic and thermal Enthalpies= -1207.131942

Frequencies -- 14.2681 21.5845 22.1957

Center Number	Atomic Number	Atomic Type	Coordinates (Angstroms) X	Y	Z
1	7	0	0.519407	0.031448	-1.059429
2	6	0	1.795775	0.740070	-1.283464
3	6	0	1.916306	2.038811	-0.516214
4	6	0	1.963382	3.271323	-1.176352
5	6	0	2.011354	2.040208	0.881190
6	6	0	2.097834	4.468990	-0.475698
7	6	0	2.144063	3.226683	1.597605
8	6	0	2.187236	4.445283	0.915299
9	8	0	2.322101	5.648376	1.566330
10	6	0	0.582633	-1.354133	-1.574406
11	6	0	1.498105	-2.264035	-0.784994
12	6	0	1.179603	-2.644048	0.526291
13	6	0	2.673779	-2.771664	-1.344813

14	6	0	2.005843	-3.491156	1.257084
15	6	0	3.513989	-3.624388	-0.627575
16	6	0	3.179096	-3.980901	0.677971
17	8	0	3.963983	-4.812931	1.440021
18	6	0	-0.595316	0.753500	-1.684864
19	6	0	-1.960121	0.327478	-1.198477
20	6	0	-2.212955	0.182714	0.171437
21	6	0	-3.010521	0.127863	-2.135613
22	6	0	-3.480770	-0.155049	0.620895
23	6	0	-4.272044	-0.206250	-1.723908
24	6	0	-4.585949	-0.368198	-0.326370
25	8	0	-5.737447	-0.672420	0.078571
26	8	0	-3.827952	-0.311873	1.904421
27	6	0	-2.837124	-0.120234	2.932040
28	1	0	2.594973	0.066424	-0.966453
29	1	0	1.951027	0.935100	-2.357897
30	1	0	1.896414	3.300091	-2.259474
31	1	0	1.987634	1.099779	1.422234
32	1	0	2.221720	3.212624	2.680537
33	1	0	2.382884	5.506699	2.520919
34	1	0	-0.433849	-1.755816	-1.538893
35	1	0	0.889102	-1.359209	-2.633744
36	1	0	0.267799	-2.276021	0.985070
37	1	0	2.943962	-2.499731	-2.360394
38	1	0	4.424240	-4.009400	-1.076732
39	1	0	4.747653	-5.080963	0.940797
40	1	0	-0.479506	1.817077	-1.447292
41	1	0	-0.560495	0.674275	-2.783787
42	1	0	-1.405587	0.343507	0.872346
43	1	0	-2.795704	0.240860	-3.192927
44	1	0	-5.077353	-0.365156	-2.432674
45	1	0	-2.454538	0.902578	2.907637
46	1	0	-3.357171	-0.301098	3.870544
47	1	0	-2.020145	-0.835285	2.813202
48	1	0	2.133898	5.418965	-0.997641
49	1	0	1.750201	-3.782107	2.270156

----

7. Radical Cation (1,2)

Sum of electronic and thermal Enthalpies= -1207.561799

Frequencies -- 16.1796 18.4564 22.8047

Center Number	Atomic Number	Atomic Type	Coordinates (Angstroms) X	Y	Z
1	7	0	0.968613	0.163063	-2.060656
2	6	0	1.459642	1.543527	-1.987809
3	6	0	0.658033	2.419424	-1.044415
4	6	0	-0.243636	3.369343	-1.536892
5	6	0	0.838416	2.317156	0.339905
6	6	0	-0.948775	4.201647	-0.673677
7	6	0	0.139892	3.143723	1.212949
8	6	0	-0.757494	4.086887	0.705141
9	8	0	-1.470769	4.926046	1.516331

10	6	0	1.894744	-0.950238	-1.830378
11	6	0	2.471758	-0.975259	-0.429193
12	6	0	1.696235	-1.421651	0.649089
13	6	0	3.792058	-0.583942	-0.191540
14	6	0	2.224407	-1.476862	1.932543
15	6	0	4.333550	-0.634987	1.090399
16	6	0	3.545786	-1.078519	2.154789
17	8	0	4.018324	-1.149436	3.436638
18	6	0	-0.350755	-0.098658	-2.645804
19	6	0	-1.214661	-1.041069	-1.834179
20	6	0	-1.838862	-0.578654	-0.663882
21	6	0	-1.425381	-2.351506	-2.257399
22	6	0	-2.665307	-1.425570	0.063936
23	6	0	-2.254714	-3.204978	-1.527360
24	6	0	-2.872632	-2.750278	-0.372022
25	8	0	-3.686934	-3.589317	0.334214
26	8	0	-3.335101	-1.098753	1.208671
27	6	0	-3.232289	0.242629	1.707928
28	1	0	2.508134	1.507280	-1.698747
29	1	0	1.393344	1.937795	-3.009284
30	1	0	-0.387885	3.471820	-2.607084
31	1	0	1.535548	1.591339	0.742960
32	1	0	0.289695	3.064662	2.284951
33	1	0	-1.247438	4.766096	2.444136
34	1	0	1.365219	-1.873295	-2.058152
35	1	0	2.700835	-0.822005	-2.563748
36	1	0	0.670881	-1.733436	0.484172
37	1	0	4.411818	-0.245606	-1.015043
38	1	0	5.362569	-0.338672	1.267054
39	1	0	4.937996	-0.850896	3.475502
40	1	0	-0.841285	0.861317	-2.796022
41	1	0	-0.150135	-0.538960	-3.632039
42	1	0	-1.678370	0.440008	-0.335595
43	1	0	-0.954725	-2.711540	-3.165206
44	1	0	-2.432623	-4.223571	-1.853226
45	1	0	-4.048059	-3.111849	1.097297
46	1	0	-3.609360	0.958992	0.973147
47	1	0	-3.853524	0.271191	2.601617
48	1	0	-2.198323	0.481203	1.970243
49	1	0	-1.639621	4.943773	-1.057425
50	1	0	1.626380	-1.828130	2.765803

S1.1 - Compound 1

ArOH (0,1)

Sum of electronic and thermal Enthalpies= -1436.797915

Frequencies -- 5.7179 13.1450 15.3962

Center Number	Atomic Number	Atomic Type	Coordinates (Angstroms) X	Y	Z
1	7	0	0.070748	-0.231942	-1.154552
2	6	0	0.998717	-1.374148	-1.267721
3	6	0	2.141832	-1.338749	-0.274057

4	6	0	3.462353	-1.176091	-0.717093
5	6	0	1.905553	-1.491072	1.094161
6	6	0	4.520296	-1.159378	0.189610
7	6	0	2.960014	-1.474052	2.007829
8	6	0	4.264605	-1.306985	1.563095
9	8	0	5.844163	-1.003422	-0.130715
10	8	0	5.293353	-1.290117	2.472332
11	6	0	-1.171957	-0.490663	-1.908075
12	6	0	-2.059833	-1.549048	-1.288031
13	6	0	-2.666402	-1.313751	-0.042590
14	6	0	-2.313898	-2.753671	-1.941531
15	6	0	-3.498456	-2.267984	0.530950
16	6	0	-3.152213	-3.717563	-1.371687
17	6	0	-3.742087	-3.482098	-0.140247
18	8	0	-4.561359	-4.433804	0.414226
19	6	0	0.712327	1.014433	-1.615802
20	6	0	-0.034200	2.272989	-1.225946
21	6	0	-0.160969	2.621516	0.129574
22	6	0	-0.578833	3.118574	-2.190764
23	6	0	-0.823756	3.785159	0.501142
24	6	0	-1.244469	4.293130	-1.824159
25	6	0	-1.370627	4.628256	-0.485829
26	8	0	-2.025516	5.780789	-0.129700
27	8	0	-1.007046	4.226396	1.785615
28	6	0	-0.483986	3.439483	2.864005
29	6	0	6.203431	-0.830423	-1.507922
30	8	0	-4.142586	-2.146242	1.734320
31	6	0	-3.950709	-0.947675	2.497499
32	1	0	0.413770	-2.281021	-1.097254
33	1	0	1.405593	-1.446757	-2.290692
34	1	0	3.655629	-1.061585	-1.776568
35	1	0	0.892500	-1.628118	1.456150
36	1	0	2.780736	-1.595162	3.070785
37	1	0	6.129574	-1.162485	1.999627
38	1	0	-1.723510	0.451785	-1.951067
39	1	0	-0.945706	-0.773909	-2.950084
40	1	0	-2.483095	-0.377872	0.469974
41	1	0	-1.856601	-2.951100	-2.905276
42	1	0	-3.349521	-4.656216	-1.878096
43	1	0	-4.888141	-4.110671	1.267370
44	1	0	1.710400	1.046091	-1.171938
45	1	0	0.851905	1.001695	-2.710090
46	1	0	0.265764	1.975586	0.886400
47	1	0	-0.487900	2.866258	-3.241959
48	1	0	-1.669949	4.951629	-2.573666
49	1	0	-2.017574	5.858760	0.836310
50	1	0	0.603437	3.351474	2.790135
51	1	0	-0.746853	3.976231	3.774374
52	1	0	-0.939723	2.445695	2.877972
53	1	0	5.741132	0.070179	-1.921438
54	1	0	7.287063	-0.722937	-1.517914
55	1	0	5.915576	-1.704504	-2.098634
56	1	0	-4.305173	-0.073085	1.945016
57	1	0	-4.545058	-1.075871	3.400972

58	1	0	-2.897979	-0.820417	2.764356

----

1. ArO-/X (-1,1)

Sum of electronic and thermal Enthalpies= -1436.331646

Frequencies -- 12.8675 14.4297 19.1362

Center Number	Atomic Number	Atomic Type	Coordinates (Angstroms) X	Y	Z
1	7	0	0.067769	-0.232548	-1.133589
2	6	0	0.916835	-1.440327	-1.256682
3	6	0	2.062714	-1.505265	-0.271477
4	6	0	3.395351	-1.341281	-0.695838
5	6	0	1.840333	-1.747667	1.085239
6	6	0	4.456844	-1.408539	0.199899
7	6	0	2.900468	-1.814487	1.990523
8	6	0	4.252836	-1.646847	1.601185
9	8	0	5.782539	-1.251516	-0.166114
10	8	0	5.249629	-1.705078	2.443194
11	6	0	-1.184176	-0.393622	-1.893927
12	6	0	-2.147512	-1.399091	-1.296875
13	6	0	-2.654096	-1.206486	-0.000484
14	6	0	-2.577428	-2.505264	-2.027760
15	6	0	-3.563086	-2.105769	0.544579
16	6	0	-3.496563	-3.411104	-1.487818
17	6	0	-3.989688	-3.217165	-0.207848
18	8	0	-4.891274	-4.109833	0.317822
19	6	0	0.797720	0.969668	-1.575604
20	6	0	0.135109	2.274871	-1.186808
21	6	0	0.032107	2.633365	0.168194
22	6	0	-0.355878	3.152262	-2.152326
23	6	0	-0.554667	3.837666	0.538749
24	6	0	-0.945810	4.366843	-1.786952
25	6	0	-1.049003	4.711488	-0.449082
26	8	0	-1.631017	5.903786	-0.095690
27	8	0	-0.706932	4.291894	1.823017
28	6	0	-0.222877	3.479359	2.900909
29	6	0	6.081654	-1.013429	-1.540266
30	8	0	-4.120299	-2.020617	1.793977
31	6	0	-3.740742	-0.928714	2.642295
32	1	0	0.259550	-2.299761	-1.102267
33	1	0	1.309824	-1.525870	-2.285148
34	1	0	3.586221	-1.158997	-1.747309
35	1	0	0.825154	-1.890253	1.446230
36	1	0	2.705281	-2.007371	3.042401
37	1	0	-1.672393	0.584261	-1.924485
38	1	0	-0.976486	-0.674981	-2.940823
39	1	0	-2.330560	-0.348597	0.574774
40	1	0	-2.195745	-2.670571	-3.029734
41	1	0	-3.830728	-4.273082	-2.055089
42	1	0	-5.130843	-3.823401	1.212068
43	1	0	1.789027	0.927191	-1.118212
44	1	0	0.950975	0.955987	-2.668700

45	1	0	0.418819	1.962903	0.925242
46	1	0	-0.281909	2.893503	-3.203317
47	1	0	-1.330045	5.049703	-2.536982
48	1	0	-1.613485	5.986122	0.869653
49	1	0	0.856585	3.325884	2.818733
50	1	0	-0.445868	4.034505	3.810904
51	1	0	-0.737236	2.514796	2.923105
52	1	0	5.624243	-0.083492	-1.894689
53	1	0	7.166735	-0.925055	-1.599273
54	1	0	5.752517	-1.845457	-2.171993
55	1	0	-4.020342	0.028608	2.193832
56	1	0	-4.291111	-1.072722	3.570933
57	1	0	-2.666387	-0.947879	2.844679

----

2. ARO-/X’ (-1,1)

Sum of electronic and thermal Enthalpies= -1436.331014

Frequencies -- 14.1292 14.6398 18.6175

Center Number	Atomic Number	Atomic Type	Coordinates (Angstroms) X	Y	Z
1	7	0	0.173041	-0.074295	-0.696633
2	6	0	0.374655	1.384890	-0.754030
3	6	0	-0.673981	2.184579	-0.007587
4	6	0	-1.512011	3.070777	-0.699745
5	6	0	-0.808749	2.077338	1.378642
6	6	0	-2.464863	3.827328	-0.020912
7	6	0	-1.764704	2.828204	2.064172
8	6	0	-2.594198	3.700186	1.371887
9	8	0	-3.331225	4.721215	-0.596150
10	8	0	-3.532122	4.433264	2.057140
11	6	0	1.367030	-0.790472	-1.203814
12	6	0	2.599439	-0.670181	-0.335454
13	6	0	3.694786	0.116155	-0.741852
14	6	0	2.707188	-1.351514	0.878140
15	6	0	4.842925	0.219127	0.034407
16	6	0	3.857710	-1.253093	1.663553
17	6	0	4.978105	-0.471300	1.286995
18	8	0	6.063595	-0.378235	2.007554
19	6	0	-1.026284	-0.466441	-1.460417
20	6	0	-1.496855	-1.880789	-1.190749
21	6	0	-1.951651	-2.236673	0.090202
22	6	0	-1.520783	-2.840129	-2.201581
23	6	0	-2.410387	-3.523454	0.346286
24	6	0	-1.981798	-4.136935	-1.951453
25	6	0	-2.423311	-4.482902	-0.684603
26	8	0	-2.873705	-5.757741	-0.444704
27	8	0	-2.879585	-3.978889	1.550859
28	6	0	-2.908503	-3.073688	2.662559
29	6	0	-3.276973	4.913357	-2.015707
30	8	0	5.943811	0.978324	-0.321701
31	6	0	5.899536	1.700158	-1.550977
32	1	0	1.353151	1.588876	-0.313236

33	1	0	0.419075	1.733641	-1.800663
34	1	0	-1.413987	3.163545	-1.774273
35	1	0	-0.163645	1.406128	1.934666
36	1	0	-1.870846	2.748310	3.140775
37	1	0	-4.021045	4.983548	1.426905
38	1	0	1.086179	-1.843332	-1.285840
39	1	0	1.605559	-0.448096	-2.226524
40	1	0	3.631243	0.647020	-1.684869
41	1	0	1.885111	-1.975738	1.218697
42	1	0	3.922241	-1.797654	2.602195
43	1	0	-1.825936	0.226538	-1.186818
44	1	0	-0.857806	-0.343978	-2.544360
45	1	0	-1.944959	-1.497953	0.881498
46	1	0	-1.175207	-2.582937	-3.197267
47	1	0	-1.997243	-4.885527	-2.736254
48	1	0	-3.123312	-5.832297	0.488731
49	1	0	-3.560734	-2.221159	2.453717
50	1	0	-3.309863	-3.646035	3.497604
51	1	0	-1.901679	-2.723754	2.906252
52	1	0	-3.500044	3.982341	-2.544209
53	1	0	-4.042210	5.654544	-2.241604
54	1	0	-2.297637	5.291940	-2.321247
55	1	0	5.085855	2.433296	-1.555968
56	1	0	6.853975	2.221789	-1.625967
57	1	0	5.788384	1.026338	-2.407276

----

3. ARO-/Y (-1,1)
Sum of electronic and thermal Enthalpies= -1436.331082
Frequencies -- 13.9669 18.9189 22.3496

Center Number	Atomic Number	Atomic Type	Coordinates (Angstroms) X	Y	Z
1	7	0	-0.112158	0.072624	-0.674110
2	6	0	-0.170904	-1.396416	-0.789524
3	6	0	0.929596	-2.123830	-0.043667
4	6	0	1.869627	-2.892202	-0.745303
5	6	0	1.010488	-2.071579	1.349722
6	6	0	2.868340	-3.588799	-0.068428
7	6	0	2.011054	-2.763324	2.033958
8	6	0	2.940087	-3.520346	1.332856
9	8	0	3.833439	-4.367695	-0.653241
10	8	0	3.917896	-4.199118	2.017906
11	6	0	-1.357594	0.681739	-1.177770
12	6	0	-2.572359	0.417342	-0.311898
13	6	0	-3.634982	-0.356559	-0.800590
14	6	0	-2.670230	0.957055	0.972802
15	6	0	-4.765534	-0.588317	-0.020029
16	6	0	-3.798421	0.726402	1.761238
17	6	0	-4.845160	-0.043744	1.272384
18	8	0	-5.950917	-0.262248	2.057485
19	6	0	1.062284	0.606539	-1.402230
20	6	0	1.355915	2.066826	-1.145918

21	6	0	1.809299	2.496604	0.118749
22	6	0	1.215690	3.028636	-2.144312
23	6	0	2.097376	3.831041	0.367894
24	6	0	1.508139	4.374896	-1.902656
25	6	0	1.955617	4.844316	-0.645381
26	8	0	2.225932	6.097964	-0.402305
27	8	0	2.543611	4.306173	1.587504
28	6	0	2.718153	3.374434	2.653921
29	6	0	3.841874	-4.494152	-2.081226
30	8	0	-5.857078	-1.327416	-0.397644
31	6	0	-5.866372	-1.921071	-1.702213
32	1	0	-1.135230	-1.714528	-0.385629
33	1	0	-0.154400	-1.704911	-1.849437
34	1	0	1.814632	-2.939734	-1.825807
35	1	0	0.288223	-1.490387	1.912358
36	1	0	2.075645	-2.726421	3.116171
37	1	0	4.481583	-4.664021	1.381579
38	1	0	-1.187233	1.759552	-1.227462
39	1	0	-1.566776	0.345453	-2.208290
40	1	0	-3.570772	-0.776149	-1.796954
41	1	0	-1.864427	1.567038	1.366128
42	1	0	-3.878654	1.146477	2.758117
43	1	0	-6.576447	-0.820405	1.571512
44	1	0	1.920208	0.002478	-1.095024
45	1	0	0.939311	0.449990	-2.488615
46	1	0	1.932020	1.761227	0.905392
47	1	0	0.872656	2.729186	-3.131436
48	1	0	1.390727	5.107134	-2.697362
49	1	0	3.468407	2.616613	2.404403
50	1	0	3.066324	3.957937	3.506509
51	1	0	1.775050	2.882098	2.913550
52	1	0	3.989447	-3.521323	-2.558298
53	1	0	4.682203	-5.146642	-2.313836
54	1	0	2.913818	-4.949429	-2.438136
55	1	0	-5.040776	-2.629357	-1.814527
56	1	0	-6.815217	-2.450155	-1.778151
57	1	0	-5.810156	-1.154305	-2.479902

----

4. ARO./X (0,2)

Sum of electronic and thermal Enthalpies= -1436.175360

Frequencies -- 12.9129 15.1788 18.8073

Center Number	Atomic Number	Atomic Type	Coordinates (Angstroms) X	Y	Z
1	7	0	-0.128431	-0.124257	1.150899
2	6	0	-1.243382	-1.071908	1.286846
3	6	0	-2.343773	-0.855046	0.277400
4	6	0	-3.667840	-0.709369	0.697555
5	6	0	-2.037679	-0.847565	-1.114408
6	6	0	-4.691510	-0.552979	-0.231677
7	6	0	-3.020410	-0.696446	-2.051321
8	6	0	-4.403344	-0.539194	-1.672957

9	8	0	-5.985925	-0.399941	0.067263
10	8	0	-5.325677	-0.400267	-2.517488
11	6	0	1.060895	-0.601571	1.884618
12	6	0	1.707804	-1.827213	1.276365
13	6	0	2.291817	-1.754742	0.000154
14	6	0	1.761855	-3.033251	1.973238
15	6	0	2.906266	-2.868568	-0.559815
16	6	0	2.382077	-4.156903	1.417385
17	6	0	2.951571	-4.080599	0.156784
18	8	0	3.558405	-5.185890	-0.384040
19	6	0	-0.532959	1.220113	1.613981
20	6	0	0.436376	2.319483	1.236271
21	6	0	0.625676	2.656872	-0.114516
22	6	0	1.135421	3.029619	2.210886
23	6	0	1.501867	3.674574	-0.472969
24	6	0	2.016977	4.056180	1.857760
25	6	0	2.205554	4.379035	0.523539
26	8	0	3.075267	5.384736	0.184129
27	8	0	1.763359	4.089035	-1.752292
28	6	0	1.082533	3.443014	-2.836285
29	6	0	-6.399805	-0.394097	1.447188
30	8	0	3.506703	-2.916494	-1.790283
31	6	0	3.522519	-1.729025	-2.593916
32	1	0	-0.842360	-2.078881	1.128479
33	1	0	-1.666233	-1.055196	2.304366
34	1	0	-3.886706	-0.715138	1.757802
35	1	0	-1.006316	-0.971936	-1.422633
36	1	0	-2.797999	-0.698155	-3.112865
37	1	0	1.783671	0.217841	1.887730
38	1	0	0.811446	-0.810453	2.938287
39	1	0	2.262541	-0.820584	-0.546211
40	1	0	1.318621	-3.106018	2.960667
41	1	0	2.424815	-5.096254	1.957833
42	1	0	3.899053	-4.959360	-1.262533
43	1	0	-1.506372	1.435837	1.164976
44	1	0	-0.678508	1.226631	2.707056
45	1	0	0.079598	2.119372	-0.879292
46	1	0	0.998784	2.784363	3.258775
47	1	0	2.564014	4.607870	2.614564
48	1	0	3.094827	5.473273	-0.780736
49	1	0	0.000494	3.575352	-2.750214
50	1	0	1.440448	3.931762	-3.741215
51	1	0	1.327993	2.378129	-2.871650
52	1	0	-5.932212	0.435166	1.982452
53	1	0	-7.479209	-0.259781	1.422814
54	1	0	-6.151748	-1.344729	1.924505
55	1	0	4.063669	-0.924352	-2.088681
56	1	0	4.043374	-2.001209	-3.510744
57	1	0	2.505225	-1.405660	-2.830775

----

5. ARO./X’ (0,2)
   Sum of electronic and thermal Enthalpies= -1436.174753
   Frequencies -- 16.4305 17.5647 18.5957

Center Number	Atomic Number	Atomic Type	Coordinates (Angstroms) X	Y	Z
1	7	0	0.020226	-0.228384	-1.125481
2	6	0	1.008071	-1.320259	-1.262844
3	6	0	2.174304	-1.221143	-0.302058
4	6	0	3.471283	-0.996073	-0.785931
5	6	0	1.986223	-1.375017	1.073297
6	6	0	4.553213	-0.919638	0.088278
7	6	0	3.064930	-1.296687	1.954836
8	6	0	4.345612	-1.067514	1.470155
9	8	0	5.857298	-0.702730	-0.272729
10	8	0	5.398066	-0.991444	2.347792
11	6	0	-1.201073	-0.551204	-1.876012
12	6	0	-2.035922	-1.644453	-1.252563
13	6	0	-2.390820	-1.580307	0.101246
14	6	0	-2.507155	-2.717994	-2.056288
15	6	0	-3.197340	-2.558254	0.663899
16	6	0	-3.302975	-3.699397	-1.530591
17	6	0	-3.694520	-3.683330	-0.143589
18	8	0	-4.421891	-4.574813	0.364815
19	6	0	0.586515	1.058759	-1.576657
20	6	0	-0.244247	2.263310	-1.189593
21	6	0	-0.389196	2.609159	0.164775
22	6	0	-0.853152	3.061671	-2.156476
23	6	0	-1.131723	3.724373	0.533598
24	6	0	-1.599216	4.187537	-1.792579
25	6	0	-1.741946	4.520917	-0.455383
26	8	0	-2.473977	5.626653	-0.102901
27	8	0	-1.339716	4.157946	1.816426
28	6	0	-0.747437	3.422255	2.895233
29	6	0	6.165981	-0.524493	-1.661536
30	8	0	-3.600631	-2.585995	1.940295
31	6	0	-3.186900	-1.534333	2.832887
32	1	0	0.477017	-2.258064	-1.077771
33	1	0	1.389237	-1.369946	-2.296402
34	1	0	3.626461	-0.879861	-1.851392
35	1	0	0.993057	-1.561003	1.466796
36	1	0	2.923459	-1.417555	3.023459
37	1	0	6.210449	-0.812117	1.850818
38	1	0	-1.815607	0.354970	-1.914960
39	1	0	-0.971978	-0.820497	-2.919983
40	1	0	-2.030292	-0.754273	0.698824
41	1	0	-2.220764	-2.752089	-3.101979
42	1	0	-3.661645	-4.524955	-2.135478
43	1	0	1.575441	1.149892	-1.121468
44	1	0	0.736884	1.058052	-2.669132
45	1	0	0.087132	2.000507	0.922883
46	1	0	-0.748409	2.811032	-3.206761
47	1	0	-2.073721	4.810007	-2.543400
48	1	0	-2.463582	5.715538	0.862210
49	1	0	0.342667	3.416542	2.810179
50	1	0	-1.041038	3.945453	3.804063

51	1	0	-1.126480	2.397029	2.922016
52	1	0	5.648234	0.349400	-2.066671
53	1	0	7.242323	-0.364954	-1.705381
54	1	0	5.902940	-1.416380	-2.236898
55	1	0	-3.555809	-0.568169	2.481475
56	1	0	-3.637179	-1.777785	3.793089
57	1	0	-2.098631	-1.516335	2.924021

----

6. ARO./Y (0,2)

Sum of electronic and thermal Enthalpies= -1436.174553

Frequencies -- 13.9687 16.4574 17.4446

Center Number	Atomic Number	Atomic Type	Coordinates (Angstroms) X	Y	Z
1	7	0	0.083532	-0.124765	-1.099930
2	6	0	0.977859	-1.293894	-1.217033
3	6	0	2.118971	-1.295002	-0.221243
4	6	0	3.445422	-1.197212	-0.665875
5	6	0	1.875895	-1.422315	1.148440
6	6	0	4.503314	-1.224017	0.240372
7	6	0	2.930588	-1.447318	2.061562
8	6	0	4.241685	-1.348825	1.615089
9	8	0	5.832380	-1.136113	-0.082238
10	8	0	5.270625	-1.378785	2.523158
11	6	0	-1.153766	-0.324461	-1.884258
12	6	0	-2.064982	-1.404804	-1.342461
13	6	0	-2.659111	-1.259720	-0.077322
14	6	0	-2.355230	-2.541882	-2.094782
15	6	0	-3.514705	-2.236820	0.418026
16	6	0	-3.219829	-3.525961	-1.605315
17	6	0	-3.798721	-3.379705	-0.354981
18	8	0	-4.646146	-4.348838	0.119587
19	6	0	0.767404	1.099697	-1.536502
20	6	0	0.072065	2.376375	-1.127496
21	6	0	-0.343392	2.565773	0.196830
22	6	0	-0.113133	3.414060	-2.081340
23	6	0	-0.937786	3.757597	0.583678
24	6	0	-0.696313	4.601448	-1.730999
25	6	0	-1.144737	4.846915	-0.383268
26	8	0	-1.683189	5.929232	-0.035443
27	8	0	-1.363659	4.042011	1.820745
28	6	0	-1.199000	3.064688	2.865666
29	6	0	6.198153	-1.021467	-1.463816
30	8	0	-4.147480	-2.204056	1.632662
31	6	0	-3.935043	-1.073414	2.488262
32	1	0	0.364137	-2.182440	-1.052295
33	1	0	1.384339	-1.373365	-2.239187
34	1	0	3.643776	-1.100754	-1.726200
35	1	0	0.857900	-1.509601	1.511771
36	1	0	2.746441	-1.551434	3.125458
37	1	0	6.112778	-1.319567	2.047373
38	1	0	-1.691607	0.628045	-1.882380

39	1	0	-0.913578	-0.551206	-2.936344
40	1	0	-2.447929	-0.377024	0.512538
41	1	0	-1.906440	-2.669297	-3.074134
42	1	0	-3.447268	-4.410978	-2.189578
43	1	0	-4.968788	-4.084692	0.994409
44	1	0	1.763468	1.105140	-1.080141
45	1	0	0.922583	1.105988	-2.627804
46	1	0	-0.188946	1.771043	0.913543
47	1	0	0.213270	3.249008	-3.102654
48	1	0	-0.845884	5.396030	-2.453733
49	1	0	-0.140154	2.843716	3.017751
50	1	0	-1.615666	3.525384	3.759017
51	1	0	-1.747762	2.151401	2.625514
52	1	0	5.784390	-0.109303	-1.902514
53	1	0	7.285989	-0.972697	-1.476967
54	1	0	5.862481	-1.895104	-2.029371
55	1	0	-4.280655	-0.152870	2.009826
56	1	0	-4.526192	-1.265982	3.382341
57	1	0	-2.879315	-0.982103	2.758087

----

7. Radical Cation (1,2)
Sum of electronic and thermal Enthalpies= -1436.605877
Frequencies -- -2.4227 14.1753 19.4915
1 negative frequency found – ignored since value is too small, likely due to numerical noise.

Center Number	Atomic Number	Atomic Type	Coordinates (Angstroms) X	Y	Z
1	7	0	-0.278772	-0.118647	-1.956316
2	6	0	-0.154501	-1.567273	-1.778045
3	6	0	0.780806	-1.953218	-0.650330
4	6	0	2.031830	-2.516254	-0.938192
5	6	0	0.392612	-1.772820	0.678425
6	6	0	2.881535	-2.897028	0.096101
7	6	0	1.240560	-2.154605	1.716943
8	6	0	2.481744	-2.709322	1.434933
9	8	0	3.304346	-3.071974	2.462510
10	6	0	-1.608362	0.478039	-2.094282
11	6	0	-2.460594	0.317544	-0.851155
12	6	0	-2.182252	1.067266	0.293255
13	6	0	-3.546483	-0.568533	-0.854619
14	6	0	-2.982123	0.942122	1.428020
15	6	0	-4.346498	-0.695765	0.276809
16	6	0	-4.057324	0.063799	1.428441
17	8	0	-4.832049	-0.051792	2.546218
18	6	0	0.906106	0.668332	-2.317040
19	6	0	1.088718	1.935103	-1.505802
20	6	0	1.579737	1.855725	-0.192549
21	6	0	0.810845	3.181208	-2.063244
22	6	0	1.787067	3.014932	0.544709
23	6	0	1.017466	4.348301	-1.324631
24	6	0	1.500718	4.271527	-0.027119
25	8	0	1.701794	5.418008	0.688483

26	8	0	2.264718	3.074177	1.823350
27	6	0	2.637977	1.853212	2.478081
28	1	0	-1.153402	-1.970317	-1.618437
29	1	0	0.222884	-1.959271	-2.731278
30	1	0	2.328075	-2.663787	-1.968870
31	1	0	-0.572619	-1.337854	0.909556
32	1	0	0.947542	-2.024918	2.752719
33	1	0	4.128431	-3.433497	2.101731
34	1	0	-1.477811	1.527894	-2.351306
35	1	0	-2.084111	-0.026918	-2.944167
36	1	0	-1.345341	1.755178	0.302676
37	1	0	-3.767874	-1.142336	-1.745009
38	1	0	-5.528545	-0.707450	2.385094
39	1	0	1.772975	0.015629	-2.231514
40	1	0	0.774816	0.927135	-3.376304
41	1	0	1.798452	0.888666	0.241221
42	1	0	0.442950	3.252283	-3.080613
43	1	0	0.811530	5.322934	-1.752490
44	1	0	2.057309	5.189483	1.561442
45	1	0	3.436935	1.348323	1.928833
46	1	0	2.997591	2.147058	3.462934
47	1	0	1.776013	1.189557	2.583216
48	1	0	-2.779013	1.522847	2.320554
49	8	0	-5.435007	-1.509167	0.394690
50	8	0	4.114021	-3.462895	-0.053259
51	6	0	-5.800649	-2.339574	-0.717816
52	1	0	-4.993066	-3.033945	-0.964445
53	1	0	-6.677994	-2.895810	-0.392431
54	1	0	-6.053000	-1.730281	-1.589305
55	6	0	4.613840	-3.693347	-1.378931
56	1	0	4.704545	-2.752458	-1.928233
57	1	0	5.598763	-4.138047	-1.247141
58	1	0	3.966144	-4.386678	-1.921926

S1.2- Compound 2

ArOH (0,1)

Sum of electronic and thermal Enthalpies= -1665.835855

Frequencies -- 12.0448 12.8955 15.9333

Center Number	Atomic Number	Atomic Type	Coordinates (Angstroms) X	Y	Z
1	7	0	-0.015894	0.118745	-1.175795
2	6	0	0.294519	-1.314705	-1.337053
3	6	0	1.520078	-1.772184	-0.572050
4	6	0	2.631128	-2.268180	-1.258190
5	6	0	1.531579	-1.726622	0.826699
6	6	0	3.757450	-2.715427	-0.559114
7	6	0	2.654048	-2.169129	1.523692
8	6	0	3.771444	-2.663767	0.837511
9	8	0	4.891709	-3.215368	-1.140704
10	8	0	4.883335	-3.103564	1.511192
11	6	0	-1.365582	0.420039	-1.689658

12	6	0	-2.490896	-0.165594	-0.860339
13	6	0	-2.680531	0.253014	0.461787
14	6	0	-3.362999	-1.100639	-1.423096
15	6	0	-3.730747	-0.263664	1.222447
16	6	0	-4.416198	-1.614689	-0.663711
17	6	0	-4.603689	-1.203152	0.658702
18	8	0	-5.632717	-1.698654	1.419769
19	6	0	0.997111	0.950805	-1.854548
20	6	0	0.937785	2.419443	-1.490762
21	6	0	1.233471	2.831605	-0.180229
22	6	0	0.623317	3.386631	-2.443964
23	6	0	1.206698	4.178970	0.160342
24	6	0	0.597724	4.744350	-2.109214
25	6	0	0.886428	5.143842	-0.814379
26	8	0	0.861315	6.477671	-0.491982
27	8	0	1.478441	4.695408	1.400225
28	6	0	1.851949	3.794686	2.451287
29	6	0	4.945216	-3.291383	-2.571022
30	8	0	-4.003704	0.079889	2.518939
31	6	0	-3.166878	1.054808	3.153585
32	1	0	-0.573164	-1.873805	-0.978233
33	1	0	0.418312	-1.569629	-2.403231
34	1	0	2.614523	-2.302644	-2.339451
35	1	0	0.668435	-1.352251	1.360721
36	1	0	4.722914	-3.009537	2.462362
37	1	0	-1.468815	1.507889	-1.704206
38	1	0	-1.471008	0.077406	-2.733041
39	1	0	-2.008438	0.984353	0.890050
40	1	0	-3.217301	-1.423690	-2.445644
41	1	0	-6.141134	-2.319201	0.875812
42	1	0	1.976742	0.556865	-1.572901
43	1	0	0.915905	0.847506	-2.950062
44	1	0	1.487910	2.089886	0.566177
45	1	0	0.395256	3.086811	-3.461277
46	1	0	0.353963	5.498155	-2.849963
47	1	0	1.101611	6.579586	0.441242
48	1	0	2.767093	3.254367	2.193839
49	1	0	2.028912	4.419508	3.325487
50	1	0	1.045945	3.086728	2.662522
51	1	0	4.857073	-2.298759	-3.021743
52	1	0	5.922662	-3.711593	-2.804500
53	1	0	4.162723	-3.949095	-2.960011
54	1	0	-3.210771	2.012723	2.627726
55	1	0	-3.567197	1.172908	4.159704
56	1	0	-2.131421	0.707791	3.210886
57	8	0	2.790937	-2.179749	2.887662
58	6	0	1.697758	-1.716361	3.691492
59	1	0	0.806867	-2.328697	3.527963
60	1	0	2.028346	-1.819214	4.724029
61	1	0	1.475196	-0.667109	3.479828
62	8	0	-5.336219	-2.533368	-1.099192
63	6	0	-5.249565	-3.003495	-2.451088
64	1	0	-4.302657	-3.522113	-2.624192
65	1	0	-6.076097	-3.702195	-2.572334

66	1	0	-5.359613	-2.177504	-3.159020

----

1. ArO-/X (-1,1)

Sum of electronic and thermal Enthalpies= -1665.370367

Frequencies -- 12.9174 13.4091 16.2326

Center Number	Atomic Number	Atomic Type	Coordinates (Angstroms) X	Y	Z
1	7	0	-0.042009	0.119139	-1.146482
2	6	0	0.249213	-1.318950	-1.288314
3	6	0	1.472714	-1.781427	-0.522922
4	6	0	2.574037	-2.303037	-1.205989
5	6	0	1.491979	-1.714052	0.874905
6	6	0	3.697929	-2.753830	-0.505096
7	6	0	2.611855	-2.160178	1.573536
8	6	0	3.719444	-2.680072	0.890274
9	8	0	4.823327	-3.277562	-1.083891
10	8	0	4.828832	-3.123470	1.566780
11	6	0	-1.395826	0.434224	-1.658278
12	6	0	-2.531766	-0.150802	-0.848967
13	6	0	-2.815788	0.327241	0.437788
14	6	0	-3.338234	-1.164961	-1.377667
15	6	0	-3.872769	-0.202901	1.175174
16	6	0	-4.397911	-1.692070	-0.638637
17	6	0	-4.718930	-1.239440	0.673990
18	8	0	-5.712451	-1.732701	1.363362
19	6	0	0.976825	0.925125	-1.845598
20	6	0	0.945466	2.400566	-1.504827
21	6	0	1.245832	2.827627	-0.200171
22	6	0	0.655443	3.359105	-2.474422
23	6	0	1.246735	4.180608	0.118648
24	6	0	0.657208	4.722302	-2.161463
25	6	0	0.949405	5.136314	-0.872016
26	8	0	0.950377	6.475650	-0.570390
27	8	0	1.526509	4.711001	1.351096
28	6	0	1.869537	3.818676	2.419538
29	6	0	4.865608	-3.385158	-2.512304
30	8	0	-4.205061	0.223872	2.450249
31	6	0	-3.428716	1.266283	3.037953
32	1	0	-0.624308	-1.859640	-0.916569
33	1	0	0.364441	-1.592627	-2.351360
34	1	0	2.551620	-2.354652	-2.286502
35	1	0	0.636177	-1.320128	1.406630
36	1	0	4.671365	-3.013993	2.516834
37	1	0	-1.479518	1.524068	-1.667229
38	1	0	-1.491516	0.102271	-2.707103
39	1	0	-2.203635	1.118302	0.853451
40	1	0	-3.128467	-1.537991	-2.372893
41	1	0	1.954030	0.521605	-1.568149
42	1	0	0.884281	0.807544	-2.939232
43	1	0	1.480735	2.092593	0.559121
44	1	0	0.423407	3.047984	-3.487453

45	1	0	0.430760	5.468937	-2.914909
46	1	0	1.177851	6.585910	0.365053
47	1	0	2.773446	3.252445	2.178504
48	1	0	2.054630	4.453158	3.285073
49	1	0	1.045065	3.133774	2.635275
50	1	0	4.788030	-2.401536	-2.984268
51	1	0	5.835425	-3.824001	-2.743447
52	1	0	4.071280	-4.040044	-2.881624
53	1	0	-3.487649	2.189330	2.451604
54	1	0	-3.859819	1.439388	4.024355
55	1	0	-2.379966	0.971585	3.149338
56	8	0	2.755250	-2.151205	2.936986
57	6	0	1.669849	-1.664817	3.738147
58	1	0	0.771692	-2.269240	3.585695
59	1	0	2.002848	-1.757360	4.770897
60	1	0	1.458135	-0.616159	3.512938
61	8	0	-5.229693	-2.696834	-1.105862
62	6	0	-4.983633	-3.227657	-2.406513
63	1	0	-3.989308	-3.681961	-2.473007
64	1	0	-5.740579	-3.996714	-2.562821
65	1	0	-5.086135	-2.459145	-3.180075

----

2. ARO-/X' (-1,1)

Sum of electronic and thermal Enthalpies= -1665.370330

Frequencies -- 14.0196 16.3835 17.8828

Center Number	Atomic Number	Atomic Type	Coordinates (Angstroms) X	Y	Z
1	7	0	0.022478	0.115459	-1.164727
2	6	0	0.305823	-1.328034	-1.339827
3	6	0	1.511560	-1.831677	-0.578151
4	6	0	2.674266	-2.225378	-1.250275
5	6	0	1.479695	-1.934061	0.819510
6	6	0	3.780065	-2.701552	-0.544325
7	6	0	2.585056	-2.408510	1.522186
8	6	0	3.793188	-2.814640	0.876351
9	8	0	4.958361	-3.105465	-1.152161
10	8	0	4.826763	-3.260242	1.538873
11	6	0	-1.308834	0.452128	-1.700257
12	6	0	-2.464216	-0.103787	-0.891169
13	6	0	-2.649785	0.299590	0.436193
14	6	0	-3.369226	-0.991557	-1.477980
15	6	0	-3.728351	-0.185048	1.177858
16	6	0	-4.450568	-1.473910	-0.737374
17	6	0	-4.634716	-1.076727	0.589821
18	8	0	-5.691196	-1.541317	1.333277
19	6	0	1.064449	0.935551	-1.810222
20	6	0	1.031423	2.398588	-1.421157
21	6	0	1.320003	2.780292	-0.099813
22	6	0	0.746901	3.389809	-2.359105
23	6	0	1.315182	4.121283	0.266063
24	6	0	0.742891	4.741352	-1.998658

25	6	0	1.023672	5.110320	-0.693142
26	8	0	1.018870	6.438310	-0.344400
27	8	0	1.582776	4.609575	1.518425
28	6	0	1.929081	3.682893	2.556527
29	6	0	5.045206	-3.030628	-2.573757
30	8	0	-3.998936	0.147343	2.478000
31	6	0	-3.116464	1.059566	3.143371
32	1	0	-0.585274	-1.863350	-1.001992
33	1	0	0.428112	-1.563806	-2.411633
34	1	0	2.705805	-2.155542	-2.330875
35	1	0	0.581159	-1.641678	1.349088
36	1	0	-1.385714	1.542440	-1.715333
37	1	0	-1.406984	0.114203	-2.746378
38	1	0	-1.950758	0.992810	0.884078
39	1	0	-3.226300	-1.304274	-2.504143
40	1	0	-6.217598	-2.134065	0.775836
41	1	0	2.028712	0.513580	-1.517422
42	1	0	1.001828	0.854216	-2.909347
43	1	0	1.551835	2.019282	0.634556
44	1	0	0.525424	3.113666	-3.384596
45	1	0	0.522057	5.513718	-2.727442
46	1	0	1.248670	6.516758	0.593772
47	1	0	2.837386	3.131383	2.298936
48	1	0	2.107158	4.288318	3.444073
49	1	0	1.108853	2.985213	2.745894
50	1	0	4.931514	-2.000969	-2.929216
51	1	0	6.041580	-3.390860	-2.831285
52	1	0	4.296214	-3.667828	-3.055868
53	1	0	-3.105083	2.031823	2.642661
54	1	0	-3.517886	1.172902	4.149617
55	1	0	-2.101406	0.656522	3.198065
56	8	0	2.616960	-2.537245	2.900616
57	6	0	1.452604	-2.175229	3.641111
58	1	0	0.591124	-2.789732	3.359086
59	1	0	1.696465	-2.357543	4.688041
60	1	0	1.204923	-1.116951	3.507528
61	8	0	-5.402642	-2.347544	-1.197042
62	6	0	-5.315478	-2.806533	-2.552605
63	1	0	-4.386669	-3.358899	-2.719056
64	1	0	-6.166261	-3.471869	-2.692450
65	1	0	-5.384440	-1.969590	-3.252847

----

3. ARO-/Y (-1,1)

Sum of electronic and thermal Enthalpies= -1665.370126

Frequencies -- 11.1034 14.9466 15.3738

Center Number	Atomic Number	Atomic Type	Coordinates (Angstroms) X	Y	Z
1	7	0	-0.002734	0.160169	-1.119049
2	6	0	0.388268	-1.247910	-1.313790
3	6	0	1.644697	-1.656217	-0.570720
4	6	0	2.762947	-2.102951	-1.278683

5	6	0	1.678054	-1.621380	0.827901
6	6	0	3.916240	-2.513637	-0.601906
7	6	0	2.828070	-2.026299	1.502704
8	6	0	3.951429	-2.474150	0.794663
9	8	0	5.058185	-2.966149	-1.207360
10	8	0	5.090087	-2.879005	1.446192
11	6	0	-1.363550	0.391757	-1.635256
12	6	0	-2.457429	-0.276864	-0.826754
13	6	0	-2.663360	0.083611	0.509971
14	6	0	-3.286948	-1.230265	-1.422342
15	6	0	-3.686591	-0.508608	1.251958
16	6	0	-4.314167	-1.819161	-0.681880
17	6	0	-4.516729	-1.466199	0.655064
18	8	0	-5.519829	-2.036914	1.398964
19	6	0	0.968874	1.065694	-1.775078
20	6	0	0.791873	2.529990	-1.445580
21	6	0	1.051266	3.010716	-0.145108
22	6	0	0.395545	3.454081	-2.410304
23	6	0	0.910084	4.355016	0.169315
24	6	0	0.255485	4.811156	-2.102469
25	6	0	0.499897	5.328632	-0.808879
26	8	0	0.372213	6.591996	-0.505154
27	8	0	1.149942	4.880540	1.425723
28	6	0	1.577379	3.995107	2.459457
29	6	0	5.094155	-3.012223	-2.639481
30	8	0	-3.971308	-0.225609	2.560696
31	6	0	-3.181560	0.767342	3.227439
32	1	0	-0.442439	-1.866054	-0.963173
33	1	0	0.518258	-1.471213	-2.386893
34	1	0	2.731286	-2.127444	-2.359890
35	1	0	0.810846	-1.283169	1.379030
36	1	0	4.942792	-2.799113	2.400751
37	1	0	-1.528767	1.471841	-1.627405
38	1	0	-1.444192	0.066065	-2.686840
39	1	0	-2.025734	0.829981	0.964002
40	1	0	-3.128946	-1.508796	-2.456124
41	1	0	-6.000850	-2.659781	0.833336
42	1	0	1.962640	0.736697	-1.459119
43	1	0	0.928244	0.937094	-2.871255
44	1	0	1.369608	2.308051	0.616197
45	1	0	0.191841	3.116118	-3.423212
46	1	0	-0.053821	5.513597	-2.872193
47	1	0	2.531324	3.518973	2.209106
48	1	0	1.705890	4.613244	3.348290
49	1	0	0.826866	3.223577	2.661107
50	1	0	4.961433	-2.015134	-3.068804
51	1	0	6.083623	-3.389599	-2.894585
52	1	0	4.332153	-3.691901	-3.031361
53	1	0	-3.273401	1.738982	2.734119
54	1	0	-3.584877	0.831668	4.237243
55	1	0	-2.130229	0.469821	3.272258
56	8	0	2.987810	-2.041027	2.864330
57	6	0	1.893517	-1.618468	3.688915
58	1	0	1.021371	-2.260372	3.537954

59	1	0	2.245256	-1.714083	4.715148
60	1	0	1.631587	-0.576704	3.485774
61	8	0	-5.193007	-2.762121	-1.150991
62	6	0	-5.089409	-3.176381	-2.519743
63	1	0	-4.121792	-3.647595	-2.712928
64	1	0	-5.885911	-3.904353	-2.667096
65	1	0	-5.235882	-2.329619	-3.195787

----

4. ARO./X (0,2)

Sum of electronic and thermal Enthalpies= -1665.219564

Frequencies -- 11.3908 13.6078 15.8987

Center Number	Atomic Number	Atomic Type	Coordinates (Angstroms) X	Y	Z
1	7	0	-0.063270	0.101681	-1.231760
2	6	0	0.363674	-1.298658	-1.436374
3	6	0	1.616479	-1.679193	-0.674711
4	6	0	2.790720	-1.995685	-1.362130
5	6	0	1.592081	-1.740333	0.723289
6	6	0	3.944371	-2.367586	-0.663428
7	6	0	2.740440	-2.110316	1.419857
8	6	0	3.920815	-2.424350	0.732988
9	8	0	5.141015	-2.692162	-1.243552
10	8	0	5.058813	-2.793041	1.404691
11	6	0	-1.434517	0.295784	-1.725466
12	6	0	-2.489172	-0.349362	-0.855619
13	6	0	-2.552469	-0.020580	0.514253
14	6	0	-3.419186	-1.231867	-1.430125
15	6	0	-3.532059	-0.567257	1.315181
16	6	0	-4.413989	-1.798386	-0.653553
17	6	0	-4.523033	-1.498618	0.773836
18	8	0	-5.416035	-2.008974	1.492045
19	6	0	0.869995	1.041082	-1.886522
20	6	0	0.690594	2.481585	-1.458053
21	6	0	1.001573	2.864987	-0.142276
22	6	0	0.242410	3.451991	-2.352741
23	6	0	0.858340	4.187058	0.261255
24	6	0	0.096910	4.784755	-1.953718
25	6	0	0.400038	5.155279	-0.653645
26	8	0	0.255421	6.463706	-0.266792
27	8	0	1.134077	4.675325	1.511109
28	6	0	1.611216	3.767092	2.512537
29	6	0	5.237412	-2.651111	-2.672896
30	8	0	-3.695327	-0.320230	2.628298
31	6	0	-2.793651	0.592425	3.276939
32	1	0	-0.458269	-1.937134	-1.101714
33	1	0	0.511965	-1.506496	-2.508967
34	1	0	2.802243	-1.949298	-2.443030
35	1	0	0.680949	-1.505311	1.257154
36	1	0	4.875095	-2.772421	2.356091
37	1	0	-1.630255	1.372840	-1.742812
38	1	0	-1.543024	-0.063975	-2.761425

39	1	0	-1.826546	0.670187	0.920022
40	1	0	-3.341119	-1.463476	-2.484137
41	1	0	1.880970	0.717902	-1.627969
42	1	0	0.784104	0.975080	-2.983949
43	1	0	1.360510	2.121486	0.558197
44	1	0	0.002555	3.174649	-3.373718
45	1	0	-0.253298	5.540779	-2.648044
46	1	0	0.509197	6.545979	0.664975
47	1	0	2.561245	3.318083	2.210170
48	1	0	1.758942	4.368003	3.408613
49	1	0	0.873855	2.984647	2.711500
50	1	0	5.046607	-1.642919	-3.051429
51	1	0	6.261995	-2.939060	-2.904645
52	1	0	4.545222	-3.360853	-3.134674
53	1	0	-2.859647	1.584920	2.824427
54	1	0	-3.120683	0.635379	4.314094
55	1	0	-1.767171	0.221306	3.225717
56	8	0	2.846215	-2.212485	2.782677
57	6	0	1.689059	-1.937624	3.583740
58	1	0	0.877175	-2.630047	3.345425
59	1	0	2.004610	-2.084538	4.615625
60	1	0	1.354980	-0.905945	3.445058
61	8	0	-5.351791	-2.654393	-1.092702
62	6	0	-5.357868	-3.026551	-2.482177
63	1	0	-4.427939	-3.535339	-2.747300
64	1	0	-6.198717	-3.708094	-2.595197
65	1	0	-5.502864	-2.147425	-3.114566

----

5. ARO./X' (0,2)
   Sum of electronic and thermal Enthalpies= -1665.219500
   Frequencies -- 10.5257 17.6176 18.6284

Center Number	Atomic Number	Atomic Type	Coordinates (Angstroms) X	Y	Z
1	7	0	0.024763	0.076509	-1.164031
2	6	0	0.332477	-1.355596	-1.283644
3	6	0	1.550238	-1.790301	-0.500683
4	6	0	2.546055	-2.547946	-1.140555
5	6	0	1.648074	-1.474569	0.870059
6	6	0	3.646143	-2.994388	-0.431171
7	6	0	2.733563	-1.902736	1.604456
8	6	0	3.804396	-2.688716	0.990058
9	8	0	4.656196	-3.721741	-0.937572
10	8	0	4.799583	-3.080373	1.645539
11	6	0	-1.331628	0.361762	-1.671943
12	6	0	-2.444750	-0.206087	-0.815372
13	6	0	-2.629296	0.255095	0.493292
14	6	0	-3.310704	-1.168453	-1.340512
15	6	0	-3.669554	-0.245318	1.277988
16	6	0	-4.353361	-1.667092	-0.556767
17	6	0	-4.536751	-1.211803	0.751918
18	8	0	-5.556165	-1.690246	1.535731

19	6	0	1.036291	0.880481	-1.882859
20	6	0	0.966180	2.364671	-1.594472
21	6	0	1.259471	2.848276	-0.308026
22	6	0	0.638285	3.276499	-2.596543
23	6	0	1.214983	4.211154	-0.038255
24	6	0	0.594150	4.649052	-2.332919
25	6	0	0.877886	5.119349	-1.060884
26	8	0	0.830948	6.466946	-0.807777
27	8	0	1.482053	4.795170	1.171844
28	6	0	1.857653	3.955386	2.271334
29	6	0	4.616301	-4.096215	-2.325879
30	8	0	-3.937645	0.138895	2.563648
31	6	0	-3.106247	1.142197	3.160170
32	1	0	-0.528145	-1.911606	-0.896698
33	1	0	0.453248	-1.652664	-2.338200
34	1	0	2.439388	-2.770032	-2.194067
35	1	0	0.858406	-0.898527	1.331597
36	1	0	-1.435993	1.448543	-1.712530
37	1	0	-1.446360	-0.008005	-2.704575
38	1	0	-1.961628	1.007113	0.891617
39	1	0	-3.169421	-1.522979	-2.353152
40	1	0	-6.062949	-2.333036	1.016509
41	1	0	2.018036	0.505590	-1.580597
42	1	0	0.956241	0.720781	-2.971122
43	1	0	1.525862	2.150678	0.475726
44	1	0	0.412323	2.920879	-3.596159
45	1	0	0.337978	5.359501	-3.111326
46	1	0	1.058162	6.620026	0.121753
47	1	0	2.778515	3.409887	2.047535
48	1	0	2.025034	4.627847	3.111367
49	1	0	1.056190	3.253195	2.516551
50	1	0	4.619111	-3.208834	-2.963407
51	1	0	5.520990	-4.676975	-2.495015
52	1	0	3.735692	-4.708977	-2.533301
53	1	0	-3.162450	2.081582	2.602983
54	1	0	-3.501625	1.289951	4.164298
55	1	0	-2.067311	0.806631	3.222561
56	8	0	2.930902	-1.668638	2.914974
57	6	0	1.941769	-0.917907	3.639176
58	1	0	0.979289	-1.435153	3.623885
59	1	0	2.313524	-0.857401	4.660303
60	1	0	1.838178	0.086327	3.221043
61	8	0	-5.266616	-2.609395	-0.953595
62	6	0	-5.175691	-3.136826	-2.283912
63	1	0	-4.222400	-3.650367	-2.435896
64	1	0	-5.993546	-3.850388	-2.373181
65	1	0	-5.297343	-2.343919	-3.026931

----

6. ARO./Y (0,2)

Sum of electronic and thermal Enthalpies= -1665.212834

Frequencies -- 13.6679 15.1585 16.9695

Center Atomic Atomic Coordinates (Angstroms)

Number	Number	Type	X	Y	Z
1	7	0	0.035355	0.189155	-1.187617
2	6	0	0.395508	-1.229090	-1.392340
3	6	0	1.614462	-1.672199	-0.609823
4	6	0	2.795978	-2.009814	-1.274977
5	6	0	1.551585	-1.765911	0.785262
6	6	0	3.918531	-2.435144	-0.556485
7	6	0	2.668690	-2.190263	1.501412
8	6	0	3.856159	-2.525524	0.837041
9	8	0	5.119196	-2.783868	-1.113772
10	8	0	4.963486	-2.947993	1.527859
11	6	0	-1.317092	0.469556	-1.712885
12	6	0	-2.434922	-0.176504	-0.920523
13	6	0	-2.647056	0.182690	0.415566
14	6	0	-3.278977	-1.107232	-1.531198
15	6	0	-3.692044	-0.389143	1.142855
16	6	0	-4.327446	-1.676167	-0.805191
17	6	0	-4.536808	-1.324723	0.531444
18	8	0	-5.560013	-1.874964	1.261037
19	6	0	1.023019	1.072804	-1.825579
20	6	0	0.920653	2.516895	-1.395375
21	6	0	0.981276	2.853749	-0.036675
22	6	0	0.806511	3.542473	-2.372928
23	6	0	0.927702	4.180903	0.361800
24	6	0	0.746682	4.861274	-2.011747
25	6	0	0.800024	5.261804	-0.628212
26	8	0	0.744204	6.465821	-0.269027
27	8	0	0.990416	4.609595	1.628480
28	6	0	1.139700	3.648126	2.690132
29	6	0	5.257453	-2.702030	-2.537827
30	8	0	-3.985577	-0.106161	2.449113
31	6	0	-3.181465	0.864553	3.130781
32	1	0	-0.463298	-1.824864	-1.075277
33	1	0	0.553053	-1.439338	-2.463274
34	1	0	2.836763	-1.940121	-2.353926
35	1	0	0.634705	-1.514140	1.301569
36	1	0	4.748321	-2.964179	2.472624
37	1	0	-1.451981	1.554685	-1.691238
38	1	0	-1.396174	0.161029	-2.768446
39	1	0	-1.997427	0.911287	0.881239
40	1	0	-3.115739	-1.383805	-2.564670
41	1	0	-6.046557	-2.487912	0.689286
42	1	0	2.018228	0.710573	-1.547713
43	1	0	0.959837	1.015958	-2.924422
44	1	0	1.077084	2.064864	0.697240
45	1	0	0.761544	3.262677	-3.419994
46	1	0	0.652256	5.649276	-2.750813
47	1	0	2.070029	3.088644	2.569387
48	1	0	1.172735	4.233690	3.606606
49	1	0	0.286023	2.967079	2.712438
50	1	0	5.108534	-1.677112	-2.889385
51	1	0	6.278868	-3.015036	-2.750334
52	1	0	4.557112	-3.374914	-3.040754

53	1	0	-3.245612	1.840842	2.642133
54	1	0	-3.594990	0.932526	4.136162
55	1	0	-2.137792	0.542680	3.186177
56	8	0	2.734829	-2.329174	2.863277
57	6	0	1.563859	-2.042685	3.640097
58	1	0	0.739837	-2.704797	3.360846
59	1	0	1.846521	-2.226104	4.675663
60	1	0	1.262637	-0.998566	3.520879
61	8	0	-5.222394	-2.595459	-1.288247
62	6	0	-5.108697	-3.012126	-2.655636
63	1	0	-4.149751	-3.506081	-2.834495
64	1	0	-5.920012	-3.720952	-2.814671
65	1	0	-5.225121	-2.161983	-3.333206

----

7. Radical Cation (1,2)

Sum of electronic and thermal Enthalpies= -1665.639009

Frequencies -- 13.5166 15.0281 18.0763

Center Number	Atomic Number	Atomic Type	Coordinates (Angstroms) X	Y	Z
1	7	0	-0.031783	0.084684	-1.165258
2	6	0	0.499294	-1.279127	-1.392847
3	6	0	1.810836	-1.561913	-0.691776
4	6	0	2.964796	-1.802373	-1.442120
5	6	0	1.866962	-1.613275	0.705973
6	6	0	4.178311	-2.087594	-0.808067
7	6	0	3.075803	-1.896159	1.338328
8	6	0	4.235956	-2.132720	0.588171
9	8	0	5.359697	-2.332855	-1.452466
10	8	0	5.430873	-2.412829	1.198582
11	6	0	-1.402183	0.179116	-1.669535
12	6	0	-2.423504	-0.525536	-0.815334
13	6	0	-2.383607	-0.369882	0.587203
14	6	0	-3.433165	-1.280555	-1.446720
15	6	0	-3.344778	-0.962015	1.377731
16	6	0	-4.403922	-1.891027	-0.683413
17	6	0	-4.379818	-1.741102	0.749808
18	8	0	-5.292144	-2.297677	1.509153
19	6	0	0.829636	1.106977	-1.798555
20	6	0	0.497918	2.523144	-1.383334
21	6	0	0.754305	2.946832	-0.068264
22	6	0	-0.043960	3.433535	-2.290214
23	6	0	0.466105	4.248469	0.323572
24	6	0	-0.336973	4.744817	-1.902721
25	6	0	-0.088274	5.154861	-0.602738
26	8	0	-0.379795	6.441164	-0.228799
27	8	0	0.677004	4.771657	1.570805
28	6	0	1.264920	3.935114	2.576200
29	6	0	5.371872	-2.305071	-2.885569
30	8	0	-3.445652	-0.892565	2.705416
31	6	0	-2.470972	-0.118519	3.437242
32	1	0	-0.255900	-1.981750	-1.029109

33	1	0	0.618656	-1.468375	-2.471854
34	1	0	2.912656	-1.762886	-2.522036
35	1	0	0.972844	-1.438408	1.289129
36	1	0	5.297801	-2.389837	2.158581
37	1	0	-1.686172	1.239642	-1.683824
38	1	0	-1.483784	-0.178307	-2.708253
39	1	0	-1.597670	0.226952	1.025498
40	1	0	-3.429708	-1.381365	-2.523380
41	1	0	-5.942123	-2.787550	0.969692
42	1	0	1.857701	0.880008	-1.508979
43	1	0	0.780264	1.029066	-2.897004
44	1	0	1.184924	2.251204	0.640806
45	1	0	-0.240469	3.125722	-3.311625
46	1	0	-0.759249	5.454215	-2.606009
47	1	0	-0.148472	6.560502	0.704877
48	1	0	2.260825	3.602194	2.271339
49	1	0	1.342488	4.556496	3.467045
50	1	0	0.628361	3.070957	2.784985
51	1	0	5.088425	-1.318337	-3.262470
52	1	0	6.399627	-2.523132	-3.172712
53	1	0	4.706865	-3.068335	-3.299473
54	1	0	-2.513820	0.927780	3.128248
55	1	0	-2.756477	-0.215587	4.482000
56	1	0	-1.470694	-0.525849	3.278397
57	8	0	3.266538	-1.980900	2.692448
58	6	0	2.147190	-1.759903	3.560373
59	1	0	1.363926	-2.501874	3.382968
60	1	0	2.533690	-1.871286	4.572350
61	1	0	1.746163	-0.751285	3.429113
62	8	0	-5.415542	-2.647515	-1.107640
63	6	0	-5.572732	-2.890317	-2.523976
64	1	0	-4.686724	-3.393638	-2.915139
65	1	0	-6.443920	-3.535176	-2.607629
66	1	0	-5.745486	-1.947040	-3.045202

S1.3-Compound 3

ArOH (0,1)

Sum of electronic and thermal Enthalpies= -1616.879303

Frequencies -- 13.4166 14.0959 16.7797

Center Number	Atomic Number	Atomic Type	Coordinates (Angstroms) X	Y	Z
1	7	0	0.047508	0.131401	-1.254813
2	6	0	1.156800	-0.803632	-1.468494
3	6	0	2.236441	-0.692524	-0.425529
4	6	0	3.594067	-0.640295	-0.816419
5	6	0	1.917674	-0.686056	0.954415
6	6	0	4.589014	-0.599963	0.123972
7	6	0	2.898718	-0.626481	1.906502
8	6	0	4.308573	-0.570376	1.560886
9	8	0	5.189209	-0.464585	2.436250
10	6	0	-1.139728	-0.276518	-2.023691
11	6	0	-1.807905	-1.522449	-1.481417

12	6	0	-2.205417	-1.599147	-0.158533
13	6	0	-2.074492	-2.624817	-2.315925
14	6	0	-2.850258	-2.745014	0.331610
15	6	0	-2.719445	-3.756500	-1.849196
16	6	0	-3.126238	-3.848653	-0.513605
17	8	0	-3.745296	-4.975018	-0.125008
18	6	0	0.464096	1.510731	-1.592710
19	6	0	-0.515246	2.574674	-1.149779
20	6	0	-0.779311	2.768089	0.216902
21	6	0	-1.147812	3.396855	-2.081112
22	6	0	-1.662014	3.758168	0.631302
23	6	0	-2.033169	4.398066	-1.670679
24	6	0	-2.293925	4.580995	-0.322099
25	8	0	-3.163167	5.563748	0.075778
26	8	0	-1.995532	4.038964	1.929253
27	6	0	-1.392733	3.266432	2.976184
28	1	0	0.757632	-1.823958	-1.409051
29	1	0	1.598074	-0.696951	-2.471571
30	1	0	3.850253	-0.648117	-1.869238
31	1	0	0.877439	-0.732587	1.251579
32	1	0	2.663081	-0.613862	2.964529
33	1	0	-1.852597	0.550504	-1.982475
34	1	0	-0.894838	-0.431013	-3.086788
35	1	0	-2.027493	-0.776659	0.520250
36	1	0	-1.767451	-2.587668	-3.355862
37	1	0	-2.919972	-4.597383	-2.502488
38	1	0	-3.972090	-4.892418	0.825082
39	1	0	1.426261	1.682810	-1.100673
40	1	0	0.639107	1.608460	-2.676295
41	1	0	-0.287569	2.141296	0.949785
42	1	0	-0.953327	3.261369	-3.139655
43	1	0	-2.526285	5.038792	-2.393543
44	1	0	-3.228305	5.551979	1.042715
45	1	0	-0.306095	3.388199	2.972473
46	1	0	-1.802356	3.660135	3.905135
47	1	0	-1.652313	2.208802	2.878774
48	7	0	5.970274	-0.574220	-0.342600
49	8	0	6.811945	-1.191454	0.301722
50	8	0	6.222326	0.056314	-1.368421
51	7	0	-3.226335	-2.765971	1.719660
52	8	0	-2.967893	-1.803021	2.436869
53	8	0	-3.810022	-3.774908	2.166818

----

1. ArO-/X (-1,1)

Sum of electronic and thermal Enthalpies= -1616.427150

Frequencies -- 12.1894 17.1069 18.6028

Center Number	Atomic Number	Atomic Type	Coordinates (Angstroms) X	Y	Z
1	7	0	0.076677	-0.054809	-1.263579
2	6	0	0.961382	-1.227687	-1.411294
3	6	0	2.074375	-1.279510	-0.391711

4	6	0	3.393837	-1.211271	-0.759485
5	6	0	1.777980	-1.434243	0.994300
6	6	0	4.446852	-1.286292	0.191334
7	6	0	2.765648	-1.504851	1.935804
8	6	0	4.174778	-1.430013	1.614683
9	8	0	5.039679	-1.490045	2.537454
10	6	0	-1.162276	-0.231137	-2.039787
11	6	0	-2.099406	-1.267860	-1.456909
12	6	0	-2.671438	-1.082573	-0.210825
13	6	0	-2.436765	-2.433536	-2.170597
14	6	0	-3.553870	-2.034318	0.323139
15	6	0	-3.308250	-3.379412	-1.658958
16	6	0	-3.888724	-3.208179	-0.397920
17	8	0	-4.720636	-4.165248	0.044672
18	6	0	0.770703	1.187329	-1.656353
19	6	0	0.060667	2.450239	-1.216618
20	6	0	-0.080107	2.734929	0.152349
21	6	0	-0.439551	3.359200	-2.147303
22	6	0	-0.713139	3.899335	0.570486
23	6	0	-1.075786	4.534364	-1.733566
24	6	0	-1.216611	4.806379	-0.382490
25	8	0	-1.843715	5.959460	0.019145
26	8	0	-0.905554	4.282276	1.871949
27	6	0	-0.425587	3.427146	2.918404
28	1	0	0.336809	-2.118086	-1.294613
29	1	0	1.390899	-1.271735	-2.425945
30	1	0	3.658122	-1.098087	-1.802425
31	1	0	0.740050	-1.502257	1.306349
32	1	0	2.523185	-1.626469	2.986658
33	1	0	-1.677512	0.731864	-2.058458
34	1	0	-0.941230	-0.494297	-3.087449
35	1	0	-2.448875	-0.199618	0.373029
36	1	0	-2.001915	-2.597710	-3.151024
37	1	0	-3.557050	-4.273615	-2.218158
38	1	0	-5.045475	-3.905979	0.932788
39	1	0	1.761987	1.159082	-1.197616
40	1	0	0.926552	1.220286	-2.747598
41	1	0	0.312935	2.039577	0.883110
42	1	0	-0.336818	3.156537	-3.208111
43	1	0	-1.467478	5.242027	-2.456193
44	1	0	-1.856421	5.987333	0.987860
45	1	0	0.659190	3.305443	2.855086
46	1	0	-0.684065	3.928877	3.849748
47	1	0	-0.915133	2.450351	2.878348
48	7	0	5.764184	-1.209949	-0.308373
49	8	0	6.745252	-1.282265	0.460740
50	8	0	5.950145	-1.068623	-1.544351
51	7	0	-4.114269	-1.781610	1.623193
52	8	0	-3.815279	-0.755777	2.228512
53	8	0	-4.898914	-2.621925	2.111355

----

2. ARO-/X’ (-1,1)
   Sum of electronic and thermal Enthalpies= -1616.426089

Frequencies -- 8.9037 13.2335 14.6004

Center Number	Atomic Number	Atomic Type	Coordinates (Angstroms) X	Y	Z
1	7	0	-0.042988	0.079534	-1.267438
2	6	0	0.876586	-1.040764	-1.526522
3	6	0	2.011761	-1.124774	-0.530720
4	6	0	3.327067	-0.984520	-0.923592
5	6	0	1.750003	-1.381417	0.832976
6	6	0	4.374057	-1.096290	0.010712
7	6	0	2.765821	-1.493085	1.761234
8	6	0	4.106879	-1.355241	1.376367
9	8	0	5.044938	-1.483559	2.326866
10	6	0	-1.315326	-0.107800	-1.985990
11	6	0	-2.168635	-1.241260	-1.453303
12	6	0	-2.466733	-1.361464	-0.117644
13	6	0	-2.725012	-2.202567	-2.341745
14	6	0	-3.287529	-2.405942	0.377809
15	6	0	-3.538787	-3.210643	-1.899665
16	6	0	-3.889850	-3.391317	-0.510564
17	8	0	-4.657391	-4.338799	-0.164065
18	6	0	0.584316	1.365889	-1.624934
19	6	0	-0.170731	2.579964	-1.125865
20	6	0	-0.351735	2.780220	0.252861
21	6	0	-0.670619	3.529934	-2.014855
22	6	0	-1.023549	3.903278	0.721330
23	6	0	-1.343551	4.664548	-1.550214
24	6	0	-1.523614	4.854001	-0.189632
25	8	0	-2.186035	5.968867	0.260651
26	8	0	-1.258266	4.202074	2.037633
27	6	0	-0.801712	3.285526	3.041643
28	1	0	0.294323	-1.963546	-1.462658
29	1	0	1.289104	-0.992761	-2.547695
30	1	0	3.576250	-0.788141	-1.958001
31	1	0	0.723856	-1.501927	1.161977
32	1	0	2.554470	-1.698039	2.804097
33	1	0	5.925404	-1.371760	1.908018
34	1	0	-1.871596	0.829998	-1.903640
35	1	0	-1.138367	-0.270246	-3.062967
36	1	0	-2.065591	-0.652021	0.592180
37	1	0	-2.496321	-2.130842	-3.401706
38	1	0	-3.954795	-3.931967	-2.595640
39	1	0	1.587319	1.368543	-1.189270
40	1	0	0.714157	1.447392	-2.717306
41	1	0	0.037399	2.051509	0.952387
42	1	0	-0.540026	3.390465	-3.082742
43	1	0	-1.735402	5.403636	-2.240571
44	1	0	-2.233726	5.936500	1.228124
45	1	0	0.286948	3.186854	3.012674
46	1	0	-1.104970	3.717770	3.994054
47	1	0	-1.270388	2.305800	2.916074
48	7	0	5.720612	-0.943538	-0.459369
49	8	0	5.932086	-0.715819	-1.648487

50	8	0	6.658561	-1.045395	0.361204
51	7	0	-3.494798	-2.444349	1.776108
52	8	0	-4.165482	-3.359048	2.296538
53	8	0	-2.989971	-1.550371	2.500834

----

3. ARO-/Y (-1,1)

Sum of electronic and thermal Enthalpies= -1616.413571

Frequencies -- 15.3304 16.9600 19.0551

Center Number	Atomic Number	Atomic Type	Coordinates (Angstroms) X	Y	Z
1	7	0	0.025337	-0.076087	-1.260191
2	6	0	-0.872860	1.066662	-1.490849
3	6	0	-2.003820	1.149537	-0.489548
4	6	0	-3.323567	1.124926	-0.891672
5	6	0	-1.732505	1.290113	0.888948
6	6	0	-4.365597	1.234637	0.048442
7	6	0	-2.743493	1.393358	1.823383
8	6	0	-4.088947	1.365905	1.430262
9	8	0	-5.020610	1.469398	2.390109
10	6	0	1.289920	0.093863	-1.992108
11	6	0	2.165766	1.198302	-1.438967
12	6	0	2.602873	1.170431	-0.126425
13	6	0	2.580910	2.272360	-2.249155
14	6	0	3.425504	2.189836	0.377854
15	6	0	3.399569	3.279483	-1.768650
16	6	0	3.841600	3.268772	-0.441702
17	8	0	4.625752	4.280578	-0.034560
18	6	0	-0.635419	-1.350874	-1.628730
19	6	0	0.094443	-2.591311	-1.169351
20	6	0	0.173802	-2.908979	0.202735
21	6	0	0.686255	-3.470899	-2.073623
22	6	0	0.823676	-4.053073	0.642865
23	6	0	1.340994	-4.627498	-1.638413
24	6	0	1.446328	-4.976163	-0.271147
25	8	0	2.053213	-6.052043	0.150090
26	8	0	0.927019	-4.413362	1.973631
27	6	0	0.340250	-3.559031	2.954291
28	1	0	-0.276260	1.979356	-1.408132
29	1	0	-1.289901	1.051235	-2.511275
30	1	0	-3.580106	1.019628	-1.937532
31	1	0	-0.702552	1.320698	1.226008
32	1	0	-2.524296	1.502967	2.878964
33	1	0	-5.905111	1.433091	1.967279
34	1	0	1.831931	-0.852366	-1.925215
35	1	0	1.109858	0.282021	-3.063815
36	1	0	2.317254	0.362614	0.533417
37	1	0	2.249479	2.314450	-3.281443
38	1	0	3.709685	4.101192	-2.403339
39	1	0	4.845543	4.139216	0.910261
40	1	0	-1.632682	-1.325463	-1.180709
41	1	0	-0.782035	-1.400066	-2.721349

42	1	0	-0.288754	-2.240801	0.919860
43	1	0	0.637985	-3.257114	-3.138351
44	1	0	1.794710	-5.299497	-2.362296
45	1	0	-0.742509	-3.473441	2.813920
46	1	0	0.542017	-4.027741	3.917699
47	1	0	0.790610	-2.561004	2.936293
48	7	0	-5.717151	1.208773	-0.432143
49	8	0	-5.936500	1.104598	-1.637007
50	8	0	-6.650802	1.294134	0.394908
51	7	0	3.835730	2.108357	1.753722
52	8	0	4.558544	3.015036	2.218687
53	8	0	3.470087	1.161859	2.445317

----

4. ARO./X (0,2)

Sum of electronic and thermal Enthalpies= -1616.237055

Frequencies -- 9.1993 13.3384 14.5820

Center Number	Atomic Number	Atomic Type	Coordinates (Angstroms) X	Y	Z
1	7	0	0.047508	0.131401	-1.254813
2	6	0	1.156800	-0.803632	-1.468494
3	6	0	2.236441	-0.692524	-0.425529
4	6	0	3.594067	-0.640295	-0.816419
5	6	0	1.917674	-0.686056	0.954415
6	6	0	4.589014	-0.599963	0.123972
7	6	0	2.898718	-0.626481	1.906502
8	6	0	4.308573	-0.570376	1.560886
9	8	0	5.189209	-0.464585	2.436250
10	6	0	-1.139728	-0.276518	-2.023691
11	6	0	-1.807905	-1.522449	-1.481417
12	6	0	-2.205417	-1.599147	-0.158533
13	6	0	-2.074492	-2.624817	-2.315925
14	6	0	-2.850258	-2.745014	0.331610
15	6	0	-2.719445	-3.756500	-1.849196
16	6	0	-3.126238	-3.848653	-0.513605
17	8	0	-3.745296	-4.975018	-0.125008
18	6	0	0.464096	1.510731	-1.592710
19	6	0	-0.515246	2.574674	-1.149779
20	6	0	-0.779311	2.768089	0.216902
21	6	0	-1.147812	3.396855	-2.081112
22	6	0	-1.662014	3.758168	0.631302
23	6	0	-2.033169	4.398066	-1.670679
24	6	0	-2.293925	4.580995	-0.322099
25	8	0	-3.163167	5.563748	0.075778
26	8	0	-1.995532	4.038964	1.929253
27	6	0	-1.392733	3.266432	2.976184
28	1	0	0.757632	-1.823958	-1.409051
29	1	0	1.598074	-0.696951	-2.471571
30	1	0	3.850253	-0.648117	-1.869238
31	1	0	0.877439	-0.732587	1.251579
32	1	0	2.663081	-0.613862	2.964529
33	1	0	-1.852597	0.550504	-1.982475

34	1	0	-0.894838	-0.431013	-3.086788
35	1	0	-2.027493	-0.776659	0.520250
36	1	0	-1.767451	-2.587668	-3.355862
37	1	0	-2.919972	-4.597383	-2.502488
38	1	0	-3.972090	-4.892418	0.825082
39	1	0	1.426261	1.682810	-1.100673
40	1	0	0.639107	1.608460	-2.676295
41	1	0	-0.287569	2.141296	0.949785
42	1	0	-0.953327	3.261369	-3.139655
43	1	0	-2.526285	5.038792	-2.393543
44	1	0	-3.228305	5.551979	1.042715
45	1	0	-0.306095	3.388199	2.972473
46	1	0	-1.802356	3.660135	3.905135
47	1	0	-1.652313	2.208802	2.878774
48	7	0	5.970274	-0.574220	-0.342600
49	8	0	6.811945	-1.191454	0.301722
50	8	0	6.222326	0.056314	-1.368421
51	7	0	-3.226335	-2.765971	1.719660
52	8	0	-2.967893	-1.803021	2.436869
53	8	0	-3.810022	-3.774908	2.166818

----

5. ARO./X' (0,2)

Sum of electronic and thermal Enthalpies= -1616.238627

Frequencies -- 15.8930 16.6321 17.6834

Center Number	Atomic Number	Atomic Type	Coordinates (Angstroms) X	Y	Z
1	7	0	-0.040392	0.032180	-1.369402
2	6	0	0.956755	-1.023885	-1.633865
3	6	0	2.073051	-1.050939	-0.614856
4	6	0	3.382746	-0.811970	-0.977854
5	6	0	1.800547	-1.345723	0.738852
6	6	0	4.413733	-0.855748	-0.021093
7	6	0	2.801112	-1.390191	1.688542
8	6	0	4.135792	-1.142185	1.336451
9	8	0	5.056076	-1.194462	2.310257
10	6	0	-1.278953	-0.219617	-2.104915
11	6	0	-2.102264	-1.337291	-1.528728
12	6	0	-2.239529	-1.469182	-0.125314
13	6	0	-2.788771	-2.239318	-2.372687
14	6	0	-3.034384	-2.447527	0.405198
15	6	0	-3.574862	-3.236209	-1.856569
16	6	0	-3.747804	-3.419249	-0.426357
17	8	0	-4.420290	-4.363793	0.031271
18	6	0	0.502329	1.373062	-1.670538
19	6	0	-0.345036	2.505859	-1.134867
20	6	0	-0.497985	2.674505	0.251756
21	6	0	-0.964280	3.410201	-1.996363
22	6	0	-1.258911	3.722318	0.755676
23	6	0	-1.728680	4.469013	-1.496027
24	6	0	-1.880165	4.627960	-0.128031
25	8	0	-2.632226	5.667440	0.355533

26	8	0	-1.476590	3.984795	2.081534
27	6	0	-0.874387	3.126085	3.059730
28	1	0	0.436539	-1.985144	-1.603107
29	1	0	1.383429	-0.922838	-2.643949
30	1	0	3.638826	-0.585036	-2.004283
31	1	0	0.779345	-1.546967	1.043525
32	1	0	2.583281	-1.620371	2.724756
33	1	0	5.933581	-1.001125	1.915754
34	1	0	-1.893125	0.689572	-2.055965
35	1	0	-1.099503	-0.411140	-3.174723
36	1	0	-1.728311	-0.773392	0.526697
37	1	0	-2.678243	-2.139410	-3.446936
38	1	0	-4.086018	-3.945028	-2.497905
39	1	0	1.495702	1.424367	-1.218349
40	1	0	0.638410	1.502899	-2.756292
41	1	0	-0.014864	1.982041	0.929335
42	1	0	-0.852894	3.295739	-3.069298
43	1	0	-2.211591	5.174067	-2.163761
44	1	0	-2.636208	5.631069	1.323956
45	1	0	0.215906	3.151802	2.980851
46	1	0	-1.181620	3.521189	4.026799
47	1	0	-1.234099	2.099373	2.950565
48	7	0	5.755915	-0.598861	-0.458889
49	8	0	5.977398	-0.358531	-1.643300
50	8	0	6.677730	-0.625508	0.384714
51	7	0	-3.160401	-2.508270	1.857349
52	8	0	-4.245723	-2.821150	2.335653
53	8	0	-2.170715	-2.230895	2.534346

----

6. ARO./Y (0,2)

Sum of electronic and thermal Enthalpies= -1616.255846

Frequencies -- 12.2022 16.2814 16.7574

Center Number	Atomic Number	Atomic Type	Coordinates (Angstroms) X	Y	Z
1	7	0	0.085092	0.057038	-1.269600
2	6	0	0.955557	-1.120458	-1.436363
3	6	0	2.054865	-1.197472	-0.401292
4	6	0	3.385494	-1.172061	-0.766625
5	6	0	1.744465	-1.323721	0.970347
6	6	0	4.400495	-1.266611	0.203460
7	6	0	2.729186	-1.412957	1.933549
8	6	0	4.085494	-1.383758	1.577998
9	8	0	4.989492	-1.470743	2.564575
10	6	0	-1.145553	-0.077250	-2.071801
11	6	0	-2.097569	-1.130404	-1.546579
12	6	0	-2.628323	-1.033001	-0.272990
13	6	0	-2.492723	-2.220392	-2.345105
14	6	0	-3.529764	-1.995723	0.205069
15	6	0	-3.386364	-3.173897	-1.889988
16	6	0	-3.930232	-3.088662	-0.603717
17	8	0	-4.790377	-4.046617	-0.223251

18	6	0	0.792413	1.295089	-1.614052
19	6	0	0.132542	2.553807	-1.102246
20	6	0	-0.452372	2.595568	0.168629
21	6	0	0.164290	3.726936	-1.905776
22	6	0	-1.001577	3.775522	0.651078
23	6	0	-0.366801	4.905130	-1.457795
24	6	0	-0.981604	5.004146	-0.157663
25	8	0	-1.478780	6.075079	0.276205
26	8	0	-1.579021	3.923637	1.849269
27	6	0	-1.655636	2.796705	2.743498
28	1	0	0.324723	-2.008029	-1.340207
29	1	0	1.399264	-1.150829	-2.444262
30	1	0	3.670844	-1.078386	-1.806063
31	1	0	0.705493	-1.355906	1.278862
32	1	0	2.481203	-1.512350	2.983717
33	1	0	5.885744	-1.436668	2.166835
34	1	0	-1.652658	0.891000	-2.058174
35	1	0	-0.908947	-0.296983	-3.125092
36	1	0	-2.358039	-0.211589	0.375907
37	1	0	-2.087389	-2.317289	-3.346831
38	1	0	-3.682457	-4.008380	-2.514580
39	1	0	-5.086720	-3.852151	0.690461
40	1	0	1.792723	1.246102	-1.168091
41	1	0	0.945574	1.384314	-2.701759
42	1	0	-0.467382	1.695921	0.767814
43	1	0	0.616806	3.673947	-2.890364
44	1	0	-0.350327	5.803042	-2.065705
45	1	0	-0.654281	2.440089	2.994815
46	1	0	-2.152213	3.171726	3.636054
47	1	0	-2.246130	1.993316	2.297717
48	7	0	5.765283	-1.236656	-0.239361
49	8	0	6.016834	-1.141046	-1.438382
50	8	0	6.675389	-1.309133	0.613935
51	7	0	-4.039720	-1.837528	1.540935
52	8	0	-4.847713	-2.682899	1.977776
53	8	0	-3.674338	-0.884539	2.224287

----

7. Radical Cation (1,2)

Sum of electronic and thermal Enthalpies= -1616.679469

Frequencies -- 14.1887 20.6651 21.9661

Center Number	Atomic Number	Atomic Type	Coordinates (Angstroms) X	Y	Z
1	7	0	-0.143965	-0.273677	2.364658
2	6	0	-1.116957	-1.352405	2.162134
3	6	0	-1.934051	-1.228004	0.893702
4	6	0	-3.264699	-0.863284	0.944609
5	6	0	-1.362451	-1.542005	-0.356600
6	6	0	-4.028505	-0.803978	-0.231169
7	6	0	-2.104804	-1.487604	-1.517604
8	6	0	-3.457661	-1.115499	-1.488920
9	8	0	-4.114984	-1.083071	-2.652937

10	6	0	1.228854	-0.616817	2.741051
11	6	0	1.970683	-1.400587	1.674520
12	6	0	2.342846	-0.791209	0.489944
13	6	0	2.330115	-2.745545	1.883292
14	6	0	3.060389	-1.502774	-0.479146
15	6	0	3.045561	-3.453502	0.936757
16	6	0	3.431997	-2.853754	-0.270049
17	8	0	4.122640	-3.597616	-1.141538
18	6	0	-0.605981	1.111726	2.488603
19	6	0	0.177922	2.080643	1.627820
20	6	0	0.012321	2.063753	0.232130
21	6	0	1.042908	3.004401	2.210968
22	6	0	0.711321	2.964236	-0.561460
23	6	0	1.740615	3.914747	1.416255
24	6	0	1.583914	3.896668	0.037472
25	8	0	2.276822	4.786379	-0.729727
26	8	0	0.643766	3.049139	-1.921458
27	6	0	-0.213469	2.140908	-2.627978
28	1	0	-0.578775	-2.298287	2.186144
29	1	0	-1.781295	-1.315295	3.035199
30	1	0	-3.742052	-0.629284	1.886821
31	1	0	-0.321197	-1.839843	-0.407125
32	1	0	-1.665620	-1.734523	-2.476627
33	1	0	-5.044225	-0.819738	-2.481373
34	1	0	1.750892	0.305595	2.988077
35	1	0	1.147370	-1.222955	3.651724
36	1	0	2.095040	0.241926	0.290898
37	1	0	2.051765	-3.236570	2.809168
38	1	0	3.328124	-4.485613	1.105680
39	1	0	4.337148	-3.045833	-1.923738
40	1	0	-1.669395	1.131865	2.255276
41	1	0	-0.482387	1.372110	3.547919
42	1	0	-0.661252	1.348035	-0.220480
43	1	0	1.170183	3.026585	3.287236
44	1	0	2.408783	4.644627	1.859136
45	1	0	2.060112	4.639432	-1.663535
46	1	0	-1.255356	2.278448	-2.326521
47	1	0	-0.100052	2.390456	-3.681604
48	1	0	0.094292	1.106050	-2.458828
49	7	0	-5.414182	-0.426363	-0.117442
50	8	0	-5.883420	-0.164271	0.985213
51	8	0	-6.103810	-0.375078	-1.154710
52	7	0	3.405242	-0.815485	-1.699470
53	8	0	3.023647	0.336456	-1.876052
54	8	0	4.083214	-1.423089	-2.550747

S1.4-Compound 4

ArOH (0,1)

Sum of electronic and thermal Enthalpies= -2127.014323

Frequencies -- 14.7803 15.7337 20.6508

Center Number	Atomic Number	Atomic Type	Coordinates (Angstroms) X	Y	Z

1	7	0	0.025944	-0.190715	-1.278074
2	6	0	1.162870	-1.104797	-1.493185
3	6	0	2.308117	-0.886762	-0.527284
4	6	0	3.557119	-0.464913	-0.981985
5	6	0	2.150597	-1.130535	0.844296
6	6	0	4.610630	-0.289370	-0.088557
7	6	0	3.202011	-0.954636	1.732683
8	6	0	4.453568	-0.527912	1.278363
9	8	0	5.447063	-0.372072	2.200284
10	6	0	-1.175564	-0.671787	-1.984412
11	6	0	-1.782553	-1.917156	-1.373747
12	6	0	-2.310298	-1.882676	-0.079561
13	6	0	-1.855906	-3.118855	-2.085134
14	6	0	-2.880307	-3.020565	0.475041
15	6	0	-2.430319	-4.257171	-1.528070
16	6	0	-2.949979	-4.225472	-0.234367
17	8	0	-3.498408	-5.369103	0.267939
18	6	0	0.367825	1.183260	-1.695951
19	6	0	-0.607716	2.233940	-1.209098
20	6	0	-0.747204	2.477307	0.167886
21	6	0	-1.359252	2.991801	-2.105354
22	6	0	-1.624539	3.452074	0.627925
23	6	0	-2.242364	3.976223	-1.649515
24	6	0	-2.380055	4.207119	-0.290436
25	8	0	-3.250740	5.171147	0.152442
26	8	0	-1.841306	3.777952	1.941003
27	6	0	-1.128537	3.057217	2.955127
28	1	0	0.790084	-2.123724	-1.363406
29	1	0	1.537843	-1.032443	-2.527642
30	1	0	3.716938	-0.269454	-2.036205
31	1	0	1.192070	-1.468153	1.222718
32	1	0	3.077989	-1.149428	2.792221
33	1	0	6.271338	-0.090480	1.776956
34	1	0	-1.914420	0.132393	-1.950927
35	1	0	-0.959729	-0.860883	-3.049280
36	1	0	-2.279362	-0.967195	0.499237
37	1	0	-1.455887	-3.169545	-3.092285
38	1	0	-2.480839	-5.187059	-2.083462
39	1	0	-3.799306	-5.233790	1.178582
40	1	0	1.358108	1.405766	-1.290462
41	1	0	0.452562	1.248683	-2.793598
42	1	0	-0.161950	1.899401	0.871784
43	1	0	-1.262393	2.818622	-3.171907
44	1	0	-2.829877	4.565380	-2.345281
45	1	0	-3.225584	5.193999	1.121004
46	1	0	-0.049455	3.197557	2.847458
47	1	0	-1.459698	3.478691	3.903108
48	1	0	-1.372908	1.992027	2.920331
49	17	0	-3.549294	-2.955557	2.111442
50	17	0	6.181932	0.253203	-0.691658

----

1. ArO-/X (-1,1)

Sum of electronic and thermal Enthalpies= -2126.556199
Frequencies -- 10.8959 16.2465 18.5970

Center Number	Atomic Number	Atomic Type	Coordinates (Angstroms) X	Y	Z
1	7	0	0.000519	-0.262151	-1.193733
2	6	0	0.799443	-1.495376	-1.370933
3	6	0	1.984769	-1.605976	-0.439514
4	6	0	3.295360	-1.538211	-0.920916
5	6	0	1.820104	-1.799104	0.941482
6	6	0	4.383347	-1.648885	-0.062099
7	6	0	2.907445	-1.905776	1.795329
8	6	0	4.265259	-1.831612	1.349247
9	8	0	5.266637	-1.925696	2.164281
10	6	0	-1.282626	-0.363266	-1.909067
11	6	0	-2.257243	-1.340515	-1.285387
12	6	0	-2.713674	-1.147463	0.021759
13	6	0	-2.750831	-2.435350	-2.001803
14	6	0	-3.626162	-2.030685	0.582801
15	6	0	-3.669992	-3.315650	-1.439230
16	6	0	-4.122013	-3.126227	-0.133724
17	8	0	-5.021083	-4.019458	0.373256
18	6	0	0.753743	0.925957	-1.635938
19	6	0	0.149942	2.239610	-1.185183
20	6	0	0.083703	2.547690	0.184385
21	6	0	-0.322941	3.172104	-2.106902
22	6	0	-0.450412	3.757358	0.611991
23	6	0	-0.859291	4.392950	-1.683857
24	6	0	-0.927541	4.687637	-0.331925
25	8	0	-1.459111	5.884856	0.079071
26	8	0	-0.564570	4.165512	1.915440
27	6	0	-0.113224	3.284728	2.953330
28	1	0	0.123074	-2.336014	-1.194752
29	1	0	1.145689	-1.582745	-2.415406
30	1	0	3.471616	-1.396034	-1.982376
31	1	0	0.817208	-1.868713	1.354175
32	1	0	2.749976	-2.057416	2.859816
33	1	0	-1.735970	0.631352	-1.907699
34	1	0	-1.127263	-0.638750	-2.966102
35	1	0	-2.358730	-0.307274	0.606387
36	1	0	-2.411047	-2.606443	-3.017815
37	1	0	-4.046859	-4.164086	-1.999458
38	1	0	-5.256903	-3.789587	1.284182
39	1	0	1.760813	0.838376	-1.221136
40	1	0	0.862400	0.936569	-2.734002
41	1	0	0.456708	1.833971	0.907847
42	1	0	-0.277478	2.952089	-3.168278
43	1	0	-1.230218	5.118504	-2.399703
44	1	0	-1.431115	5.922981	1.046999
45	1	0	0.957028	3.083426	2.855587
46	1	0	-0.303347	3.810100	3.888133
47	1	0	-0.673699	2.346027	2.938139
48	17	0	-4.190606	-1.769077	2.239065

49	17	0	6.015485	-1.550540	-0.771796

----

2. ARO-/X' (-1,1)

Sum of electronic and thermal Enthalpies= -2126.555955

Frequencies -- 14.5251 18.6568 20.9314

Center Number	Atomic Number	Atomic Type	Coordinates (Angstroms) X	Y	Z
1	7	0	0.129603	-0.133084	-1.322299
2	6	0	1.226971	0.797207	-1.639029
3	6	0	1.290753	1.997807	-0.718895
4	6	0	1.078655	3.287131	-1.206552
5	6	0	1.591466	1.846421	0.642140
6	6	0	1.161788	4.386007	-0.355168
7	6	0	1.674051	2.943114	1.488379
8	6	0	1.458300	4.235600	1.000951
9	8	0	1.552555	5.273454	1.882117
10	6	0	0.329461	-1.435432	-1.996472
11	6	0	1.482860	-2.249289	-1.455424
12	6	0	1.421996	-2.818612	-0.176486
13	6	0	2.640322	-2.490480	-2.206955
14	6	0	2.471870	-3.578930	0.316937
15	6	0	3.688449	-3.256754	-1.711113
16	6	0	3.672909	-3.850747	-0.412991
17	8	0	4.653282	-4.562944	0.040272
18	6	0	-1.174986	0.451264	-1.686147
19	6	0	-2.363593	-0.279616	-1.096859
20	6	0	-2.535321	-0.331138	0.296857
21	6	0	-3.316912	-0.884641	-1.913955
22	6	0	-3.632646	-0.979986	0.850451
23	6	0	-4.425251	-1.537206	-1.363774
24	6	0	-4.585450	-1.589692	0.011323
25	8	0	-5.672260	-2.234859	0.547493
26	8	0	-3.904299	-1.092145	2.188998
27	6	0	-2.981968	-0.522671	3.127635
28	1	0	2.161019	0.238320	-1.545250
29	1	0	1.165315	1.141896	-2.685267
30	1	0	0.846476	3.443280	-2.253893
31	1	0	1.769640	0.855322	1.044595
32	1	0	1.913375	2.822365	2.539147
33	1	0	1.408252	6.120310	1.435213
34	1	0	-0.599709	-1.997407	-1.873674
35	1	0	0.468108	-1.289105	-3.081826
36	1	0	0.541839	-2.667247	0.439861
37	1	0	2.721435	-2.069884	-3.206040
38	1	0	4.572675	-3.426555	-2.319348
39	1	0	-1.181092	1.482341	-1.322741
40	1	0	-1.290116	0.501868	-2.782335
41	1	0	-1.803614	0.140788	0.940099
42	1	0	-3.200566	-0.854211	-2.992142
43	1	0	-5.166008	-2.011814	-1.998173
44	1	0	-5.619867	-2.184075	1.513894

45	1	0	-2.904704	0.559463	2.990597
46	1	0	-3.393901	-0.738327	4.112429
47	1	0	-1.995068	-0.983529	3.032245
48	17	0	0.880838	6.009787	-0.998507
49	17	0	2.309516	-4.269001	1.951959

----

3. ARO-/Y (-1,1)
Sum of electronic and thermal Enthalpies= -2126.548499
Frequencies -- 13.3214 16.8028 21.6722

Center Number	Atomic Number	Atomic Type	Coordinates (Angstroms) X	Y	Z
1	7	0	0.038389	-0.079890	-1.229827
2	6	0	1.445493	-0.449447	-1.464260
3	6	0	2.418247	0.223100	-0.518432
4	6	0	3.392753	1.098437	-0.996966
5	6	0	2.384586	-0.041012	0.857915
6	6	0	4.299335	1.691513	-0.122086
7	6	0	3.288118	0.552581	1.728020
8	6	0	4.262869	1.433262	1.249726
9	8	0	5.122280	1.985470	2.154634
10	6	0	-0.868659	-1.016325	-1.916174
11	6	0	-0.901265	-2.397456	-1.296806
12	6	0	-1.390336	-2.577232	0.000741
13	6	0	-0.469382	-3.525729	-2.001358
14	6	0	-1.430917	-3.845138	0.564754
15	6	0	-0.513713	-4.795899	-1.434712
16	6	0	-0.993555	-4.974334	-0.137663
17	8	0	-1.011693	-6.239130	0.374647
18	6	0	-0.217215	1.315133	-1.658741
19	6	0	-1.544864	1.879337	-1.208530
20	6	0	-1.786582	2.140119	0.156541
21	6	0	-2.557225	2.185854	-2.115525
22	6	0	-2.991464	2.676549	0.587311
23	6	0	-3.773589	2.729603	-1.689814
24	6	0	-4.054175	2.999622	-0.329750
25	8	0	-5.183049	3.508988	0.083103
26	8	0	-3.278379	2.954557	1.911200
27	6	0	-2.281492	2.675093	2.892970
28	1	0	1.527149	-1.531044	-1.330190
29	1	0	1.742335	-0.235465	-2.504967
30	1	0	3.450462	1.325021	-2.055477
31	1	0	1.642103	-0.724334	1.254994
32	1	0	3.261160	0.342830	2.791618
33	1	0	5.754713	2.573575	1.716596
34	1	0	-1.870871	-0.584230	-1.869879
35	1	0	-0.610162	-1.107711	-2.984935
36	1	0	-1.742751	-1.728165	0.574420
37	1	0	-0.089147	-3.412435	-3.011131
38	1	0	-0.173758	-5.666067	-1.985171
39	1	0	-1.338885	-6.234867	1.286253
40	1	0	0.594850	1.921049	-1.247445

41	1	0	-0.144158	1.394949	-2.757363
42	1	0	-1.006884	1.916610	0.875387
43	1	0	-2.400543	2.001337	-3.175300
44	1	0	-4.548241	2.961838	-2.416111
45	1	0	-1.371524	3.258315	2.716377
46	1	0	-2.715161	2.965927	3.850032
47	1	0	-2.032263	1.609006	2.917989
48	17	0	5.521912	2.801731	-0.755855
49	17	0	-2.059998	-4.053061	2.205141

----

4. ARO./X (0,2)
   Sum of electronic and thermal Enthalpies= -2126.386138
   Frequencies -- 15.7943 17.2134 22.5400

Center Number	Atomic Number	Atomic Type	Coordinates (Angstroms) X	Y	Z
1	7	0	0.101527	-0.144154	-1.270804
2	6	0	1.335113	-0.912180	-1.480332
3	6	0	2.408111	-0.595800	-0.470418
4	6	0	3.709529	-0.267546	-0.896188
5	6	0	2.127059	-0.670495	0.920351
6	6	0	4.699694	-0.016158	0.023652
7	6	0	3.098356	-0.426138	1.848859
8	6	0	4.452153	-0.078987	1.462731
9	8	0	5.344670	0.146688	2.311119
10	6	0	-1.032711	-0.756934	-1.986316
11	6	0	-1.489345	-2.070125	-1.388190
12	6	0	-1.974565	-2.119789	-0.077944
13	6	0	-1.467573	-3.255039	-2.130614
14	6	0	-2.413961	-3.321154	0.461463
15	6	0	-1.915470	-4.456183	-1.590151
16	6	0	-2.396866	-4.507673	-0.282194
17	8	0	-2.823422	-5.711264	0.197110
18	6	0	0.292434	1.265125	-1.676081
19	6	0	-0.804762	2.194203	-1.204281
20	6	0	-0.999045	2.412625	0.170163
21	6	0	-1.617289	2.868006	-2.114430
22	6	0	-1.988598	3.281488	0.614116
23	6	0	-2.612127	3.747184	-1.674845
24	6	0	-2.801645	3.955784	-0.318245
25	8	0	-3.779156	4.818895	0.107834
26	8	0	-2.268870	3.572407	1.922939
27	6	0	-1.483231	2.950968	2.948941
28	1	0	1.090460	-1.975383	-1.373412
29	1	0	1.732047	-0.779242	-2.498967
30	1	0	3.924300	-0.208379	-1.956938
31	1	0	1.125432	-0.933732	1.237953
32	1	0	2.897898	-0.490110	2.912569
33	1	0	-1.859461	-0.043697	-1.947495
34	1	0	-0.793249	-0.909512	-3.051419
35	1	0	-2.012784	-1.220834	0.525603
36	1	0	-1.095545	-3.241328	-3.149562

37	1	0	-1.896757	-5.371993	-2.170319
38	1	0	-3.128640	-5.630667	1.112678
39	1	0	1.244556	1.595051	-1.250732
40	1	0	0.388796	1.344974	-2.771245
41	1	0	-0.368235	1.899899	0.885008
42	1	0	-1.479457	2.711698	-3.179020
43	1	0	-3.245515	4.272561	-2.381323
44	1	0	-3.767234	4.854810	1.076398
45	1	0	-0.429772	3.228087	2.853865
46	1	0	-1.877763	3.328984	3.890924
47	1	0	-1.589306	1.863246	2.915646
48	17	0	-3.020674	-3.360494	2.122481
49	17	0	6.306667	0.399111	-0.508576

----

5. ARO./X' (0,2)
   Sum of electronic and thermal Enthalpies= -2126.386321
   Frequencies -- 14.9944 15.7916 18.7258

Center Number	Atomic Number	Atomic Type	Coordinates (Angstroms) X	Y	Z
1	7	0	0.050466	-0.251420	-1.271722
2	6	0	1.232473	-1.113677	-1.479788
3	6	0	2.358864	-0.842367	-0.506612
4	6	0	3.586374	-0.353919	-0.953610
5	6	0	2.206082	-1.099193	0.862937
6	6	0	4.624290	-0.126800	-0.053853
7	6	0	3.241296	-0.870138	1.757881
8	6	0	4.470860	-0.376139	1.311886
9	8	0	5.448910	-0.172066	2.240008
10	6	0	-1.106524	-0.781923	-1.997109
11	6	0	-1.699785	-2.023675	-1.384407
12	6	0	-1.867001	-2.123390	0.014629
13	6	0	-2.147472	-3.080900	-2.214818
14	6	0	-2.449953	-3.235864	0.563791
15	6	0	-2.733697	-4.197590	-1.684063
16	6	0	-2.922319	-4.350598	-0.255519
17	8	0	-3.455817	-5.371536	0.235216
18	6	0	0.331379	1.142944	-1.670768
19	6	0	-0.714757	2.132743	-1.206518
20	6	0	-0.888854	2.378091	0.166164
21	6	0	-1.501055	2.832097	-2.120686
22	6	0	-1.832781	3.298891	0.604981
23	6	0	-2.451225	3.761839	-1.686320
24	6	0	-2.621155	3.996927	-0.331525
25	8	0	-3.554943	4.910401	0.087566
26	8	0	-2.088102	3.621124	1.911426
27	6	0	-1.321499	2.980897	2.940429
28	1	0	0.905560	-2.149596	-1.354916
29	1	0	1.608932	-1.020437	-2.511272
30	1	0	3.740307	-0.147012	-2.006488
31	1	0	1.264963	-1.488880	1.234654
32	1	0	3.121875	-1.074032	2.816183

33	1	0	6.253598	0.172883	1.826246
34	1	0	-1.888775	-0.012534	-1.990723
35	1	0	-0.873714	-0.976821	-3.056490
36	1	0	-1.532070	-1.311450	0.647008
37	1	0	-2.012834	-2.996614	-3.287794
38	1	0	-3.073971	-5.014178	-2.310958
39	1	0	1.295640	1.412316	-1.233349
40	1	0	0.444266	1.220371	-2.764665
41	1	0	-0.277717	1.844907	0.883366
42	1	0	-1.376930	2.656950	-3.184040
43	1	0	-3.064373	4.307217	-2.395462
44	1	0	-3.540237	4.956231	1.055597
45	1	0	-0.257279	3.206467	2.829940
46	1	0	-1.687883	3.393124	3.879422
47	1	0	-1.478666	1.898953	2.927263
48	17	0	6.170450	0.494655	-0.645603
49	17	0	-2.661750	-3.353994	2.292446

----

6. ARO./Y (0,2)

Sum of electronic and thermal Enthalpies= -2126.391105

Frequencies -- 14.5190 17.5147 19.2737

Center Number	Atomic Number	Atomic Type	Coordinates (Angstroms) X	Y	Z
1	7	0	0.106438	-0.117647	-1.221323
2	6	0	1.233790	-1.051982	-1.404756
3	6	0	2.370095	-0.822762	-0.431879
4	6	0	3.625211	-0.415193	-0.883229
5	6	0	2.197933	-1.037757	0.942809
6	6	0	4.670754	-0.225844	0.016469
7	6	0	3.241446	-0.847661	1.837425
8	6	0	4.499176	-0.435180	1.386568
9	8	0	5.483935	-0.264411	2.314529
10	6	0	-1.089558	-0.577330	-1.955314
11	6	0	-1.708053	-1.837319	-1.389290
12	6	0	-2.214971	-1.856914	-0.086623
13	6	0	-1.813862	-3.001336	-2.157423
14	6	0	-2.796976	-3.010782	0.420588
15	6	0	-2.402602	-4.154323	-1.648639
16	6	0	-2.902717	-4.176770	-0.346949
17	8	0	-3.465890	-5.332930	0.106813
18	6	0	0.476414	1.238878	-1.647961
19	6	0	-0.475348	2.313006	-1.177554
20	6	0	-0.865626	2.376314	0.166077
21	6	0	-0.938122	3.295171	-2.095148
22	6	0	-1.704589	3.388686	0.607584
23	6	0	-1.765726	4.307158	-1.690584
24	6	0	-2.200596	4.416972	-0.320637
25	8	0	-2.961189	5.336485	0.076842
26	8	0	-2.133374	3.541258	1.866339
27	6	0	-1.712595	2.602013	2.874041
28	1	0	0.843757	-2.061557	-1.256038

29	1	0	1.619744	-1.007130	-2.436270
30	1	0	3.795914	-0.242738	-1.939731
31	1	0	1.234694	-1.364386	1.318857
32	1	0	3.106454	-1.019925	2.899494
33	1	0	6.313699	0.005655	1.894253
34	1	0	-1.826047	0.229910	-1.910757
35	1	0	-0.858574	-0.738635	-3.021016
36	1	0	-2.158850	-0.972793	0.536738
37	1	0	-1.427792	-3.009808	-3.171185
38	1	0	-2.479213	-5.054502	-2.248156
39	1	0	-3.765067	-5.231599	1.022547
40	1	0	1.463287	1.462434	-1.227821
41	1	0	0.580555	1.300811	-2.743249
42	1	0	-0.500063	1.627318	0.854913
43	1	0	-0.627106	3.228948	-3.132333
44	1	0	-2.127082	5.057293	-2.385405
45	1	0	-0.626601	2.626322	2.988683
46	1	0	-2.191843	2.932396	3.793296
47	1	0	-2.046272	1.593488	2.619953
48	17	0	6.250051	0.296060	-0.582750
49	17	0	-3.431377	-3.017903	2.071738

----

7. Radical Cation (1,2)

Sum of electronic and thermal Enthalpies= -2126.817971

Frequencies -- 10.9221 12.1089 16.0180

Center Number	Atomic Number	Atomic Type	Coordinates (Angstroms) X	Y	Z
1	7	0	-0.260045	-1.456961	-2.110548
2	6	0	0.440663	-2.628594	-1.574228
3	6	0	1.417214	-2.296234	-0.463564
4	6	0	2.787975	-2.257551	-0.717182
5	6	0	0.959765	-2.059005	0.838851
6	6	0	3.677204	-1.982480	0.314794
7	6	0	1.852271	-1.790545	1.865618
8	6	0	3.227815	-1.744356	1.618960
9	8	0	4.050543	-1.472558	2.664441
10	6	0	-1.724041	-1.464760	-2.189049
11	6	0	-2.404799	-1.553419	-0.837477
12	6	0	-2.483292	-0.422839	-0.020818
13	6	0	-2.990546	-2.746547	-0.403213
14	6	0	-3.132708	-0.498375	1.203131
15	6	0	-3.638773	-2.816125	0.822645
16	6	0	-3.716330	-1.692239	1.648145
17	8	0	-4.361004	-1.818923	2.836369
18	6	0	0.498994	-0.419119	-2.813573
19	6	0	0.225497	0.983283	-2.306141
20	6	0	0.734794	1.387300	-1.060704
21	6	0	-0.495284	1.886971	-3.084327
22	6	0	0.526651	2.685687	-0.612415
23	6	0	-0.705289	3.191858	-2.636651
24	6	0	-0.200942	3.594867	-1.408475

25	8	0	-0.409665	4.875549	-0.984948
26	8	0	0.975994	3.202671	0.568874
27	6	0	1.744671	2.361558	1.441306
28	1	0	-0.309542	-3.339354	-1.234202
29	1	0	0.975902	-3.069234	-2.424099
30	1	0	3.169234	-2.451779	-1.712592
31	1	0	-0.101901	-2.092738	1.053892
32	1	0	1.503393	-1.614086	2.876580
33	1	0	4.980167	-1.479709	2.390647
34	1	0	-2.033304	-0.573041	-2.730661
35	1	0	-1.984771	-2.344768	-2.790485
36	1	0	-2.045868	0.516463	-0.336805
37	1	0	-2.949004	-3.628383	-1.032562
38	1	0	-4.099033	-3.737826	1.159406
39	1	0	-4.355176	-0.982750	3.325958
40	1	0	1.554825	-0.673938	-2.740371
41	1	0	0.199855	-0.493981	-3.866653
42	1	0	1.296250	0.686551	-0.456941
43	1	0	-0.884359	1.583443	-4.049544
44	1	0	-1.254404	3.906725	-3.239012
45	1	0	0.014148	5.001070	-0.122030
46	1	0	2.661766	2.027734	0.948605
47	1	0	1.994172	2.981276	2.300996
48	1	0	1.156370	1.499679	1.765927
49	17	0	-3.239806	0.936513	2.224432
50	17	0	5.408368	-1.938659	-0.017122

S1.5- Compound 5

ArOH (0,1)

Sum of electronic and thermal Enthalpies= -6354.854309

Frequencies -- 10.9334 14.5599 14.8375

Center Number	Atomic Number	Atomic Type	Coordinates (Angstroms) X	Y	Z
1	7	0	0.025342	-0.202722	-1.272058
2	6	0	1.167011	-1.111572	-1.486962
3	6	0	2.310918	-0.888728	-0.520785
4	6	0	3.550283	-0.434587	-0.971952
5	6	0	2.161979	-1.158715	0.846783
6	6	0	4.603402	-0.254689	-0.077922
7	6	0	3.212094	-0.978694	1.735208
8	6	0	4.454507	-0.520980	1.284459
9	8	0	5.445505	-0.366516	2.210517
10	6	0	-1.169531	-0.687002	-1.987368
11	6	0	-1.780802	-1.932384	-1.380830
12	6	0	-2.325160	-1.894336	-0.093525
13	6	0	-1.845993	-3.135664	-2.090472
14	6	0	-2.906344	-3.030383	0.455461
15	6	0	-2.431788	-4.270618	-1.539944
16	6	0	-2.971704	-4.235144	-0.253929
17	8	0	-3.530960	-5.381236	0.233625
18	6	0	0.364011	1.173788	-1.686352
19	6	0	-0.617939	2.220028	-1.203390

20	6	0	-0.740574	2.484452	0.171266
21	6	0	-1.392534	2.952987	-2.100809
22	6	0	-1.623088	3.456125	0.628074
23	6	0	-2.282259	3.932882	-1.648246
24	6	0	-2.402372	4.185384	-0.291291
25	8	0	-3.276798	5.147571	0.148137
26	8	0	-1.822744	3.802539	1.938677
27	6	0	-1.065640	3.126334	2.951385
28	1	0	0.799103	-2.132381	-1.358436
29	1	0	1.541507	-1.035981	-2.521235
30	1	0	3.695735	-0.220216	-2.024223
31	1	0	1.211076	-1.521105	1.221682
32	1	0	3.095454	-1.194126	2.791567
33	1	0	6.263699	-0.061918	1.790651
34	1	0	-1.910357	0.115449	-1.961159
35	1	0	-0.943984	-0.877223	-3.049916
36	1	0	-2.296892	-0.974412	0.478095
37	1	0	-1.432228	-3.189230	-3.091887
38	1	0	-2.478257	-5.201316	-2.094385
39	1	0	-3.879211	-5.241515	1.126853
40	1	0	1.350926	1.399524	-1.275129
41	1	0	0.453957	1.240130	-2.783502
42	1	0	-0.136442	1.927045	0.875906
43	1	0	-1.307899	2.764119	-3.165728
44	1	0	-2.887562	4.502783	-2.344801
45	1	0	-3.236875	5.188910	1.115693
46	1	0	0.006306	3.290447	2.811009
47	1	0	-1.382495	3.564057	3.896884
48	1	0	-1.283843	2.054889	2.950445
49	35	0	-3.655674	-2.951370	2.230075
50	35	0	6.303991	0.376871	-0.729234

----

1. ArO-/X (-1,1)

Sum of electronic and thermal Enthalpies= -6354.394495

Frequencies -- 13.8160 17.8752 21.4072

Center Number	Atomic Number	Atomic Type	Coordinates (Angstroms) X	Y	Z
1	7	0	-0.011091	-0.261199	-1.210316
2	6	0	0.800624	-1.486877	-1.378130
3	6	0	1.975523	-1.588005	-0.432366
4	6	0	3.292083	-1.577653	-0.904032
5	6	0	1.795071	-1.723676	0.953415
6	6	0	4.372693	-1.691164	-0.034737
7	6	0	2.872913	-1.829763	1.818185
8	6	0	4.239433	-1.814456	1.383756
9	8	0	5.220955	-1.907872	2.215732
10	6	0	-1.285993	-0.373425	-1.938765
11	6	0	-2.261134	-1.353428	-1.320057
12	6	0	-2.764973	-1.130375	-0.035083
13	6	0	-2.706945	-2.480625	-2.017499
14	6	0	-3.678062	-2.016171	0.523150

15	6	0	-3.624334	-3.363638	-1.457857
16	6	0	-4.124625	-3.144353	-0.174078
17	8	0	-5.020865	-4.046908	0.323431
18	6	0	0.736378	0.934002	-1.645008
19	6	0	0.127909	2.240020	-1.179126
20	6	0	0.105655	2.550899	0.191246
21	6	0	-0.395735	3.160685	-2.085201
22	6	0	-0.433980	3.752154	0.635297
23	6	0	-0.940426	4.372008	-1.645449
24	6	0	-0.963966	4.669816	-0.292647
25	8	0	-1.499996	5.859365	0.134879
26	8	0	-0.504613	4.163145	1.940825
27	6	0	0.026219	3.305802	2.960548
28	1	0	0.128562	-2.333244	-1.211518
29	1	0	1.160344	-1.571819	-2.418324
30	1	0	3.474764	-1.481008	-1.969286
31	1	0	0.787975	-1.748854	1.361001
32	1	0	2.703740	-1.935541	2.886685
33	1	0	-1.745938	0.617908	-1.947025
34	1	0	-1.117225	-0.653247	-2.992624
35	1	0	-2.443872	-0.262434	0.528209
36	1	0	-2.330550	-2.674806	-3.016292
37	1	0	-3.964243	-4.237280	-2.002858
38	1	0	-5.300212	-3.797122	1.216845
39	1	0	1.744769	0.846359	-1.233673
40	1	0	0.840773	0.954191	-2.743246
41	1	0	0.518547	1.846537	0.902279
42	1	0	-0.383291	2.939071	-3.147152
43	1	0	-1.350508	5.088575	-2.348887
44	1	0	-1.435679	5.902037	1.101058
45	1	0	1.095332	3.134767	2.808010
46	1	0	-0.129942	3.834460	3.899779
47	1	0	-0.506074	2.350985	2.982843
48	35	0	-4.361876	-1.684706	2.295260
49	35	0	6.152211	-1.679418	-0.807232

----

2. ARO-/X’ (-1,1)

Sum of electronic and thermal Enthalpies= -6354.394281

Frequencies -- 13.7212 15.8744 20.2447

Center Number	Atomic Number	Atomic Type	Coordinates (Angstroms) X	Y	Z
1	7	0	0.128591	-0.169258	-1.264866
2	6	0	1.231411	0.751762	-1.590229
3	6	0	1.299373	1.964500	-0.686472
4	6	0	1.119198	3.249326	-1.198895
5	6	0	1.576333	1.830681	0.681510
6	6	0	1.212159	4.361345	-0.364581
7	6	0	1.666494	2.939369	1.510499
8	6	0	1.484526	4.227955	0.997989
9	8	0	1.588534	5.275106	1.868494
10	6	0	0.312203	-1.474745	-1.940744

11	6	0	1.504200	-2.270655	-1.461586
12	6	0	1.516888	-2.862924	-0.190913
13	6	0	2.634329	-2.466530	-2.266760
14	6	0	2.609295	-3.599047	0.247309
15	6	0	3.724924	-3.205218	-1.827544
16	6	0	3.788862	-3.819070	-0.536643
17	8	0	4.814875	-4.496447	-0.147866
18	6	0	-1.169266	0.428474	-1.634012
19	6	0	-2.372185	-0.294634	-1.065546
20	6	0	-2.586767	-0.318035	0.322868
21	6	0	-3.299853	-0.916899	-1.899278
22	6	0	-3.701498	-0.955358	0.855205
23	6	0	-4.424198	-1.559331	-1.370721
24	6	0	-4.627383	-1.583184	-0.000442
25	8	0	-5.732096	-2.215310	0.514070
26	8	0	-4.016547	-1.036789	2.186516
27	6	0	-3.133290	-0.430752	3.139702
28	1	0	2.162561	0.190222	-1.487117
29	1	0	1.174046	1.083579	-2.640924
30	1	0	0.905701	3.384939	-2.252736
31	1	0	1.730047	0.843757	1.103590
32	1	0	1.886581	2.832133	2.566938
33	1	0	1.457892	6.118108	1.409658
34	1	0	-0.602858	-2.046625	-1.768279
35	1	0	0.393016	-1.330075	-3.032357
36	1	0	0.657766	-2.746136	0.461312
37	1	0	2.659585	-2.029293	-3.261792
38	1	0	4.587080	-3.338783	-2.475663
39	1	0	-1.169792	1.456424	-1.262282
40	1	0	-1.271629	0.488108	-2.731103
41	1	0	-1.876832	0.169784	0.978750
42	1	0	-3.150774	-0.906791	-2.973801
43	1	0	-5.145232	-2.046802	-2.017953
44	1	0	-5.710595	-2.145626	1.480523
45	1	0	-3.060741	0.647554	2.973088
46	1	0	-3.578112	-0.621209	4.115341
47	1	0	-2.139649	-0.884627	3.093196
48	35	0	0.955994	6.124002	-1.102346
49	35	0	2.523283	-4.382611	2.019170

----

3. ARO-/Y (-1,1)

Sum of electronic and thermal Enthalpies= -6354.388485

Frequencies -- 11.6925 12.4954 16.7623

Center Number	Atomic Number	Atomic Type	Coordinates (Angstroms) X	Y	Z
1	7	0	0.041485	-0.087755	-1.213793
2	6	0	1.449333	-0.456323	-1.447929
3	6	0	2.422463	0.217972	-0.503958
4	6	0	3.380940	1.111052	-0.983137
5	6	0	2.405748	-0.061094	0.869787
6	6	0	4.289558	1.705570	-0.110469

7	6	0	3.310654	0.533555	1.737122
8	6	0	4.270511	1.430839	1.258088
9	8	0	5.132128	1.979646	2.164165
10	6	0	-0.861759	-1.019493	-1.911165
11	6	0	-0.897751	-2.406348	-1.304910
12	6	0	-1.391689	-2.597502	-0.010666
13	6	0	-0.469753	-3.529055	-2.020683
14	6	0	-1.443331	-3.872642	0.538415
15	6	0	-0.525918	-4.804967	-1.469921
16	6	0	-1.013775	-4.996209	-0.177042
17	8	0	-1.041445	-6.271536	0.310969
18	6	0	-0.212097	1.309937	-1.638438
19	6	0	-1.543502	1.871194	-1.196740
20	6	0	-1.780569	2.167125	0.161963
21	6	0	-2.565872	2.137314	-2.105553
22	6	0	-2.990971	2.697163	0.585564
23	6	0	-3.788773	2.671217	-1.686940
24	6	0	-4.065628	2.973339	-0.332695
25	8	0	-5.202142	3.470413	0.073393
26	8	0	-3.272666	3.012711	1.902124
27	6	0	-2.268414	2.770193	2.886126
28	1	0	1.532037	-1.537705	-1.313016
29	1	0	1.744939	-0.242921	-2.489057
30	1	0	3.419217	1.345204	-2.040515
31	1	0	1.675905	-0.757979	1.266814
32	1	0	3.297568	0.312058	2.798583
33	1	0	5.755088	2.578160	1.725832
34	1	0	-1.864590	-0.589166	-1.865511
35	1	0	-0.598407	-1.101504	-2.979500
36	1	0	-1.738770	-1.748303	0.565786
37	1	0	-0.084955	-3.406591	-3.027586
38	1	0	-0.191127	-5.671389	-2.029417
39	1	0	-1.401082	-6.287263	1.210389
40	1	0	0.595723	1.914615	-1.217840
41	1	0	-0.130170	1.393091	-2.736150
42	1	0	-0.992574	1.976773	0.881487
43	1	0	-2.412143	1.926978	-3.160921
44	1	0	-4.572088	2.869871	-2.413874
45	1	0	-1.364378	3.355829	2.688140
46	1	0	-2.699603	3.084771	3.836785
47	1	0	-2.009739	1.707421	2.940251
48	35	0	5.602536	2.936888	-0.800791
49	35	0	-2.135296	-4.112718	2.322335

----

4. ARO./X (0,2)

Sum of electronic and thermal Enthalpies= -6354.226384

Frequencies -- 12.5970 13.9783 18.7327

Center Number	Atomic Number	Atomic Type	Coordinates (Angstroms) X	Y	Z
1	7	0	0.106858	-0.147519	-1.223963
2	6	0	1.327519	-0.933535	-1.449246

3	6	0	2.430542	-0.612124	-0.473638
4	6	0	3.708276	-0.247976	-0.942505
5	6	0	2.205316	-0.712257	0.924877
6	6	0	4.730123	0.012787	-0.059786
7	6	0	3.208283	-0.462636	1.817824
8	6	0	4.541642	-0.081334	1.387120
9	8	0	5.456338	0.143911	2.209958
10	6	0	-1.038109	-0.741007	-1.940329
11	6	0	-1.503192	-2.057309	-1.355754
12	6	0	-2.018792	-2.109926	-0.057114
13	6	0	-1.457078	-3.242225	-2.097374
14	6	0	-2.463824	-3.314824	0.472156
15	6	0	-1.908979	-4.445828	-1.567443
16	6	0	-2.420667	-4.500782	-0.270495
17	8	0	-2.846055	-5.712043	0.192907
18	6	0	0.318364	1.259324	-1.628050
19	6	0	-0.780320	2.200352	-1.184838
20	6	0	-1.024198	2.406228	0.183636
21	6	0	-1.544990	2.899107	-2.117576
22	6	0	-2.015946	3.286804	0.598905
23	6	0	-2.540191	3.791387	-1.706462
24	6	0	-2.779269	3.987302	-0.355827
25	8	0	-3.755982	4.864203	0.042763
26	8	0	-2.343412	3.565985	1.899203
27	6	0	-1.636425	2.888934	2.946839
28	1	0	1.072851	-1.991285	-1.318851
29	1	0	1.698911	-0.819907	-2.479856
30	1	0	3.872600	-0.169665	-2.010678
31	1	0	1.222609	-1.000643	1.278724
32	1	0	3.051566	-0.547257	2.887528
33	1	0	-1.857749	-0.020845	-1.887909
34	1	0	-0.805031	-0.882592	-3.008374
35	1	0	-2.074147	-1.207255	0.539355
36	1	0	-1.062241	-3.226419	-3.107611
37	1	0	-1.871202	-5.361539	-2.146820
38	1	0	-3.194305	-5.635665	1.093583
39	1	0	1.263866	1.581829	-1.182457
40	1	0	0.439494	1.335510	-2.721017
41	1	0	-0.431336	1.874623	0.916966
42	1	0	-1.369398	2.751428	-3.177833
43	1	0	-3.136433	4.335883	-2.430546
44	1	0	-3.785759	4.881752	1.011394
45	1	0	-0.570496	3.130848	2.917461
46	1	0	-2.069187	3.253970	3.877072
47	1	0	-1.777332	1.806978	2.874770
48	35	0	-3.169689	-3.359646	2.265581
49	35	0	6.442354	0.521702	-0.713909

----

5. ARO./X' (0,2)

Sum of electronic and thermal Enthalpies= -6354.226525

Frequencies -- 13.5792 15.1676 16.8761

Center Atomic Atomic Coordinates (Angstroms)

Number	Number	Type	X	Y	Z
1	7	0	0.032699	-0.249655	-1.280154
2	6	0	1.211809	-1.116058	-1.485584
3	6	0	2.339742	-0.847811	-0.513181
4	6	0	3.570362	-0.369555	-0.963637
5	6	0	2.187200	-1.099001	0.857515
6	6	0	4.611535	-0.147317	-0.065499
7	6	0	3.224808	-0.874658	1.750269
8	6	0	4.457991	-0.391549	1.300681
9	8	0	5.436105	-0.192713	2.231130
10	6	0	-1.122988	-0.774841	-2.015545
11	6	0	-1.710838	-2.021608	-1.406601
12	6	0	-1.987610	-2.069006	-0.020832
13	6	0	-2.033794	-3.136254	-2.216672
14	6	0	-2.554597	-3.184920	0.538185
15	6	0	-2.598837	-4.260277	-1.676807
16	6	0	-2.893385	-4.363067	-0.259826
17	8	0	-3.403294	-5.395595	0.229041
18	6	0	0.319670	1.145889	-1.672614
19	6	0	-0.720804	2.138123	-1.200512
20	6	0	-0.879282	2.387352	0.173419
21	6	0	-1.517711	2.835193	-2.107274
22	6	0	-1.818221	3.308926	0.620652
23	6	0	-2.463220	3.765784	-1.664311
24	6	0	-2.617853	4.004044	-0.308216
25	8	0	-3.545165	4.919747	0.120112
26	8	0	-2.057200	3.634797	1.929277
27	6	0	-1.284683	2.988772	2.950197
28	1	0	0.881106	-2.150492	-1.358170
29	1	0	1.588836	-1.026960	-2.517227
30	1	0	3.717782	-0.169143	-2.018371
31	1	0	1.244087	-1.481025	1.232083
32	1	0	3.105505	-1.074015	2.809446
33	1	0	6.246067	0.136053	1.813505
34	1	0	-1.904021	-0.005301	-2.005108
35	1	0	-0.883112	-0.963880	-3.074150
36	1	0	-1.747892	-1.207162	0.588430
37	1	0	-1.819362	-3.092806	-3.279008
38	1	0	-2.842617	-5.122878	-2.286977
39	1	0	1.285636	1.408405	-1.235014
40	1	0	0.431081	1.228074	-2.766241
41	1	0	-0.258733	1.857994	0.885391
42	1	0	-1.405221	2.658169	-3.171619
43	1	0	-3.084609	4.309196	-2.367773
44	1	0	-3.521690	4.962762	1.088163
45	1	0	-0.220699	3.212249	2.833072
46	1	0	-1.643444	3.397728	3.893533
47	1	0	-1.444413	1.907220	2.933626
48	35	0	6.301674	0.512701	-0.715097
49	35	0	-2.934956	-3.228350	2.404252

6. ARO./Y (0,2)

Sum of electronic and thermal Enthalpies= -6354.230920

Frequencies -- 11.8280 14.5405 17.1143

Center Number	Atomic Number	Atomic Type	Coordinates (Angstroms) X	Y	Z
1	7	0	0.107484	-0.127672	-1.235485
2	6	0	1.242558	-1.053260	-1.418521
3	6	0	2.372254	-0.823655	-0.438311
4	6	0	3.620095	-0.380758	-0.877448
5	6	0	2.201029	-1.073228	0.930588
6	6	0	4.660214	-0.191979	0.029679
7	6	0	3.238275	-0.884273	1.831921
8	6	0	4.489344	-0.437880	1.393411
9	8	0	5.466289	-0.273876	2.331959
10	6	0	-1.079720	-0.592859	-1.981744
11	6	0	-1.708903	-1.845532	-1.411215
12	6	0	-2.293255	-1.827460	-0.141210
13	6	0	-1.749392	-3.038133	-2.140755
14	6	0	-2.888296	-2.972803	0.372816
15	6	0	-2.348047	-4.182246	-1.625269
16	6	0	-2.926707	-4.167375	-0.355629
17	8	0	-3.495915	-5.322187	0.096846
18	6	0	0.471370	1.234063	-1.653675
19	6	0	-0.485054	2.300639	-1.174847
20	6	0	-0.845173	2.374221	0.176855
21	6	0	-0.982351	3.265294	-2.092777
22	6	0	-1.686427	3.381478	0.626212
23	6	0	-1.816463	4.269248	-1.681485
24	6	0	-2.221639	4.388850	-0.303447
25	8	0	-2.988722	5.300220	0.100080
26	8	0	-2.083519	3.548322	1.893403
27	6	0	-1.618208	2.635402	2.905440
28	1	0	0.859554	-2.066568	-1.278163
29	1	0	1.632883	-0.998808	-2.447813
30	1	0	3.782445	-0.183224	-1.930502
31	1	0	1.243177	-1.426882	1.296035
32	1	0	3.104985	-1.083870	2.889388
33	1	0	6.292527	0.020772	1.920757
34	1	0	-1.815887	0.214873	-1.955183
35	1	0	-0.833383	-0.764126	-3.042383
36	1	0	-2.286573	-0.916205	0.444547
37	1	0	-1.305283	-3.075799	-3.129722
38	1	0	-2.374907	-5.104763	-2.194396
39	1	0	-3.864449	-5.200296	0.984481
40	1	0	1.457082	1.458669	-1.232200
41	1	0	0.573907	1.303132	-2.748495
42	1	0	-0.452947	1.638408	0.865548
43	1	0	-0.692951	3.191880	-3.135685
44	1	0	-2.205164	5.005403	-2.376490
45	1	0	-0.529603	2.676949	2.985247
46	1	0	-2.073376	2.977306	3.832688
47	1	0	-1.944917	1.617447	2.681603
48	35	0	6.372638	0.423408	-0.604988

49	35	0	-3.695214	-2.920809	2.122874

----

7. Radical Cation (1,2)

Sum of electronic and thermal Enthalpies= -6354.657527

Frequencies -- 2.5859 7.5008 17.7685

Center Number	Atomic Number	Atomic Type	Coordinates (Angstroms) X	Y	Z
1	7	0	-0.233508	-1.441815	-2.063668
2	6	0	0.476946	-2.618569	-1.553920
3	6	0	1.501677	-2.301302	-0.485174
4	6	0	2.864674	-2.370923	-0.771938
5	6	0	1.095393	-1.977600	0.815361
6	6	0	3.798085	-2.120822	0.228546
7	6	0	2.029864	-1.732218	1.809042
8	6	0	3.399166	-1.798367	1.530586
9	8	0	4.258876	-1.546403	2.552353
10	6	0	-1.694750	-1.477250	-2.167350
11	6	0	-2.401126	-1.582549	-0.829520
12	6	0	-2.487508	-0.465731	0.005481
13	6	0	-3.012808	-2.775500	-0.432116
14	6	0	-3.174180	-0.553071	1.209518
15	6	0	-3.697667	-2.856999	0.772112
16	6	0	-3.787515	-1.745794	1.614315
17	8	0	-4.473214	-1.894078	2.777970
18	6	0	0.516801	-0.381384	-2.743930
19	6	0	0.185477	1.012977	-2.252028
20	6	0	0.683680	1.449915	-1.013069
21	6	0	-0.573279	1.878755	-3.036802
22	6	0	0.423937	2.743359	-0.577160
23	6	0	-0.837521	3.177632	-2.600022
24	6	0	-0.346335	3.612437	-1.377633
25	8	0	-0.607483	4.885959	-0.962141
26	8	0	0.857041	3.293963	0.594797
27	6	0	1.676204	2.503306	1.468288
28	1	0	-0.264896	-3.325091	-1.186542
29	1	0	0.971549	-3.065357	-2.426030
30	1	0	3.197313	-2.629893	-1.769799
31	1	0	0.039515	-1.924825	1.054136
32	1	0	1.720725	-1.488604	2.819035
33	1	0	5.180188	-1.631917	2.264130
34	1	0	-2.012640	-0.590819	-2.712470
35	1	0	-1.928712	-2.360728	-2.774263
36	1	0	-2.025335	0.468812	-0.286411
37	1	0	-2.963981	-3.647597	-1.074240
38	1	0	-4.179258	-3.778106	1.079559
39	1	0	-4.487637	-1.067597	3.283755
40	1	0	1.577071	-0.602256	-2.633712
41	1	0	0.254970	-0.471221	-3.806239
42	1	0	1.276177	0.778150	-0.405796
43	1	0	-0.951779	1.551420	-3.998383
44	1	0	-1.418518	3.863005	-3.206701

45	1	0	-0.187174	5.032034	-0.100594
46	1	0	2.603183	2.209307	0.968801
47	1	0	1.903720	3.145364	2.317580
48	1	0	1.135341	1.617162	1.810319
49	35	0	-3.300341	0.995550	2.344632
50	35	0	5.674209	-2.226396	-0.179479

S2. Values of pKa obtained using ChemAxon pKa calculator

<table>
<tr><th>Compound</th><th colspan="2">Reference (H)</th><th colspan="2">Compound 1 (one o-OCH_3)</th><th colspan="2">Compound 2 (two o-OCH_3)</th><th colspan="2">Compound 3 (o-NO_2)</th><th colspan="2">Compound 4 (o-Cl)</th><th colspan="2">Compound 5 (o-Br)</th></tr>
<tr><td>Phenol ring</td><td>pKa</td><td><pKa></td><td>pKa</td><td><pKa></td><td>pKa</td><td><pKa></td><td>pKa</td><td><pKa></td><td>pKa</td><td><pKa></td><td>pKa</td><td><pKa></td></tr>
<tr><td>X</td><td>9.63</td><td rowspan="2">9.38</td><td>9.93</td><td rowspan="3">9.93</td><td>9.5</td><td rowspan="2">9.24</td><td>7.17</td><td rowspan="2">6.65</td><td>8.29</td><td rowspan="2">8.03</td><td>8.46</td><td rowspan="2">8.19</td></tr>
<tr><td>X'</td><td>9.13</td><td>9.45</td><td>8.97</td><td>6.13</td><td>7.77</td><td>7.92</td></tr>
<tr><td>Y</td><td>10.17</td><td>10.17</td><td>10.41</td><td>10.10</td><td>10.10</td><td>9.93</td><td>9.93</td><td>9.94</td><td>9.94</td><td>9.94</td><td>9.94</td></tr>
</table>

S3. Multistep DFT calculations in methanol:

Information ordered as follows:

Molecule (Charge, spin multiplicity), Enthalpies (au), lowest frequencies (cm^{-1}), and, optimized geometries (Cartesian, Å) of Compounds **1** to **5** as well as reference obtained at the B3LYP/6-311++G**

S3.0 - Reference

2nd PA (-1,2)

Sum of electronic and thermal Enthalpies= -1206.661375

Frequencies -- 13.2817 17.2390 23.3618

Center Number	Atomic Number	Atomic Type	Coordinates (Angstroms) X	Y	Z
1	7	0	0.423142	0.018611	-0.994775
2	6	0	1.596965	0.848419	-1.295322
3	6	0	1.578840	2.189927	-0.614831
4	6	0	1.784436	3.377239	-1.356410
5	6	0	1.395970	2.281722	0.789737
6	6	0	1.798653	4.605921	-0.743730
7	6	0	1.406694	3.494001	1.427661
8	6	0	1.605013	4.722374	0.686038
9	8	0	1.612491	5.847421	1.262001
10	6	0	0.659094	-1.387290	-1.403501
11	6	0	1.732121	-2.095055	-0.612485
12	6	0	1.531426	-2.456659	0.730506
13	6	0	2.962316	-2.445649	-1.188209
14	6	0	2.504869	-3.122651	1.463535
15	6	0	3.948874	-3.115209	-0.470404
16	6	0	3.765230	-3.483537	0.894633
17	8	0	4.680760	-4.112181	1.578827
18	6	0	-0.778354	0.565635	-1.658325
19	6	0	-2.080630	-0.040265	-1.179799
20	6	0	-2.484991	0.121596	0.156056
21	6	0	-2.914545	-0.734376	-2.054869
22	6	0	-3.692432	-0.406433	0.597857
23	6	0	-4.132833	-1.264475	-1.618201
24	6	0	-4.524195	-1.105080	-0.298693
25	8	0	-5.720910	-1.627044	0.124515
26	8	0	-4.187806	-0.310389	1.871692
27	6	0	-3.410255	0.376604	2.861326
28	1	0	2.479249	0.303500	-0.937381
29	1	0	1.729795	0.987429	-2.380924
30	1	0	1.926275	3.305707	-2.429926
31	1	0	1.252347	1.370500	1.358808
32	1	0	1.275049	3.569688	2.501692
33	1	0	-0.293435	-1.908615	-1.279203
34	1	0	0.907868	-1.432620	-2.478007
35	1	0	0.586318	-2.213265	1.209955
36	1	0	3.150145	-2.189261	-2.228383
37	1	0	4.889944	-3.372171	-0.949732
38	1	0	-0.796713	1.641360	-1.461009
39	1	0	-0.707009	0.445586	-2.752457
40	1	0	-1.849027	0.664378	0.843659

41	1	0	-2.618120	-0.868757	-3.089706
42	1	0	-4.782167	-1.805921	-2.297603
43	1	0	-5.838364	-1.422074	1.064407
44	1	0	-3.262777	1.423787	2.583280
45	1	0	-3.990009	0.320771	3.781477
46	1	0	-2.443174	-0.112781	3.005425
47	1	0	1.949113	5.519124	-1.309268
48	1	0	2.316245	-3.391596	2.499697

2nd ETE (0,3)

Sum of electronic and thermal Enthalpies= -1206.495467

Frequencies -- 9.4953 20.5840 23.3799

Center Number	Atomic Number	Atomic Type	Coordinates (Angstroms) X	Y	Z
1	7	0	0.457590	0.070890	-1.069477
2	6	0	1.603849	0.942654	-1.366504
3	6	0	1.578023	2.250274	-0.616758
4	6	0	1.800241	3.469431	-1.298757
5	6	0	1.379288	2.271434	0.787893
6	6	0	1.816979	4.666020	-0.626098
7	6	0	1.392122	3.450260	1.485324
8	6	0	1.608183	4.712043	0.806259
9	8	0	1.615838	5.805793	1.436691
10	6	0	0.718209	-1.302235	-1.519691
11	6	0	1.720781	-2.036736	-0.668347
12	6	0	1.596293	-2.049639	0.745021
13	6	0	2.773857	-2.762852	-1.272518
14	6	0	2.478974	-2.748388	1.525428
15	6	0	3.673836	-3.471062	-0.515548
16	6	0	3.568535	-3.494369	0.928638
17	8	0	4.390446	-4.139607	1.637089
18	6	0	-0.778612	0.610397	-1.678693
19	6	0	-2.043638	-0.057535	-1.188052
20	6	0	-2.450198	0.100441	0.147992
21	6	0	-2.836119	-0.816994	-2.047720
22	6	0	-3.619017	-0.495570	0.605249
23	6	0	-4.014009	-1.418989	-1.594321
24	6	0	-4.406678	-1.264442	-0.274534
25	8	0	-5.561713	-1.856957	0.166145
26	8	0	-4.115442	-0.407613	1.878201
27	6	0	-3.397537	0.373137	2.843141
28	1	0	2.516382	0.412318	-1.069176
29	1	0	1.692242	1.139334	-2.446649
30	1	0	1.952892	3.449122	-2.372815
31	1	0	1.221560	1.334196	1.308328
32	1	0	1.247716	3.474869	2.559986
33	1	0	-0.227919	-1.853465	-1.464675
34	1	0	1.037288	-1.330958	-2.573696
35	1	0	0.782910	-1.500512	1.204228
36	1	0	2.867165	-2.748934	-2.353469
37	1	0	4.487031	-4.024684	-0.972024

38	1	0	-0.816802	1.675061	-1.431760
39	1	0	-0.732163	0.537983	-2.777359
40	1	0	-1.847157	0.695348	0.822049
41	1	0	-2.539152	-0.944808	-3.083144
42	1	0	-4.631587	-2.011046	-2.260855
43	1	0	-5.682337	-1.649370	1.105207
44	1	0	-3.334898	1.418512	2.528716
45	1	0	-3.972292	0.301644	3.765269
46	1	0	-2.394355	-0.031341	3.003280
47	1	0	1.978925	5.605189	-1.143669
48	1	0	2.385970	-2.770293	2.605800

3rd PA (-1,3)

Sum of electronic and thermal Enthalpies= -1206.038847

Frequencies -- 18.1811 21.3803 23.9982

Center Number	Atomic Number	Atomic Type	Coordinates (Angstroms) X	Y	Z
1	7	0	0.400982	0.027787	-0.997441
2	6	0	1.591817	0.838114	-1.290632
3	6	0	1.558336	2.203302	-0.660466
4	6	0	1.782022	3.362700	-1.439231
5	6	0	1.340420	2.346313	0.734620
6	6	0	1.781114	4.613095	-0.871260
7	6	0	1.335242	3.581233	1.328191
8	6	0	1.551643	4.781775	0.547651
9	8	0	1.544473	5.927707	1.081792
10	6	0	0.632663	-1.398692	-1.342351
11	6	0	1.688501	-2.075196	-0.507453
12	6	0	1.479437	-2.353524	0.854971
13	6	0	2.914970	-2.476727	-1.059893
14	6	0	2.441996	-2.986190	1.628409
15	6	0	3.890823	-3.113312	-0.301818
16	6	0	3.700297	-3.394315	1.084264
17	8	0	4.607530	-3.985239	1.806338
18	6	0	-0.769588	0.543044	-1.722380
19	6	0	-2.089925	0.000483	-1.230328
20	6	0	-2.380090	-0.035753	0.139731
21	6	0	-3.069614	-0.420284	-2.170303
22	6	0	-3.615630	-0.480319	0.585492
23	6	0	-4.298116	-0.864160	-1.761572
24	6	0	-4.646966	-0.923223	-0.364655
25	8	0	-5.768598	-1.328084	0.037802
26	8	0	-3.994477	-0.548724	1.868396
27	6	0	-3.076198	-0.136186	2.898405
28	1	0	2.454976	0.298331	-0.882291
29	1	0	1.761586	0.934320	-2.375265
30	1	0	1.951545	3.252747	-2.505460
31	1	0	1.184621	1.456767	1.333981
32	1	0	1.177157	3.695365	2.395222
33	1	0	-0.328332	-1.905496	-1.209415
34	1	0	0.895999	-1.490689	-2.409627

35	1	0	0.536785	-2.069591	1.316026
36	1	0	3.106782	-2.284393	-2.112921
37	1	0	4.829875	-3.410174	-0.761196
38	1	0	-0.792190	1.631653	-1.594585
39	1	0	-0.683801	0.357446	-2.805392
40	1	0	-1.626941	0.290359	0.843441
41	1	0	-2.827082	-0.388375	-3.227225
42	1	0	-5.048747	-1.191765	-2.472441
43	1	0	-2.817091	0.918270	2.779363
44	1	0	-3.609067	-0.285074	3.835379
45	1	0	-2.175920	-0.754304	2.878266
46	1	0	1.946137	5.504948	-1.466015
47	1	0	2.249622	-3.190256	2.678429

3rd ETE (0,4)

Sum of electronic and thermal Enthalpies= -1205.870860

Frequencies -- 15.2725 18.5440 20.6350

Center Number	Atomic Number	Atomic Type	Coordinates (Angstroms) X	Y	Z
1	7	0	0.438086	0.055271	-1.096713
2	6	0	1.593276	0.922132	-1.374152
3	6	0	1.535191	2.253083	-0.667827
4	6	0	1.813165	3.445755	-1.375569
5	6	0	1.251750	2.323691	0.720194
6	6	0	1.805323	4.664291	-0.743402
7	6	0	1.237756	3.525661	1.377524
8	6	0	1.511672	4.761094	0.671390
9	8	0	1.497839	5.875293	1.264465
10	6	0	0.715927	-1.333213	-1.500156
11	6	0	1.691333	-2.037079	-0.590734
12	6	0	1.443994	-2.111480	0.804081
13	6	0	2.843253	-2.662478	-1.120887
14	6	0	2.305172	-2.772376	1.639569
15	6	0	3.726062	-3.329355	-0.307274
16	6	0	3.496791	-3.412878	1.120067
17	8	0	4.300839	-4.020922	1.879617
18	6	0	-0.771327	0.560945	-1.766942
19	6	0	-2.055083	-0.034529	-1.236289
20	6	0	-2.316305	-0.036094	0.139134
21	6	0	-3.019528	-0.553037	-2.142105
22	6	0	-3.512814	-0.542725	0.626147
23	6	0	-4.206336	-1.065536	-1.692947
24	6	0	-4.524472	-1.097336	-0.287133
25	8	0	-5.603827	-1.569994	0.151847
26	8	0	-3.867493	-0.578590	1.915260
27	6	0	-2.968487	-0.054248	2.910866
28	1	0	2.493219	0.403788	-1.022943
29	1	0	1.725811	1.083861	-2.455223
30	1	0	2.029851	3.387401	-2.437117
31	1	0	1.051336	1.407085	1.261959
32	1	0	1.029142	3.588227	2.439991

33	1	0	-0.229995	-1.884940	-1.463888
34	1	0	1.074051	-1.385107	-2.539772
35	1	0	0.552964	-1.641963	1.204164
36	1	0	3.027878	-2.603541	-2.188350
37	1	0	4.615369	-3.805273	-0.705540
38	1	0	-0.813729	1.643780	-1.606738
39	1	0	-0.718557	0.405012	-2.855556
40	1	0	-1.575361	0.369113	0.814218
41	1	0	-2.797867	-0.542938	-3.203914
42	1	0	-4.945097	-1.470746	-2.375531
43	1	0	-2.782597	1.007762	2.735729
44	1	0	-3.480336	-0.190950	3.861211
45	1	0	-2.029268	-0.611574	2.909321
46	1	0	2.010773	5.583261	-1.281328
47	1	0	2.119633	-2.841888	2.705834

S3.1- Compound 1

2nd PA (-1,2)

Sum of electronic and thermal Enthalpies= -1435.710312

Frequencies -- 11.3891 15.3565 20.0376

Center Number	Atomic Number	Atomic Type	Coordinates (Angstroms) X	Y	Z
1	7	0	0.084138	-0.144655	-1.116823
2	6	0	1.183532	-1.109981	-1.248835
3	6	0	2.296728	-0.889572	-0.257000
4	6	0	3.615741	-0.737380	-0.693820
5	6	0	2.009829	-0.878176	1.138486
6	6	0	4.649401	-0.568906	0.221032
7	6	0	3.004226	-0.715513	2.061802
8	6	0	4.380275	-0.548707	1.665045
9	8	0	5.940414	-0.408337	-0.095937
10	8	0	5.313096	-0.396313	2.497273
11	6	0	-1.125686	-0.622073	-1.828664
12	6	0	-1.748161	-1.865811	-1.241028
13	6	0	-2.367951	-1.829311	0.025934
14	6	0	-1.754301	-3.077177	-1.931954
15	6	0	-2.955020	-2.959893	0.574680
16	6	0	-2.348063	-4.219821	-1.388534
17	6	0	-2.971639	-4.223915	-0.117890
18	8	0	-3.528639	-5.279938	0.404127
19	6	0	0.498771	1.183310	-1.613415
20	6	0	-0.445901	2.306208	-1.240261
21	6	0	-0.617549	2.661874	0.108069
22	6	0	-1.135465	3.022731	-2.216965
23	6	0	-1.466790	3.703715	0.462077
24	6	0	-1.989914	4.073620	-1.868319
25	6	0	-2.160855	4.414610	-0.536274
26	8	0	-3.004152	5.444323	-0.200209
27	8	0	-1.708789	4.137436	1.739143
28	6	0	-1.046354	3.476930	2.825804
29	6	0	6.334578	-0.409267	-1.481019

30	8	0	-3.573593	-2.982556	1.809309
31	6	0	-3.603113	-1.778695	2.574989
32	1	0	0.763785	-2.105882	-1.067040
33	1	0	1.594678	-1.119561	-2.271719
34	1	0	3.821060	-0.747771	-1.756754
35	1	0	0.983742	-1.009784	1.461346
36	1	0	2.795339	-0.714863	3.126145
37	1	0	-1.845409	0.200288	-1.799773
38	1	0	-0.893739	-0.803312	-2.892473
39	1	0	-2.381644	-0.893724	0.572774
40	1	0	-1.289396	-3.135700	-2.912724
41	1	0	-2.341175	-5.153220	-1.945315
42	1	0	1.483890	1.395869	-1.188954
43	1	0	0.622562	1.166610	-2.709493
44	1	0	-0.080058	2.118633	0.874821
45	1	0	-1.013455	2.762904	-3.263158
46	1	0	-2.530271	4.629476	-2.626912
47	1	0	-3.018922	5.537629	0.764283
48	1	0	0.039163	3.574646	2.737159
49	1	0	-1.386838	3.982190	3.728420
50	1	0	-1.325110	2.420562	2.867995
51	1	0	5.854460	0.413155	-2.015976
52	1	0	7.413416	-0.267851	-1.473300
53	1	0	6.086364	-1.364606	-1.948948
54	1	0	-4.149339	-0.986112	2.052869
55	1	0	-4.124638	-2.025887	3.499865
56	1	0	-2.592043	-1.430837	2.811328

2nd ETE

Sum of electronic and thermal Enthalpies= -1435.551680

Frequencies -- 8.3223 12.9887 15.0727

Center Number	Atomic Number	Atomic Type	Coordinates (Angstroms) X	Y	Z
1	7	0	0.106485	-0.167873	-1.145036
2	6	0	1.271293	-1.057887	-1.281723
3	6	0	2.369708	-0.770564	-0.286881
4	6	0	3.673883	-0.537989	-0.727845
5	6	0	2.085185	-0.783548	1.109340
6	6	0	4.699346	-0.312808	0.185442
7	6	0	3.069795	-0.565410	2.031005
8	6	0	4.432755	-0.313527	1.630943
9	8	0	5.975398	-0.076915	-0.135173
10	8	0	5.355669	-0.106469	2.460158
11	6	0	-1.043736	-0.708488	-1.887231
12	6	0	-1.655231	-1.934618	-1.251398
13	6	0	-2.096864	-1.891679	0.077350
14	6	0	-1.818029	-3.123589	-2.012680
15	6	0	-2.688704	-3.003389	0.658300
16	6	0	-2.394513	-4.238904	-1.467548
17	6	0	-2.861076	-4.250717	-0.103503
18	8	0	-3.390506	-5.263449	0.421509

19	6	0	0.437813	1.198969	-1.603285
20	6	0	-0.595696	2.238584	-1.229735
21	6	0	-0.814042	2.560304	0.120756
22	6	0	-1.325197	2.911724	-2.208497
23	6	0	-1.746891	3.527777	0.474510
24	6	0	-2.263092	3.888586	-1.859902
25	6	0	-2.478282	4.197616	-0.526421
26	8	0	-3.400191	5.156274	-0.191463
27	8	0	-2.042354	3.923109	1.752020
28	6	0	-1.332140	3.317789	2.840797
29	6	0	6.364503	-0.051485	-1.522495
30	8	0	-3.152260	-3.063655	1.912325
31	6	0	-3.048120	-1.903378	2.759386
32	1	0	0.927588	-2.083597	-1.109212
33	1	0	1.680863	-1.027543	-2.303767
34	1	0	3.876416	-0.530671	-1.791145
35	1	0	1.069809	-0.977995	1.434022
36	1	0	2.864373	-0.580481	3.095814
37	1	0	-1.810389	0.072326	-1.919576
38	1	0	-0.775090	-0.934245	-2.931316
39	1	0	-1.975529	-0.977358	0.642280
40	1	0	-1.471152	-3.137398	-3.040289
41	1	0	-2.516717	-5.152557	-2.038778
42	1	0	1.393666	1.466271	-1.144894
43	1	0	0.591488	1.215995	-2.694627
44	1	0	-0.245119	2.051329	0.888401
45	1	0	-1.165836	2.678255	-3.255823
46	1	0	-2.832081	4.413284	-2.619638
47	1	0	-3.422578	5.249199	0.772998
48	1	0	-0.259291	3.513981	2.762506
49	1	0	-1.725010	3.783840	3.743165
50	1	0	-1.514832	2.240301	2.874149
51	1	0	5.839406	0.746961	-2.051313
52	1	0	7.434272	0.146662	-1.517005
53	1	0	6.165158	-1.016948	-1.992648
54	1	0	-3.609661	-1.068656	2.334174
55	1	0	-3.486064	-2.200817	3.709988
56	1	0	-2.001124	-1.626404	2.900664

3rd PA

Sum of electronic and thermal Enthalpies= -1435.087755

Frequencies -- 17.8242 19.0384 22.2191

Center Number	Atomic Number	Atomic Type	Coordinates (Angstroms) X	Y	Z
1	7	0	0.104368	-0.073555	-1.099779
2	6	0	1.168672	-1.079299	-1.234166
3	6	0	2.274926	-0.916409	-0.224503
4	6	0	3.603108	-0.795178	-0.643046
5	6	0	1.971650	-0.926847	1.167188
6	6	0	4.630611	-0.680860	0.287021
7	6	0	2.959803	-0.815915	2.105010

8	6	0	4.345063	-0.684865	1.727802
9	8	0	5.929803	-0.555235	-0.012127
10	8	0	5.272292	-0.583258	2.574174
11	6	0	-1.111164	-0.480468	-1.851286
12	6	0	-1.789047	-1.713464	-1.312768
13	6	0	-2.433857	-1.688096	-0.057849
14	6	0	-1.814399	-2.908102	-2.034687
15	6	0	-3.061869	-2.813742	0.451910
16	6	0	-2.445191	-4.046381	-1.530072
17	6	0	-3.091534	-4.063597	-0.269196
18	8	0	-3.677019	-5.116335	0.219732
19	6	0	0.574006	1.242175	-1.560848
20	6	0	-0.324273	2.388963	-1.164601
21	6	0	-0.704096	2.562763	0.173450
22	6	0	-0.743781	3.328456	-2.144331
23	6	0	-1.488573	3.643107	0.547289
24	6	0	-1.519519	4.404822	-1.806513
25	6	0	-1.940210	4.628076	-0.446922
26	8	0	-2.654911	5.608478	-0.111186
27	8	0	-1.900189	3.904866	1.795374
28	6	0	-1.521010	3.017068	2.863528
29	6	0	6.339579	-0.539697	-1.392413
30	8	0	-3.709059	-2.848243	1.669245
31	6	0	-3.736436	-1.658378	2.457241
32	1	0	0.706074	-2.059698	-1.074955
33	1	0	1.592175	-1.087288	-2.251496
34	1	0	3.821088	-0.788008	-1.703535
35	1	0	0.938423	-1.034623	1.475907
36	1	0	2.738331	-0.832467	3.166667
37	1	0	-1.797280	0.371593	-1.808372
38	1	0	-0.863650	-0.636833	-2.914728
39	1	0	-2.432944	-0.765174	0.510070
40	1	0	-1.331629	-2.954488	-3.007277
41	1	0	-2.450937	-4.967119	-2.107223
42	1	0	1.558711	1.420367	-1.114334
43	1	0	0.716242	1.254725	-2.653551
44	1	0	-0.373178	1.843524	0.910267
45	1	0	-0.442169	3.178041	-3.175432
46	1	0	-1.848837	5.122765	-2.549640
47	1	0	-0.434149	2.987854	2.969104
48	1	0	-1.970821	3.437202	3.760862
49	1	0	-1.912576	2.013252	2.684165
50	1	0	5.891165	0.308042	-1.915304
51	1	0	7.422120	-0.432100	-1.370126
52	1	0	6.067505	-1.477030	-1.883015
53	1	0	-4.252189	-0.845917	1.934957
54	1	0	-4.288481	-1.912734	3.362115
55	1	0	-2.725670	-1.335977	2.728213

3rd ETE (0,4)

Sum of electronic and thermal Enthalpies= -1434.927377

Frequencies -- 13.3380 15.0782 16.7695

Center Number	Atomic Number	Atomic Type	Coordinates (Angstroms) X	Y	Z
1	7	0	0.096885	-0.132541	-1.167368
2	6	0	1.180237	-1.122593	-1.297234
3	6	0	2.282707	-0.937696	-0.282902
4	6	0	3.605691	-0.789726	-0.701847
5	6	0	1.975921	-0.955722	1.108568
6	6	0	4.631032	-0.654387	0.230428
7	6	0	2.959027	-0.825248	2.047979
8	6	0	4.342561	-0.665411	1.672007
9	8	0	5.924787	-0.501917	-0.066065
10	8	0	5.265031	-0.544692	2.518394
11	6	0	-1.095582	-0.563679	-1.919648
12	6	0	-1.815460	-1.736166	-1.294182
13	6	0	-2.302028	-1.642958	0.015470
14	6	0	-2.031664	-2.921212	-2.047964
15	6	0	-2.992611	-2.702704	0.585967
16	6	0	-2.702853	-3.986932	-1.512264
17	6	0	-3.219491	-3.946964	-0.167015
18	8	0	-3.835223	-4.913571	0.350049
19	6	0	0.547100	1.195000	-1.623584
20	6	0	-0.369038	2.321420	-1.204598
21	6	0	-0.641466	2.536260	0.152109
22	6	0	-0.926761	3.185977	-2.184785
23	6	0	-1.457603	3.586119	0.547860
24	6	0	-1.739287	4.227895	-1.827758
25	6	0	-2.058173	4.490017	-0.446452
26	8	0	-2.806130	5.436857	-0.093171
27	8	0	-1.770318	3.885312	1.813647
28	6	0	-1.230835	3.084660	2.882804
29	6	0	6.340063	-0.473330	-1.445773
30	8	0	-3.510165	-2.712438	1.819316
31	6	0	-3.370361	-1.546715	2.653849
32	1	0	0.742936	-2.113940	-1.138710
33	1	0	1.605322	-1.120049	-2.312810
34	1	0	3.824381	-0.777385	-1.762045
35	1	0	0.944447	-1.083880	1.415150
36	1	0	2.736947	-0.846132	3.109316
37	1	0	-1.790407	0.281533	-1.950085
38	1	0	-0.840722	-0.805366	-2.963023
39	1	0	-2.139741	-0.730603	0.573467
40	1	0	-1.649668	-2.972479	-3.061824
41	1	0	-2.866613	-4.898069	-2.077033
42	1	0	1.532013	1.378999	-1.182944
43	1	0	0.677464	1.216447	-2.716535
44	1	0	-0.201082	1.877511	0.888450
45	1	0	-0.702156	3.004263	-3.230306
46	1	0	-2.175015	4.888836	-2.568871
47	1	0	-0.139640	3.132479	2.881527
48	1	0	-1.622232	3.524898	3.797543
49	1	0	-1.567267	2.049538	2.792411
50	1	0	5.876676	0.367717	-1.965988
51	1	0	7.419983	-0.344561	-1.417762

52	1	0	6.087738	-1.414195	-1.939748
53	1	0	-3.859685	-0.686356	2.192011
54	1	0	-3.867326	-1.800343	3.587961
55	1	0	-2.315160	-1.332885	2.837848

S3.2 - Compound 2

2nd PA (-1,2)

Sum of electronic and thermal Enthalpies= -1664.754790

Frequencies -- 12.0172 17.0052 17.6699

Center Number	Atomic Number	Atomic Type	Coordinates (Angstroms) X	Y	Z
1	7	0	-0.041943	0.093583	-1.192085
2	6	0	0.323369	-1.332014	-1.382855
3	6	0	1.565658	-1.767304	-0.641745
4	6	0	2.743332	-2.078809	-1.331466
5	6	0	1.556198	-1.885297	0.755303
6	6	0	3.885563	-2.488521	-0.642961
7	6	0	2.697052	-2.294310	1.440957
8	6	0	3.922003	-2.614377	0.777090
9	8	0	5.080383	-2.808488	-1.266627
10	8	0	4.988659	-2.997467	1.423388
11	6	0	-1.396135	0.343986	-1.704522
12	6	0	-2.490898	-0.256305	-0.852992
13	6	0	-2.553406	0.059071	0.520125
14	6	0	-3.460620	-1.081466	-1.448107
15	6	0	-3.570781	-0.443687	1.302699
16	6	0	-4.492916	-1.602877	-0.689604
17	6	0	-4.604935	-1.311376	0.738494
18	8	0	-5.535762	-1.777982	1.439273
19	6	0	0.936954	0.985577	-1.844176
20	6	0	0.822712	2.434797	-1.422147
21	6	0	1.161001	2.809849	-0.110640
22	6	0	0.407056	3.420180	-2.316448
23	6	0	1.075082	4.138003	0.289341
24	6	0	0.318989	4.759141	-1.920937
25	6	0	0.647472	5.120875	-0.624548
26	8	0	0.557701	6.435188	-0.239336
27	8	0	1.381874	4.619459	1.534861
28	6	0	1.828084	3.695932	2.537044
29	6	0	5.144049	-2.724602	-2.689109
30	8	0	-3.734244	-0.205703	2.618078
31	6	0	-2.786050	0.640538	3.289773
32	1	0	-0.531730	-1.919664	-1.035687
33	1	0	0.442384	-1.551829	-2.457753
34	1	0	2.757474	-1.996028	-2.411525
35	1	0	0.646661	-1.656869	1.297256
36	1	0	-1.548256	1.428317	-1.724777
37	1	0	-1.504992	-0.011096	-2.742567
38	1	0	-1.794942	0.702764	0.943224
39	1	0	-3.382044	-1.305144	-2.503781
40	1	0	1.929205	0.615612	-1.577396
41	1	0	0.855224	0.920090	-2.942495

42	1	0	1.496222	2.054284	0.588709
43	1	0	0.148031	3.149643	-3.334620
44	1	0	-0.006157	5.526728	-2.614822
45	1	0	0.824741	6.507743	0.689547
46	1	0	2.754596	3.204029	2.228727
47	1	0	2.010212	4.294320	3.428497
48	1	0	1.059028	2.947587	2.746665
49	1	0	4.960419	-1.703345	-3.039502
50	1	0	6.158000	-3.019834	-2.960215
51	1	0	4.430931	-3.407134	-3.163424
52	1	0	-2.789226	1.643045	2.854719
53	1	0	-3.119251	0.685082	4.324961
54	1	0	-1.782707	0.210573	3.240401
55	8	0	2.753306	-2.435557	2.816588
56	6	0	1.575095	-2.163287	3.573553
57	1	0	0.754451	-2.831150	3.291221
58	1	0	1.842062	-2.342380	4.615344
59	1	0	1.255155	-1.122510	3.457002
60	8	0	-5.468286	-2.406163	-1.149548
61	6	0	-5.469998	-2.771387	-2.540564
62	1	0	-4.560544	-3.321372	-2.794468
63	1	0	-6.339637	-3.412759	-2.670230
64	1	0	-5.564342	-1.884053	-3.171195

2nd ETE (0,3)

Sum of electronic and thermal Enthalpies= -1664.602956

Frequencies -- 10.8005 14.4657 18.9856

Center Number	Atomic Number	Atomic Type	Coordinates (Angstroms) X	Y	Z
1	7	0	-0.020051	0.063890	-1.204579
2	6	0	0.386971	-1.341450	-1.376969
3	6	0	1.624780	-1.716078	-0.593364
4	6	0	2.736845	-2.247315	-1.267368
5	6	0	1.630065	-1.563779	0.808460
6	6	0	3.865818	-2.623034	-0.561337
7	6	0	2.736389	-1.935780	1.542438
8	6	0	3.926963	-2.487918	0.893629
9	8	0	4.988582	-3.127005	-1.099485
10	8	0	4.940824	-2.828259	1.548422
11	6	0	-1.389793	0.266448	-1.702688
12	6	0	-2.450819	-0.356685	-0.824870
13	6	0	-2.524116	0.004753	0.536320
14	6	0	-3.373722	-1.255129	-1.385114
15	6	0	-3.506635	-0.527733	1.343643
16	6	0	-4.372514	-1.806437	-0.602251
17	6	0	-4.490446	-1.475225	0.817331
18	8	0	-5.385066	-1.973353	1.541426
19	6	0	0.931595	0.966535	-1.888821
20	6	0	0.761637	2.426548	-1.530951
21	6	0	1.048892	2.870144	-0.228696
22	6	0	0.347663	3.357004	-2.482947

23	6	0	0.916800	4.212736	0.105173
24	6	0	0.215285	4.709794	-2.154577
25	6	0	0.496072	5.140484	-0.867955
26	8	0	0.365724	6.468668	-0.551539
27	8	0	1.170078	4.759230	1.335210
28	6	0	1.624422	3.898768	2.388258
29	6	0	5.058113	-3.306928	-2.525101
30	8	0	-3.679409	-0.252209	2.649493
31	6	0	-2.786184	0.678415	3.284206
32	1	0	-0.436607	-1.970359	-1.023816
33	1	0	0.540393	-1.582982	-2.440607
34	1	0	2.697695	-2.349670	-2.343661
35	1	0	0.753207	-1.159817	1.295077
36	1	0	-1.571394	1.345278	-1.734937
37	1	0	-1.500217	-0.107037	-2.733147
38	1	0	-1.804480	0.708855	0.930115
39	1	0	-3.288018	-1.510421	-2.432955
40	1	0	1.936858	0.645050	-1.603955
41	1	0	0.857389	0.850426	-2.982559
42	1	0	1.382423	2.158030	0.515402
43	1	0	0.126062	3.032214	-3.493942
44	1	0	-0.106493	5.435435	-2.893569
45	1	0	0.613235	6.596592	0.376737
46	1	0	2.576584	3.430341	2.124562
47	1	0	1.760426	4.542332	3.256086
48	1	0	0.878823	3.131216	2.612810
49	1	0	4.948218	-2.348916	-3.038955
50	1	0	6.046937	-3.719198	-2.716353
51	1	0	4.290894	-4.008252	-2.861577
52	1	0	-2.854944	1.660871	2.810716
53	1	0	-3.119473	0.741161	4.318363
54	1	0	-1.757614	0.311657	3.246574
55	8	0	2.851476	-1.849641	2.880230
56	6	0	1.739046	-1.346970	3.639722
57	1	0	0.861712	-1.984034	3.503406
58	1	0	2.060633	-1.376484	4.679030
59	1	0	1.508389	-0.318713	3.350770
60	8	0	-5.304807	-2.674321	-1.028395
61	6	0	-5.301042	-3.077626	-2.409315
62	1	0	-4.368957	-3.591414	-2.656621
63	1	0	-6.140550	-3.762368	-2.512607
64	1	0	-5.442516	-2.213142	-3.062352

3rd PA (-1,3)

Sum of electronic and thermal Enthalpies= -1664.138477

Frequencies -- 11.3151 16.4248 18.2574

Center Number	Atomic Number	Atomic Type	Coordinates (Angstroms) X	Y	Z
1	7	0	-0.004429	0.082727	-1.157784
2	6	0	0.500693	-1.286639	-1.349127
3	6	0	1.770299	-1.579725	-0.582718

4	6	0	2.901591	-2.052023	-1.269761
5	6	0	1.788732	-1.417719	0.817873
6	6	0	4.059829	-2.357440	-0.578395
7	6	0	2.925463	-1.720687	1.536821
8	6	0	4.136057	-2.207220	0.873800
9	8	0	5.201448	-2.802760	-1.131072
10	8	0	5.178960	-2.482648	1.514758
11	6	0	-1.379306	0.199126	-1.665640
12	6	0	-2.405036	-0.498467	-0.804221
13	6	0	-2.509004	-0.160999	0.561376
14	6	0	-3.269733	-1.444092	-1.380921
15	6	0	-3.463116	-0.761463	1.354693
16	6	0	-4.238746	-2.063053	-0.611965
17	6	0	-4.386064	-1.757511	0.809924
18	8	0	-5.256040	-2.316343	1.521239
19	6	0	0.893237	1.062165	-1.819626
20	6	0	0.589580	2.504755	-1.501829
21	6	0	0.800978	3.014638	-0.203271
22	6	0	0.121489	3.384900	-2.477351
23	6	0	0.546683	4.343682	0.100055
24	6	0	-0.134070	4.725976	-2.181209
25	6	0	0.062256	5.273172	-0.890067
26	8	0	-0.166301	6.522351	-0.600585
27	8	0	0.736906	4.895859	1.351461
28	6	0	1.233535	4.057218	2.393799
29	6	0	5.256036	-2.992095	-2.555698
30	8	0	-3.658726	-0.513613	2.663885
31	6	0	-2.821701	0.456747	3.315650
32	1	0	-0.270947	-1.978867	-0.995965
33	1	0	0.661581	-1.505537	-2.417144
34	1	0	2.852757	-2.164776	-2.344660
35	1	0	0.898245	-1.059636	1.315417
36	1	0	-1.625017	1.266000	-1.689077
37	1	0	-1.458036	-0.169481	-2.701204
38	1	0	-1.835915	0.579991	0.969556
39	1	0	-3.162897	-1.681051	-2.431249
40	1	0	1.908426	0.817320	-1.492369
41	1	0	0.869580	0.919302	-2.913388
42	1	0	1.174522	2.347054	0.564241
43	1	0	-0.046085	3.022550	-3.488306
44	1	0	-0.496856	5.394648	-2.957380
45	1	0	2.225044	3.661720	2.149483
46	1	0	1.306020	4.691026	3.277760
47	1	0	0.549592	3.227035	2.598940
48	1	0	5.081659	-2.047154	-3.076059
49	1	0	6.263487	-3.348459	-2.761796
50	1	0	4.524706	-3.739542	-2.872465
51	1	0	-2.941864	1.440182	2.854653
52	1	0	-3.163189	0.487432	4.348613
53	1	0	-1.774102	0.148062	3.279344
54	8	0	3.054273	-1.619355	2.872954
55	6	0	1.925784	-1.174994	3.644775
56	1	0	1.084634	-1.862515	3.526272
57	1	0	2.263172	-1.177342	4.679518

58	1	0	1.632196	-0.164043	3.351905
59	8	0	-5.115533	-2.980395	-1.056070
60	6	0	-5.077809	-3.363789	-2.441657
61	1	0	-4.116016	-3.820185	-2.688165
62	1	0	-5.875756	-4.094115	-2.561566
63	1	0	-5.263778	-2.500248	-3.084861

3rd ETE (0,4)

Sum of electronic and thermal Enthalpies= -1663.978621

Frequencies -- 12.3754 17.2906 21.0478

Center Number	Atomic Number	Atomic Type	Coordinates (Angstroms) X	Y	Z
1	7	0	0.009379	0.105211	-1.208527
2	6	0	0.411469	-1.305042	-1.360820
3	6	0	1.642918	-1.670438	-0.562788
4	6	0	2.769941	-2.184567	-1.224516
5	6	0	1.628813	-1.519774	0.839147
6	6	0	3.896541	-2.543461	-0.505270
7	6	0	2.731997	-1.875477	1.586029
8	6	0	3.939069	-2.406909	0.950365
9	8	0	5.032277	-3.030785	-1.030073
10	8	0	4.951158	-2.729528	1.616231
11	6	0	-1.355512	0.314450	-1.724256
12	6	0	-2.430271	-0.318649	-0.869545
13	6	0	-2.535495	0.038915	0.490270
14	6	0	-3.332109	-1.223977	-1.452123
15	6	0	-3.530325	-0.504715	1.275363
16	6	0	-4.342964	-1.785901	-0.692286
17	6	0	-4.494484	-1.459093	0.725259
18	8	0	-5.399871	-1.966813	1.428427
19	6	0	0.961371	0.994099	-1.896512
20	6	0	0.826168	2.449402	-1.512651
21	6	0	0.886226	2.832846	-0.167252
22	6	0	0.683531	3.437325	-2.524387
23	6	0	0.805753	4.172043	0.186602
24	6	0	0.597863	4.766110	-2.208195
25	6	0	0.650772	5.215279	-0.839483
26	8	0	0.571832	6.429512	-0.522437
27	8	0	0.863842	4.643718	1.437229
28	6	0	1.030955	3.721606	2.531145
29	6	0	5.120123	-3.213227	-2.454495
30	8	0	-3.732859	-0.235267	2.577614
31	6	0	-2.858082	0.696503	3.235984
32	1	0	-0.418248	-1.923300	-1.004478
33	1	0	0.570661	-1.560599	-2.419862
34	1	0	2.744415	-2.288077	-2.301129
35	1	0	0.739968	-1.130735	1.316219
36	1	0	-1.536473	1.393932	-1.748674
37	1	0	-1.448520	-0.048943	-2.759495
38	1	0	-1.831645	0.748992	0.901434
39	1	0	-3.221214	-1.476356	-2.498340

40	1	0	1.971861	0.669820	-1.627207
41	1	0	0.877689	0.895894	-2.990226
42	1	0	1.005510	2.072871	0.592961
43	1	0	0.638616	3.120941	-3.560889
44	1	0	0.482875	5.525981	-2.973281
45	1	0	1.970343	3.173926	2.427941
46	1	0	1.055397	4.338795	3.426848
47	1	0	0.188351	3.028215	2.577889
48	1	0	5.002412	-2.258040	-2.971837
49	1	0	6.116761	-3.611828	-2.633595
50	1	0	4.366617	-3.926150	-2.797266
51	1	0	-2.921058	1.680907	2.765745
52	1	0	-3.214096	0.752833	4.262872
53	1	0	-1.827399	0.334108	3.219026
54	8	0	2.829929	-1.789781	2.924905
55	6	0	1.701868	-1.303608	3.672063
56	1	0	0.835287	-1.952902	3.525303
57	1	0	2.012026	-1.329507	4.714918
58	1	0	1.460367	-0.278392	3.381235
59	8	0	-5.257652	-2.660837	-1.139988
60	6	0	-5.218811	-3.064284	-2.520414
61	1	0	-4.277740	-3.571904	-2.745312
62	1	0	-6.051035	-3.754653	-2.643047
63	1	0	-5.350587	-2.200809	-3.176731

S3.3 - Compound 3

2nd PA (-1,2)

Sum of electronic and thermal Enthalpies= -1615.784954

Frequencies -- 10.0532 11.9368 13.6666

Center Number	Atomic Number	Atomic Type	Coordinates (Angstroms) X	Y	Z
1	7	0	0.030926	0.074611	-1.196515
2	6	0	1.091372	-0.922186	-1.377821
3	6	0	2.187138	-0.812280	-0.353648
4	6	0	3.539702	-0.776920	-0.761540
5	6	0	1.886449	-0.787638	1.030708
6	6	0	4.548162	-0.736982	0.165382
7	6	0	2.880252	-0.729170	1.969105
8	6	0	4.286377	-0.691181	1.605466
9	8	0	5.178557	-0.587366	2.469637
10	6	0	-1.182801	-0.304229	-1.950188
11	6	0	-1.866643	-1.546546	-1.423772
12	6	0	-2.436451	-1.572422	-0.172136
13	6	0	-1.964650	-2.722463	-2.217796
14	6	0	-3.090293	-2.722555	0.331298
15	6	0	-2.598388	-3.848415	-1.765506
16	6	0	-3.209896	-3.941077	-0.460356
17	8	0	-3.780979	-5.012098	-0.097348
18	6	0	0.510297	1.415926	-1.594617
19	6	0	-0.406862	2.545547	-1.180176
20	6	0	-0.655759	2.789453	0.181093

21	6	0	-0.991975	3.379495	-2.131679
22	6	0	-1.477537	3.840061	0.570996
23	6	0	-1.816264	4.441177	-1.746089
24	6	0	-2.063213	4.673290	-0.402504
25	8	0	-2.874212	5.714597	-0.029292
26	8	0	-1.789814	4.173195	1.862126
27	6	0	-1.254151	3.375616	2.926757
28	1	0	0.639843	-1.915293	-1.260405
29	1	0	1.524082	-0.887517	-2.389945
30	1	0	3.781891	-0.799072	-1.817442
31	1	0	0.849654	-0.821528	1.342059
32	1	0	2.658308	-0.705023	3.029908
33	1	0	-1.869666	0.542651	-1.887899
34	1	0	-0.948599	-0.445943	-3.018388
35	1	0	-2.392826	-0.695816	0.459873
36	1	0	-1.524723	-2.722115	-3.211313
37	1	0	-2.662712	-4.735551	-2.387208
38	1	0	1.487489	1.561101	-1.123988
39	1	0	0.672611	1.463365	-2.683850
40	1	0	-0.200860	2.152838	0.928995
41	1	0	-0.809551	3.204826	-3.186666
42	1	0	-2.274197	5.089948	-2.484797
43	1	0	-2.949730	5.723119	0.936915
44	1	0	-0.161400	3.415588	2.931091
45	1	0	-1.640331	3.814337	3.845552
46	1	0	-1.591253	2.338891	2.843835
47	7	0	5.921983	-0.729478	-0.321009
48	8	0	6.771327	-1.335028	0.324995
49	8	0	6.162765	-0.124687	-1.365474
50	7	0	-3.625465	-2.630597	1.639119
51	8	0	-3.492223	-1.562989	2.286282
52	8	0	-4.221586	-3.598071	2.152155

2nd ETE (0,3)

Sum of electronic and thermal Enthalpies= -1615.595932

Frequencies -- 9.9497 12.4710 14.6275

Center Number	Atomic Number	Atomic Type	Coordinates (Angstroms) X	Y	Z
1	7	0	-0.006753	0.143278	-1.311338
2	6	0	1.266350	-0.547580	-1.535031
3	6	0	2.308244	-0.260996	-0.487202
4	6	0	3.663765	-0.142116	-0.873338
5	6	0	1.972928	-0.169199	0.884418
6	6	0	4.641888	0.037794	0.067166
7	6	0	2.937125	0.032226	1.835246
8	6	0	4.343742	0.157661	1.495705
9	8	0	5.205206	0.390720	2.365577
10	6	0	-1.086288	-0.485252	-2.075783
11	6	0	-1.542511	-1.796111	-1.497770
12	6	0	-1.737851	-1.926668	-0.100630
13	6	0	-1.832359	-2.894777	-2.337110

14	6	0	-2.219373	-3.093267	0.426271
15	6	0	-2.294884	-4.078224	-1.822529
16	6	0	-2.515668	-4.266104	-0.399698
17	8	0	-2.887195	-5.363417	0.060110
18	6	0	0.118977	1.585826	-1.628172
19	6	0	-1.056932	2.415301	-1.168274
20	6	0	-1.300289	2.591493	0.204581
21	6	0	-1.902967	3.033451	-2.088880
22	6	0	-2.370199	3.364712	0.637415
23	6	0	-2.980536	3.813946	-1.660307
24	6	0	-3.218720	3.980988	-0.305336
25	8	0	-4.275999	4.747200	0.108421
26	8	0	-2.701728	3.611084	1.941681
27	6	0	-1.888928	3.042342	2.977622
28	1	0	1.082245	-1.629305	-1.509139
29	1	0	1.679725	-0.330063	-2.532106
30	1	0	3.931975	-0.211339	-1.921021
31	1	0	0.934633	-0.263196	1.175944
32	1	0	2.688217	0.111627	2.887318
33	1	0	-1.949187	0.192941	-2.059156
34	1	0	-0.820574	-0.622481	-3.135255
35	1	0	-1.529367	-1.085482	0.547353
36	1	0	-1.676045	-2.794672	-3.405520
37	1	0	-2.499387	-4.932134	-2.458096
38	1	0	1.026914	1.940720	-1.132773
39	1	0	0.266219	1.729301	-2.710072
40	1	0	-0.643493	2.123523	0.926656
41	1	0	-1.725108	2.910897	-3.151840
42	1	0	-3.640451	4.295594	-2.373437
43	1	0	-4.295362	4.764110	1.077477
44	1	0	-0.860573	3.407430	2.909074
45	1	0	-2.333843	3.372757	3.914888
46	1	0	-1.903603	1.950217	2.927872
47	7	0	6.023498	0.124374	-0.391823
48	8	0	6.897966	-0.383782	0.301835
49	8	0	6.242986	0.693372	-1.460058
50	7	0	-2.439978	-3.151186	1.867261
51	8	0	-1.654651	-2.546862	2.596873
52	8	0	-3.402009	-3.788112	2.283429

3rd PA (-1,3)
Sum of electronic and thermal Enthalpies= -1615.161551
Frequencies -- 13.2360 13.8531 18.0198

Center Number	Atomic Number	Atomic Type	Coordinates (Angstroms) X	Y	Z
1	7	0	0.085861	0.158556	-1.239374
2	6	0	1.133702	-0.854103	-1.404216
3	6	0	2.195141	-0.785115	-0.340332
4	6	0	3.561750	-0.811612	-0.699283
5	6	0	1.845503	-0.738410	1.031415
6	6	0	4.536223	-0.810645	0.264103

7	6	0	2.806061	-0.718568	2.005797
8	6	0	4.224649	-0.745010	1.693420
9	8	0	5.088985	-0.676281	2.588713
10	6	0	-1.117324	-0.186996	-2.029232
11	6	0	-1.847326	-1.410847	-1.525858
12	6	0	-2.436445	-1.426649	-0.282798
13	6	0	-1.975842	-2.575057	-2.332493
14	6	0	-3.142766	-2.553188	0.198885
15	6	0	-2.659632	-3.679350	-1.900368
16	6	0	-3.296985	-3.759122	-0.606230
17	8	0	-3.916308	-4.808984	-0.263066
18	6	0	0.580433	1.492384	-1.603850
19	6	0	-0.276909	2.632097	-1.105412
20	6	0	-0.879478	2.582857	0.156359
21	6	0	-0.417791	3.794925	-1.912596
22	6	0	-1.615820	3.661955	0.626014
23	6	0	-1.137019	4.874063	-1.477959
24	6	0	-1.781032	4.875773	-0.188200
25	8	0	-2.451796	5.853573	0.232167
26	8	0	-2.224714	3.719163	1.816008
27	6	0	-2.133230	2.596030	2.713909
28	1	0	0.658140	-1.839677	-1.322061
29	1	0	1.600299	-0.810220	-2.400354
30	1	0	3.841428	-0.850954	-1.745327
31	1	0	0.797610	-0.724338	1.304323
32	1	0	2.546968	-0.678306	3.057627
33	1	0	-1.783564	0.678530	-1.983462
34	1	0	-0.855337	-0.330197	-3.090031
35	1	0	-2.369049	-0.558184	0.357556
36	1	0	-1.520099	-2.582894	-3.318762
37	1	0	-2.747482	-4.558244	-2.530763
38	1	0	1.576745	1.617289	-1.162746
39	1	0	0.712931	1.589072	-2.693461
40	1	0	-0.763346	1.692340	0.757992
41	1	0	0.053338	3.813205	-2.889672
42	1	0	-1.253709	5.762041	-2.089454
43	1	0	-1.091190	2.401824	2.977268
44	1	0	-2.692373	2.890395	3.599697
45	1	0	-2.585663	1.708914	2.265418
46	7	0	5.926284	-0.865404	-0.170942
47	8	0	6.723564	-1.504137	0.508148
48	8	0	6.231421	-0.276113	-1.207401
49	7	0	-3.695361	-2.449822	1.499251
50	8	0	-3.524265	-1.395735	2.159293
51	8	0	-4.344175	-3.393534	1.990789

3rd ETE (0,4)
Sum of electronic and thermal Enthalpies= -1614.971377
Frequencies -- 12.9372 17.7792 19.4755

Center Number	Atomic Number	Atomic Type	Coordinates (Angstroms) X	Y	Z

1	7	0	-0.049282	0.378785	-1.380976
2	6	0	1.269155	-0.214275	-1.605223
3	6	0	2.272471	0.063250	-0.517929
4	6	0	3.650771	-0.006157	-0.837661
5	6	0	1.889027	0.339396	0.813252
6	6	0	4.598785	0.166620	0.132207
7	6	0	2.828162	0.539314	1.791373
8	6	0	4.253291	0.473081	1.521836
9	8	0	5.096094	0.705866	2.410102
10	6	0	-1.079039	-0.295217	-2.171912
11	6	0	-1.504294	-1.633062	-1.629605
12	6	0	-1.405283	-1.926486	-0.249901
13	6	0	-2.063924	-2.597797	-2.500939
14	6	0	-1.867386	-3.117994	0.239219
15	6	0	-2.510895	-3.802936	-2.030074
16	6	0	-2.438432	-4.155086	-0.622626
17	8	0	-2.798877	-5.275508	-0.213162
18	6	0	-0.037636	1.825076	-1.624628
19	6	0	-1.167769	2.591735	-0.980398
20	6	0	-1.840194	2.110717	0.145387
21	6	0	-1.505172	3.865764	-1.519596
22	6	0	-2.836793	2.872148	0.745604
23	6	0	-2.476335	4.639121	-0.948569
24	6	0	-3.201428	4.194139	0.215726
25	8	0	-4.100574	4.885358	0.759707
26	8	0	-3.539867	2.506349	1.822376
27	6	0	-3.280043	1.231552	2.441030
28	1	0	1.157771	-1.305093	-1.661435
29	1	0	1.695284	0.089481	-2.575048
30	1	0	3.955350	-0.214236	-1.856791
31	1	0	0.834909	0.395575	1.052497
32	1	0	2.542553	0.763351	2.812803
33	1	0	-1.974843	0.338458	-2.190106
34	1	0	-0.777708	-0.413767	-3.224856
35	1	0	-0.981299	-1.190626	0.420516
36	1	0	-2.130367	-2.373066	-3.559919
37	1	0	-2.922997	-4.554109	-2.693993
38	1	0	0.898210	2.231008	-1.223076
39	1	0	-0.021762	2.049818	-2.703231
40	1	0	-1.574184	1.140315	0.540453
41	1	0	-0.976273	4.217493	-2.399357
42	1	0	-2.739351	5.610308	-1.353029
43	1	0	-2.249655	1.184045	2.799813
44	1	0	-3.969361	1.174140	3.280857
45	1	0	-3.478796	0.419108	1.738768
46	7	0	6.001133	0.052234	-0.254189
47	8	0	6.776964	-0.478005	0.533496
48	8	0	6.333881	0.482741	-1.357162
49	7	0	-1.779186	-3.342419	1.678501
50	8	0	-0.796133	-2.901156	2.271804
51	8	0	-2.693785	-3.945623	2.229382

S3.4 - Compound 4

2nd PA (-1,2)

Sum of electronic and thermal Enthalpies= -2125.928782

Frequencies -- 14.7043 17.4146 21.1402

Center Number	Atomic Number	Atomic Type	Coordinates (Angstroms) X	Y	Z
1	7	0	0.047604	-0.050162	-1.230154
2	6	0	1.079163	-1.074912	-1.431475
3	6	0	2.217947	-0.966025	-0.453803
4	6	0	3.548104	-0.894207	-0.910892
5	6	0	1.963232	-0.975284	0.943979
6	6	0	4.589594	-0.826966	-0.015960
7	6	0	2.985878	-0.912224	1.847408
8	6	0	4.370821	-0.830112	1.428041
9	8	0	5.310682	-0.770550	2.254851
10	6	0	-1.213994	-0.429172	-1.908055
11	6	0	-1.901000	-1.629662	-1.303014
12	6	0	-2.491793	-1.553003	-0.033780
13	6	0	-1.994340	-2.853979	-1.980220
14	6	0	-3.126476	-2.651037	0.524330
15	6	0	-2.633961	-3.951779	-1.419818
16	6	0	-3.239382	-3.923393	-0.126260
17	8	0	-3.828152	-4.950044	0.389692
18	6	0	0.526165	1.269334	-1.691614
19	6	0	-0.323164	2.430992	-1.221300
20	6	0	-0.430049	2.712617	0.151126
21	6	0	-0.982879	3.255888	-2.130549
22	6	0	-1.184914	3.792030	0.594455
23	6	0	-1.741542	4.345893	-1.691764
24	6	0	-1.846156	4.616449	-0.336931
25	8	0	-2.592178	5.687466	0.087721
26	8	0	-1.358608	4.162821	1.901902
27	6	0	-0.717608	3.391058	2.926433
28	1	0	0.604368	-2.051405	-1.277224
29	1	0	1.469479	-1.068808	-2.461754
30	1	0	3.745347	-0.886423	-1.976706
31	1	0	0.938049	-1.042775	1.287886
32	1	0	2.802087	-0.930256	2.915951
33	1	0	-1.872655	0.440604	-1.846804
34	1	0	-1.032678	-0.616400	-2.980156
35	1	0	-2.455109	-0.622326	0.522739
36	1	0	-1.555048	-2.947260	-2.970002
37	1	0	-2.689468	-4.887937	-1.968264
38	1	0	1.542259	1.397819	-1.307775
39	1	0	0.597310	1.295513	-2.791704
40	1	0	0.082758	2.080941	0.865111
41	1	0	-0.910943	3.052824	-3.193780
42	1	0	-2.256862	4.988274	-2.397578
43	1	0	-2.556884	5.731862	1.055291
44	1	0	0.369184	3.412418	2.807458
45	1	0	-0.992154	3.865799	3.867286
46	1	0	-1.075308	2.357903	2.917325

47	17	0	-3.853874	-2.467486	2.138597
48	17	0	6.234195	-0.727379	-0.590823

2nd ETE (0,3)

Sum of electronic and thermal Enthalpies= -2125.757521

Frequencies -- 10.3450 17.1941 18.1255

Center Number	Atomic Number	Atomic Type	Coordinates (Angstroms) X	Y	Z
1	7	0	0.109760	-0.077123	-1.266066
2	6	0	1.174356	-1.073576	-1.453044
3	6	0	2.276139	-0.963105	-0.429574
4	6	0	3.622140	-0.925781	-0.840573
5	6	0	1.967603	-0.943060	0.956812
6	6	0	4.631443	-0.862753	0.090811
7	6	0	2.956714	-0.882784	1.896771
8	6	0	4.357853	-0.833519	1.526433
9	8	0	5.266913	-0.771965	2.384564
10	6	0	-1.106429	-0.468716	-1.987171
11	6	0	-1.845728	-1.612389	-1.342244
12	6	0	-2.113115	-1.589854	0.044954
13	6	0	-2.315573	-2.695361	-2.124073
14	6	0	-2.815173	-2.610594	0.630706
15	6	0	-3.016107	-3.725158	-1.555579
16	6	0	-3.309133	-3.753014	-0.136604
17	8	0	-3.943576	-4.695855	0.388610
18	6	0	0.571574	1.268100	-1.677189
19	6	0	-0.333758	2.389052	-1.219378
20	6	0	-0.440892	2.690906	0.149143
21	6	0	-1.054454	3.152367	-2.136696
22	6	0	-1.256754	3.729512	0.580413
23	6	0	-1.877041	4.199454	-1.709578
24	6	0	-1.982784	4.489291	-0.358798
25	8	0	-2.789520	5.516589	0.056562
26	8	0	-1.436645	4.119364	1.880502
27	6	0	-0.729058	3.419508	2.913114
28	1	0	0.729579	-2.069583	-1.345135
29	1	0	1.601661	-1.026387	-2.466461
30	1	0	3.857229	-0.940613	-1.898494
31	1	0	0.929010	-0.983484	1.261890
32	1	0	2.733972	-0.875658	2.957892
33	1	0	-1.780907	0.395668	-1.996383
34	1	0	-0.896962	-0.714737	-3.039773
35	1	0	-1.762932	-0.755565	0.639164
36	1	0	-2.105273	-2.703783	-3.188090
37	1	0	-3.373004	-4.563414	-2.143377
38	1	0	1.564919	1.410445	-1.243164
39	1	0	0.689936	1.318727	-2.771304
40	1	0	0.122895	2.110871	0.868750
41	1	0	-0.977927	2.935463	-3.196779
42	1	0	-2.439553	4.794277	-2.420870
43	1	0	-2.745815	5.577730	1.022992

44	1	0	0.351713	3.510731	2.774401
45	1	0	-1.020424	3.899287	3.846224
46	1	0	-1.016840	2.364942	2.936016
47	17	0	-3.158815	-2.572655	2.341122
48	17	0	6.295829	-0.804260	-0.422028

3rd PA (-1,3)
Sum of electronic and thermal Enthalpies= -2125.305961
Frequencies -- 15.5002 17.5003 20.5369

Center Number	Atomic Number	Atomic Type	Coordinates (Angstroms) X	Y	Z
1	7	0	0.061054	-0.038196	-1.195387
2	6	0	1.066059	-1.091412	-1.399377
3	6	0	2.248556	-0.955398	-0.479229
4	6	0	3.554410	-0.876005	-0.999750
5	6	0	2.059626	-0.932956	0.928545
6	6	0	4.635151	-0.767225	-0.156371
7	6	0	3.122188	-0.830202	1.781237
8	6	0	4.484126	-0.734086	1.295461
9	8	0	5.460267	-0.632766	2.075406
10	6	0	-1.235257	-0.409726	-1.813314
11	6	0	-1.907491	-1.599114	-1.176237
12	6	0	-2.373966	-1.542881	0.145780
13	6	0	-2.124224	-2.790761	-1.884531
14	6	0	-3.012683	-2.628565	0.720208
15	6	0	-2.772241	-3.874368	-1.309448
16	6	0	-3.261467	-3.863224	0.033537
17	8	0	-3.867611	-4.869772	0.562517
18	6	0	0.533284	1.247786	-1.729398
19	6	0	-0.257875	2.441692	-1.250296
20	6	0	-0.562651	2.595338	0.108152
21	6	0	-0.644721	3.445923	-2.178945
22	6	0	-1.241424	3.719675	0.553664
23	6	0	-1.313788	4.567606	-1.770070
24	6	0	-1.652759	4.775064	-0.384745
25	8	0	-2.265123	5.798130	0.017270
26	8	0	-1.577898	3.963527	1.826529
27	6	0	-1.225054	3.008419	2.845148
28	1	0	0.578571	-2.049135	-1.181642
29	1	0	1.407802	-1.135236	-2.445348
30	1	0	3.702110	-0.894019	-2.073437
31	1	0	1.053295	-1.008887	1.322966
32	1	0	2.988377	-0.823268	2.857312
33	1	0	-1.880058	0.470412	-1.727048
34	1	0	-1.103719	-0.603277	-2.890858
35	1	0	-2.236672	-0.638769	0.729030
36	1	0	-1.777994	-2.866101	-2.911918
37	1	0	-2.927798	-4.783995	-1.882463
38	1	0	1.570754	1.387637	-1.404542
39	1	0	0.549258	1.242336	-2.831119
40	1	0	-0.258365	1.826204	0.804417

41	1	0	-0.403703	3.308902	-3.227775
42	1	0	-1.616102	5.335988	-2.473130
43	1	0	-0.140182	2.894427	2.902497
44	1	0	-1.605348	3.425907	3.775203
45	1	0	-1.699268	2.044696	2.646708
46	17	0	-3.570548	-2.486509	2.402086
47	17	0	6.248793	-0.652132	-0.810776

3rd ETE (0,4)

Sum of electronic and thermal Enthalpies= -2125.133321

Frequencies -- 14.1092 16.0226 19.8566

Center Number	Atomic Number	Atomic Type	Coordinates (Angstroms) X	Y	Z
1	7	0	0.156880	-0.066532	-1.262032
2	6	0	1.215293	-1.070335	-1.437821
3	6	0	2.383973	-0.880872	-0.503707
4	6	0	3.698018	-0.972884	-1.000860
5	6	0	2.174838	-0.661652	0.883516
6	6	0	4.772097	-0.853258	-0.151586
7	6	0	3.230054	-0.540283	1.742915
8	6	0	4.602158	-0.627724	1.282557
9	8	0	5.571816	-0.519836	2.066583
10	6	0	-1.097391	-0.509830	-1.876858
11	6	0	-1.825065	-1.582693	-1.107880
12	6	0	-1.769000	-1.630337	0.300028
13	6	0	-2.622278	-2.524499	-1.806926
14	6	0	-2.477910	-2.582275	0.988362
15	6	0	-3.334456	-3.480904	-1.137408
16	6	0	-3.305997	-3.576724	0.308838
17	8	0	-3.948469	-4.455446	0.927075
18	6	0	0.572847	1.238862	-1.786862
19	6	0	-0.238157	2.409591	-1.284062
20	6	0	-0.791561	2.415935	-0.000355
21	6	0	-0.380917	3.549163	-2.124264
22	6	0	-1.476048	3.532530	0.463342
23	6	0	-1.050562	4.662015	-1.697191
24	6	0	-1.639700	4.724174	-0.382810
25	8	0	-2.263557	5.734202	0.032877
26	8	0	-2.032131	3.646227	1.674510
27	6	0	-1.929328	2.553477	2.607580
28	1	0	0.785775	-2.056334	-1.225055
29	1	0	1.573778	-1.103760	-2.477818
30	1	0	3.857987	-1.134471	-2.060607
31	1	0	1.159978	-0.598294	1.255999
32	1	0	3.083076	-0.382002	2.805462
33	1	0	-1.770500	0.354302	-1.939077
34	1	0	-0.944693	-0.850176	-2.913494
35	1	0	-1.160321	-0.908766	0.828821
36	1	0	-2.656133	-2.480830	-2.890246
37	1	0	-3.941677	-4.209599	-1.662576
38	1	0	1.612579	1.409381	-1.482982

39	1	0	0.573579	1.248310	-2.888691
40	1	0	-0.676343	1.541008	0.623987
41	1	0	0.050559	3.522507	-3.119305
42	1	0	-1.167188	5.533216	-2.332335
43	1	0	-0.881859	2.341871	2.833518
44	1	0	-2.442699	2.892757	3.504913
45	1	0	-2.421907	1.663717	2.209417
46	17	0	-2.418029	-2.630012	2.730403
47	17	0	6.396278	-0.961235	-0.771376

S3.5 - Compound 5

2nd PA (-1,2)

Sum of electronic and thermal Enthalpies= -6353.767778

Frequencies -- 13.6037 15.0954 20.4955

Center Number	Atomic Number	Atomic Type	Coordinates (Angstroms) X	Y	Z
1	7	0	-0.043640	0.193451	1.302274
2	6	0	-1.013453	-0.925190	1.386187
3	6	0	-2.046645	-0.930965	0.287454
4	6	0	-3.408320	-0.774315	0.571071
5	6	0	-1.689614	-1.118283	-1.058643
6	6	0	-4.363129	-0.796143	-0.438850
7	6	0	-2.640584	-1.132527	-2.064471
8	6	0	-4.045546	-0.964186	-1.825088
9	8	0	-4.904470	-0.971568	-2.783500
10	6	0	1.078542	-0.035707	2.217514
11	6	0	1.997580	-1.147337	1.783527
12	6	0	2.478427	-1.192564	0.454762
13	6	0	2.432133	-2.126959	2.708015
14	6	0	3.347850	-2.177053	0.061006
15	6	0	3.300455	-3.117063	2.332903
16	6	0	3.815788	-3.211944	0.980849
17	8	0	4.606680	-4.122583	0.643029
18	6	0	-0.690052	1.491615	1.578319
19	6	0	0.146182	2.685965	1.171114
20	6	0	0.404697	2.926110	-0.189242
21	6	0	0.653097	3.573709	2.118806
22	6	0	1.156942	4.026307	-0.582456
23	6	0	1.410236	4.684002	1.730084
24	6	0	1.665346	4.912464	0.387633
25	8	0	2.407693	6.003777	0.011095
26	8	0	1.467601	4.361891	-1.873919
27	6	0	0.981444	3.529480	-2.935925
28	1	0	-0.425473	-1.848268	1.339476
29	1	0	-1.519504	-0.923307	2.366403
30	1	0	-3.723985	-0.633581	1.599594
31	1	0	-0.644372	-1.257999	-1.319698
32	1	0	-2.338153	-1.280855	-3.097705
33	1	0	1.671996	0.885904	2.255695
34	1	0	0.734801	-0.230138	3.246807
35	1	0	2.149761	-0.434766	-0.244772

36	1	0	2.060615	-2.088529	3.726402
37	1	0	3.632568	-3.875460	3.033279
38	1	0	-1.627279	1.505162	1.017610
39	1	0	-0.954225	1.578979	2.645490
40	1	0	0.006784	2.247893	-0.933443
41	1	0	0.459096	3.406266	3.172969
42	1	0	1.806479	5.375582	2.465672
43	1	0	2.486855	6.011014	-0.954931
44	1	0	-0.111682	3.505299	-2.942185
45	1	0	1.343382	3.985547	-3.856206
46	1	0	1.377837	2.514306	-2.847591
47	35	0	3.991176	-2.216336	-1.733204
48	35	0	-6.221110	-0.585091	0.070058

2nd ETE (0,3)

Sum of electronic and thermal Enthalpies= -6353.597965

Frequencies -- 11.7730 15.8548 17.4524

Center Number	Atomic Number	Atomic Type	Coordinates (Angstroms) X	Y	Z
1	7	0	-0.106912	0.227454	1.295342
2	6	0	-1.099737	-0.845476	1.450876
3	6	0	-2.109395	-0.897294	0.332067
4	6	0	-3.483384	-0.994652	0.628951
5	6	0	-1.687716	-0.902933	-1.023267
6	6	0	-4.411961	-1.095069	-0.380325
7	6	0	-2.594196	-1.004636	-2.040231
8	6	0	-4.020589	-1.108290	-1.789210
9	8	0	-4.844957	-1.204846	-2.724787
10	6	0	1.045160	-0.001078	2.172660
11	6	0	1.947629	-1.120061	1.721723
12	6	0	2.279445	-1.267742	0.356352
13	6	0	2.514355	-2.002721	2.673119
14	6	0	3.137309	-2.258715	-0.047960
15	6	0	3.371859	-2.997473	2.289072
16	6	0	3.739327	-3.196183	0.899553
17	8	0	4.519183	-4.111025	0.553486
18	6	0	-0.725244	1.547545	1.552971
19	6	0	0.146382	2.719667	1.162486
20	6	0	0.470280	2.940617	-0.187115
21	6	0	0.619585	3.611704	2.123931
22	6	0	1.255908	4.026043	-0.555443
23	6	0	1.407252	4.707943	1.759820
24	6	0	1.728494	4.917799	0.428346
25	8	0	2.501481	5.993912	0.077195
26	8	0	1.632894	4.342410	-1.832874
27	6	0	1.197595	3.499011	-2.907676
28	1	0	-0.566739	-1.803639	1.450645
29	1	0	-1.621763	-0.775788	2.417702
30	1	0	-3.798649	-0.983783	1.665648
31	1	0	-0.629286	-0.834053	-1.243054
32	1	0	-2.283689	-1.021084	-3.079108

33	1	0	1.644725	0.917353	2.182802
34	1	0	0.736852	-0.180909	3.214876
35	1	0	1.847505	-0.583604	-0.362314
36	1	0	2.253829	-1.884360	3.719395
37	1	0	3.806226	-3.682550	3.008633
38	1	0	-1.653632	1.581496	0.975905
39	1	0	-1.005140	1.642296	2.614455
40	1	0	0.099822	2.260031	-0.943132
41	1	0	0.375023	3.457301	3.169395
42	1	0	1.776570	5.403114	2.505803
43	1	0	2.627898	5.990641	-0.883706
44	1	0	0.106373	3.488213	-2.976298
45	1	0	1.616949	3.936163	-3.812553
46	1	0	1.575767	2.480851	-2.781456
47	35	0	3.585310	-2.437845	-1.890252
48	35	0	-6.263193	-1.209749	0.040820

3rd PA (-1,3)

Sum of electronic and thermal Enthalpies= -6353.144959

Frequencies -- 14.2195 15.3624 16.6257

Center Number	Atomic Number	Atomic Type	Coordinates (Angstroms) X	Y	Z
1	7	0	-0.029856	0.263786	1.253012
2	6	0	-0.960412	-0.861413	1.434725
3	6	0	-2.055600	-0.893163	0.404936
4	6	0	-3.407206	-0.857677	0.799943
5	6	0	-1.742507	-0.988771	-0.976260
6	6	0	-4.414192	-0.908915	-0.136243
7	6	0	-2.729398	-1.046838	-1.920198
8	6	0	-4.135926	-1.008940	-1.565823
9	8	0	-5.034860	-1.063831	-2.437426
10	6	0	1.205799	0.057501	2.049947
11	6	0	2.042514	-1.111747	1.600395
12	6	0	2.698352	-1.093651	0.359495
13	6	0	2.223568	-2.246832	2.406438
14	6	0	3.481430	-2.158959	-0.052433
15	6	0	3.009747	-3.311576	1.996378
16	6	0	3.692163	-3.343264	0.736009
17	8	0	4.413629	-4.341431	0.373989
18	6	0	-0.670867	1.535124	1.623208
19	6	0	0.084066	2.761251	1.169055
20	6	0	0.539431	2.869785	-0.151542
21	6	0	0.284513	3.837114	2.075884
22	6	0	1.188311	4.018591	-0.579086
23	6	0	0.920441	4.983928	1.683912
24	6	0	1.411443	5.146136	0.338975
25	8	0	2.000764	6.189269	-0.045953
26	8	0	1.659758	4.225016	-1.815365
27	6	0	1.506484	3.195564	-2.810358
28	1	0	-0.372889	-1.781640	1.334626
29	1	0	-1.397945	-0.867365	2.445531

30	1	0	-3.643908	-0.783183	1.854771
31	1	0	-0.701911	-1.026863	-1.277241
32	1	0	-2.499001	-1.132870	-2.976504
33	1	0	1.784490	0.982361	1.964740
34	1	0	0.954906	-0.064854	3.116704
35	1	0	2.592259	-0.228977	-0.286299
36	1	0	1.735054	-2.291423	3.376261
37	1	0	3.131861	-4.178758	2.639372
38	1	0	-1.660128	1.561943	1.152204
39	1	0	-0.839320	1.596325	2.710417
40	1	0	0.375933	2.045451	-0.832023
41	1	0	-0.072583	3.734859	3.095147
42	1	0	1.081685	5.806962	2.371476
43	1	0	0.448401	2.992764	-2.990999
44	1	0	1.967991	3.594797	-3.711255
45	1	0	2.021051	2.283658	-2.499431
46	35	0	4.342020	-2.033192	-1.780769
47	35	0	-6.232629	-0.838834	0.427250

3rd ETE (0,4)

Sum of electronic and thermal Enthalpies= -6352.973704

Frequencies -- 11.7802 15.9313 18.3946

Center Number	Atomic Number	Atomic Type	Coordinates (Angstroms) X	Y	Z
1	7	0	-0.137324	0.220463	1.298124
2	6	0	-1.113557	-0.870485	1.443860
3	6	0	-2.210582	-0.835189	0.409279
4	6	0	-3.555774	-0.946308	0.811979
5	6	0	-1.902516	-0.732508	-0.972287
6	6	0	-4.566942	-0.954858	-0.120264
7	6	0	-2.891840	-0.743368	-1.914437
8	6	0	-4.293929	-0.854484	-1.553457
9	8	0	-5.194515	-0.865285	-2.420899
10	6	0	1.076423	-0.061094	2.070369
11	6	0	1.977339	-1.111769	1.472924
12	6	0	2.041274	-1.318135	0.079408
13	6	0	2.818109	-1.868639	2.328008
14	6	0	2.905955	-2.246515	-0.446902
15	6	0	3.687920	-2.794046	1.822609
16	6	0	3.789306	-3.049324	0.397554
17	8	0	4.582449	-3.901206	-0.060405
18	6	0	-0.725747	1.506313	1.686287
19	6	0	0.009010	2.727746	1.188226
20	6	0	0.819957	2.691917	0.051527
21	6	0	-0.189140	3.956476	1.880314
22	6	0	1.433121	3.851295	-0.409434
23	6	0	0.396133	5.113295	1.448351
24	6	0	1.242257	5.134915	0.281147
25	8	0	1.795772	6.185000	-0.135703
26	8	0	2.226673	3.925367	-1.483320
27	6	0	2.496266	2.735394	-2.248661

28	1	0	-0.578432	-1.819321	1.324465
29	1	0	-1.555749	-0.883172	2.451464
30	1	0	-3.783576	-1.018853	1.868841
31	1	0	-0.865494	-0.652591	-1.274473
32	1	0	-2.669119	-0.676003	-2.973550
33	1	0	1.662869	0.863448	2.138424
34	1	0	0.838993	-0.343381	3.108865
35	1	0	1.396607	-0.734783	-0.564632
36	1	0	2.759900	-1.706546	3.399081
37	1	0	4.332508	-3.380086	2.468155
38	1	0	-1.741442	1.553408	1.275835
39	1	0	-0.841399	1.581675	2.780087
40	1	0	0.962334	1.751823	-0.462433
41	1	0	-0.817757	3.962211	2.764713
42	1	0	0.250173	6.052607	1.970277
43	1	0	1.571501	2.333667	-2.668634
44	1	0	3.161095	3.052914	-3.049261
45	1	0	2.991236	1.986007	-1.627252
46	35	0	2.983870	-2.513732	-2.329726
47	35	0	-6.377285	-1.087042	0.445146
